# A universal approach to investigate circRNA protein coding function

**DOI:** 10.1038/s41598-019-48224-y

**Published:** 2019-08-12

**Authors:** Dingding Mo, Xinping Li, Carsten A. Raabe, Di Cui, Jeanne-Franca Vollmar, Timofey S. Rozhdestvensky, Boris V. Skryabin, Juergen Brosius

**Affiliations:** 1 Max Planck Institute for Biology of Ageing, Joseph-Stelzmann-Strasse 9b, 50931 Cologne, Germany; 2 Medical Faculty, Core Facility Transgenic Animal and Genetic Engineering Models (TRAM), University of Münster, Von-Esmarch-Str. 56, D-48149 Münster, Germany; 3 Institute of Experimental Pathology, Centre for Molecular Biology of Inflammation (ZMBE), University of Münster, Von-Esmarch-Str. 56, D-48149 Münster, Germany; 4 Brandenburg Medical School (MHB), Fehrbelliner Strasse 38, D-16816 Neuruppin, Germany; 5 Institute of Medical Biochemistry, Centre for Molecular Biology of Inflammation (ZMBE), University of Münster, Von-Esmarch-Strasse 56, D-48149 Münster, Germany; 6 Institutes for Systems Genetics, West China Hospital, Sichuan University, Chengdu, 610041 China

**Keywords:** RNA, Genetic techniques

## Abstract

Circular RNAs (circRNAs) are an emerging class of RNA molecules that have been linked to human diseases and important regulatory pathways. Their functional roles are still under investigation, often hampered by inefficient circRNA formation *in* and *ex vivo*. We generated an intron-mediated enhancement (IME) system that—in comparison to previously published methods—increases circRNA formation up to 5-fold. This strategy also revealed previously undetected translation of circRNA, e.g., circRtn4. Substantiated by Western blots and mass spectrometry we showed that in mammalian cells, translation of circRtn4 containing a potential “infinite” circular reading frame resulted in “monomers” and extended proteins, presumably “multimer” tandem repeats. In order to achieve high levels of circRNA formation and translation of other natural or recombinant circRNAs, we constructed a versatile circRNA expression vector—pCircRNA-DMo. We demonstrated the general applicability of this method by efficiently generating two additional circRNAs exhibiting high expression levels. The circRNA expression vector will be an important tool to investigate different aspects of circRNA biogenesis and to gain insights into mechanisms of circular RNA translation.

## Introduction

Circular RNAs (circRNAs) are products of hnRNA backsplicing and the resulting RNAs represent covalently closed circles, which are devoid of terminal RNA cap structures and poly(A) tails^1–3^. Circular RNAs might play important regulatory roles in various biological processes, e.g., acting as sponges for sequestering of miRNAs^4–12^. For example, Cdr1as/ciRS-7 interacts with miR-7 and regulates its activity^13–15^. In addition, circRNA functions related to cancer are being increasingly recognized^16–18^. Nevertheless, for most circRNAs, actual functions remain enigmatic^19^.

The analysis of circRNA functions requires effective methods to control RNA formation within cell lines or organisms. However, currently used tools have limitations, thus representing a key challenge in the investigation of circRNAs function, as discussed below^19^. For specific knockdown of circRNAs, only the junction site can be targeted without interruption of its *bona fide* mRNA counterpart, as otherwise they share identical sequences. Thus, knockdown efficiencies are compromised, and off-target effects are anticipated^19^. Furthermore, available vector systems for circRNA formation rely on inverted repeats, which are inserted into introns flanking circularized exons^13,20,21^. Reactions leading to RNA circularization compete with regular splicing events. Therefore, circRNA formation is severely compromised by these undesired side reactions, in particular because RNA circularization is often less efficient compared to the generation of linear isoforms^19,22–24^. In addition, the accumulation of large amounts of linear precursor RNA without efficient splicing may perturb circRNA-specific formation^19,25^. Vector systems that enable increased circRNA formation levels while also limiting side reactions are in high demand. Intron-mediated enhancement (IME) is a conserved eukaryotic mechanism. Incorporation of a specific intron in close proximity to the transcription start sites causes enhanced gene expression levels^26–32^.

The molecular basis of IME remains largely unknown; however, substantially increased cellular mRNA levels from intron-containing genes are reported and IME has been frequently employed as a powerful tool to increase gene expression from linear RNA, especially in applications of transgenesis^31,33^. It is currently unknown whether IME also can serve to enhance circRNA expression. Reticulon-4 or Nogo-A, B, C mRNA is encoded by the *RTN4* gene. The corresponding protein participates in the inhibition of neurite outgrowth within the central nervous system of higher vertebrates, and reportedly inhibits neuronal regeneration after brain injury^34,35^. circRtn4 comprises protein coding exons 2 and 3 of the RTN4 pre-mRNA^4^.

Here we report a superior and universal circRNA formation system based on IME and utilize circRtn4 as a model to monitor the resulting RNA formation in various cell lines^36^. We established that IME-based vector systems provide a means for generally enhancing circRNA formation by testing two additional IME introns^36^. Importantly, we also demonstrated by Western blot and mass spectrometry analysis that IME boosted circRtn4 translation^36^. The results were confirmed with two additional circRNAs, underscoring the value and general applicability of this approach for investigating circRNA functions and their potential translation products^36^.

## Results

### Formation of circRtn4 using an existing method

**Figure 1:**
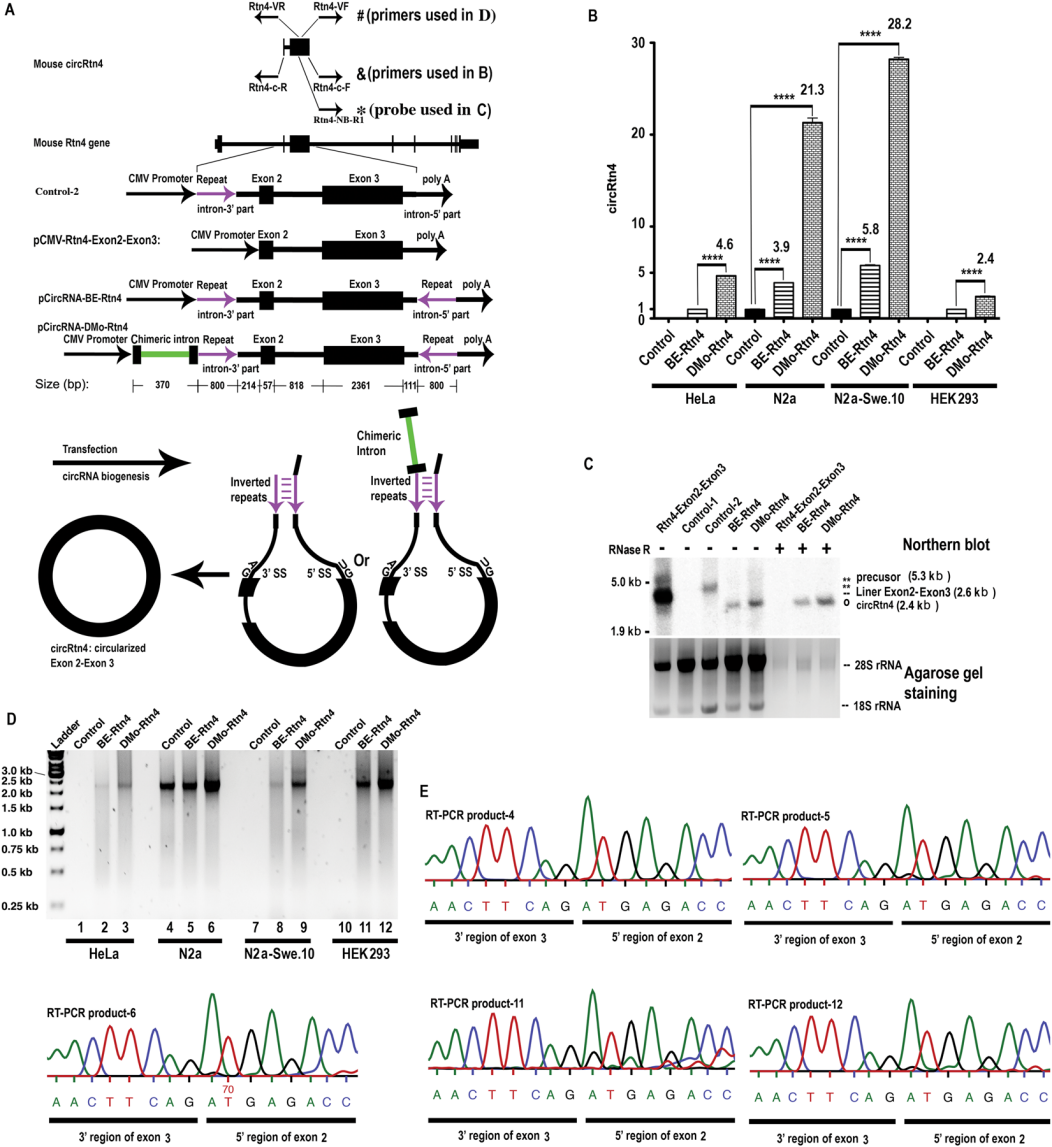
Mouse circRtn4 structure and generation in mammalian cells. (**A**) Schematic representation of the circRtn4 localization within the mouse *Rtn4* gene environment and the circRNA cassette for pCircRNA-BE and pCircRNA-DMo vectors; mouse circRtn4 consists of Rtn4 gene exons 2 and 3. The 800 nt inverted repeats (purple colour) within the flanking introns were inserted to promote backsplicing through the formation of inter-intronic base-pairing interactions; flanking introns lack 5′ and 3′ splice sites, which lead to abolished canonical splicing of exon 2 and exon 3; the chimeric intron is displayed in green; & and # indicate the circRtn4 RT-PCR oligonucleotide positions; &: Rtn4-c-R and Rtn4-c-F were used for qRT-PCR to determine circRtn4 levels (results shown in B); #, Rtn4-VR, Rtn4-VF were used for the analysis of circRtn4 backsplicing fidelity (results shown in C); *indicates the position of the oligonucleotide probe for Northern blot hybridization (Rtn4-NB-R1) as displayed in D. (**B**) circRtn4 levels in transfected cells (HeLa, N2a, N2a-swe.10, HEK293 cell); pCMV-MIR empty vector as negative control; BE-Rtn4, pCircRNA-BE-Rtn4; DMo-Rtn4, pCircRNA-DMo-Rtn4; all T-tests were performed in comparison to levels of the control sample, ****P ≤ *0*.*0001*, *n* ≥ 4; β-actin mRNA was used as internal control. (**C**) Northern blot hybridization for detection of circRtn4 in transfected HEK293 cells. Control-1, pCMV-MIR empty vector; Rtn4-Exon2-Exon3, pCMV-Rtn4-Exon2-Exon3; Control-2, the construct devoid of the downstream portion of the inverted repeat in the 3′ flanking intronic region; BE-Rtn4, pCircRNA-BE-Rtn4; DMo-Rtn4, pCircRNA-DMo-Rtn4; −, no RNase R treatment; +, with RNase R treatment; agarose gel ethidium bromide staining of 28S and 18S rRNAs served as loading control; the weak staining of 18S rRNA is due to co-migration and signal quenching with xylene cyanol loading dye. (**D**) Agarose gel electrophoresis of RT-PCR products of circRtn4 to analyse backsplicing fidelity (PCR primers: Rtn4-VR, Rtn4-VF); pCMV-MIR empty vector as negative control; BE-Rtn4, pCircRNA-BE-Rtn4; DMo-Rtn4, pCircRNA-DMo-Rtn4. The entire un-spliced precursor transcript is ~5.3 kb; the spliced circular isoform of exons 2 and exon 3 devoid of the intron corresponds to 2.4 kb. The intron flanked by exons 2 and 3 is 818 nt long. The expected amplicon size is 2.4 kb. Products migrated between 2.0 and 2.5 kb, indicating that the internal intron was spliced during circRtn4 biogenesis. Lane 1–3, HeLa cells; lane 4–6, N2a cells; lane 7–9, N2a-swe.10 cells; lane 10–12, HEK293 cells. PCR products were sequenced and aligned (data not shown). (**E**) Sequencing of the junction site for circRtn4 backsplicing as revealed by assays in N2a and HEK293 cell lines. The RT-PCR products as displayed in C were sequenced and the junction regions were shown; RT-PCR product-4, 5, 6 were from N2a cells; RT-PCR product-11, 12 were from HEK293 cells.

The mouse circRtn4 cassette was PCR generated as previously described^13^. The resulting construct harboured two 800-nucleotide inverted repeats within the introns flanking Rtn4 exons 2 and 3. The inverted repeats enhance RNA backsplicing, which ultimately leads to the generation of circRtn4. This cassette was inserted into plasmid pCMV-MIR, containing the CMV promoter for transcription (Fig. 1A). The resulting vector, pCircRNA-BE-Rtn4 (BE, Basal Expression), was transfected into the mouse neuroblastoma cell line (N2a) and its derivative, N2a-swe.10. Quantitative RT-PCR with circRtn4-specific oligonucleotides (Rtn4-c-F and Rtn4-c-R, Fig. 1A) revealed a 3.9-fold (N2a) and 5.8-fold (N2a-swe.10) increase in circRtn4 production compared to endogenous (background) levels, as observed in control transfections with empty vector (Fig. 1B). Two additional human cell lines of (notably) non-neuronal origin, i.e., HeLa and HEK293, were transfected with the same plasmid pCircRNA-BE-Rtn4 and also generated detectable levels of circRtn4 (Fig. 1B).

In order to address whether the pCircRNA-BE-Rtn4 cassette would also promote the generation of linear competing RNA isoforms, we performed Northern blot hybridization with a DNA probe specific to both, the linear and circular variants of Rtn4 RNA. For control, we transfected the pCMV-Rtn4-Exon2-Exon3 construct, which serves as template for the linear Rtn4-exon2-exon3 isoform (Fig. 1A). As displayed in Fig. 1C, circRtn4 migrated faster than its linear counterpart in native agarose gels. Importantly, the linear Rtn4-exon2-exon3 fusion product was absent in pCircRNA-BE-Rtn4 transfection experiments (Fig. 1C). This was expected as the vector did not contain functional intron sequences, which are required to promote the regular splicing event. As a negative control, we included the analysis of an analogous construct lacking the 3′ portion of the inverted repeat intron (control-2 plasmid in Fig. 1A,C). The resulting vector did not support the generation of circRNA but instead promoted the formation of the linear precursor RNA (control-2 in Fig. 1A,C), demonstrating that inverted repeats at both 5′ and 3′ sides are required for circRNA biogenesis, which is consistent with previous findings^13,20,21,37^.

Furthermore, we utilized RNase R digestion followed by Northern blot analysis^19,38^ to confirm that the RNA generated from pCircRNA-BE-Rtn4 vector is Rtn4 circular RNA (Fig. 1C). For control, we monitored the effects of RNase R treatment on the linear Rtn4-exon2-exon3 transcript, which was completely digested by the exonuclease activity of RNase R (Fig. 1C). These results confirmed that flanking intronic sequences harbouring inverted repeats promote circRtn4 biogenesis and underscored that the cassette represents a valuable start point for the development of optimized circRNA vector systems.

### A neighbouring chimeric intron significantly enhances circRtn4 biogenesis: intron-mediated enhancement (IME) in circRNA formation

Intron-mediated enhancement (IME) markedly increases expression of protein coding genes. The underlying mechanism of IME in promoting mRNA abundance and translation is not resolved in all details^31,33^. Here, we investigated, whether neighbouring introns might also promote circRNA formation. To address this question, we inserted a β-globin/IgG chimeric intron derived from the pCI-neo expression system, upstream from the circRtn4 cassette under the control of the same CMV promoter. We selected this intron because it is well known to convey strong IME-related effects on mRNA expression (pCI-neo vector). The resulting vector was called pCircRNA-DMo-Rtn4. Comprehensive analysis of transfection experiments in N2a and N2a-swe.10 cell lines using pCircRNA-DMo-Rtn4 and pCircRNA-BE-Rtn4 plasmids, uncovered significant enhancement of circRtn4 levels for the IME–containing vector (Fig. 1B, Table 1). IME-related effects on circRtn4 formation were also investigated with two human cell lines (HeLa and HEK293) and resulted in the detection of a 4.6-fold (HeLa) and 2.4-fold (HEK293) increase in circRtn4 formation compared to pCircRNA-BE-Rtn4 vector (Fig. 1B, Table 1). Interestingly, these data revealed that the chimeric IME intron-containing vector enhanced circRNA formation in various unrelated cell lines (Fig. 1B). Our data established that intron-mediated enhancement significantly boosts circRNA formation.

**Table 1:** Increase of circRtn4 formation *via* IME in various cell lines.

	N2a	N2a-swe.10	HeLa	HEK293
control	1	1	0	0
pCircRNA-BE-Rtn4	3.9	5.8	1	1
pCircRNA-DMo-Rtn4	21.3	28.2	4.6	2.4
fold increase by IME (DMo-Rtn4/BE-Rtn4)	5.5	4.9	4.6	2.4

Summarized from Fig. 1B.

Notably, Northern blot analysis for circRtn4 formation showed that pCircRNA-DMo-Rtn4 did not lead to the production of the linear Rtn4-exon2-exon3 isoform, which is consistent with its design (Fig. 1C). As anticipated, circRtn4 was resistant to RNase R treatment (Fig. 1C).

Moreover, we analysed circRtn4 formation with unique RT-PCR oligonucleotides (Rtn4-VF, Rtn4-VR in Fig. 1A) enabling specific amplification of full-length circRtn4. Agarose gel electrophoresis of the resulting RT-PCR products demonstrated that all circRtn4s were of the same size, predicted to be 2418 nucleotides (Fig. 1D). Sequencing of the corresponding RT-PCR products confirmed that circRtn4 sequences were identical to the wild type circRNA in either human or mouse cell lines (Fig. 1E and data not shown).

### IVS1 and PAT1 introns also boost circRNA biogenesis: ubiquitously robust enhancement by various introns

**Figure 2:**
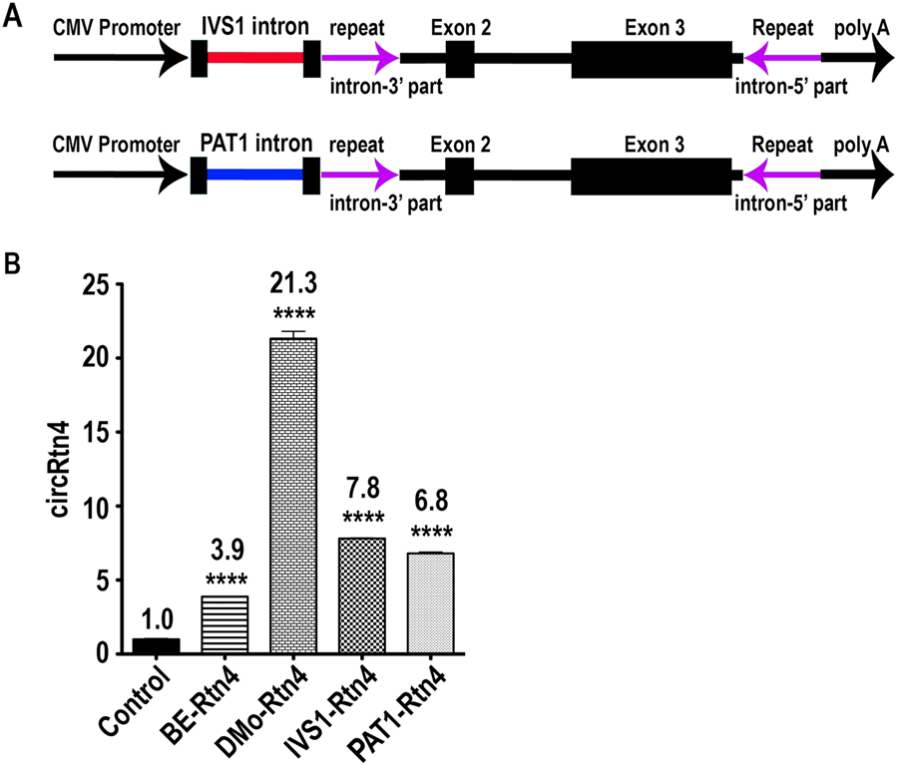
Formation of circRtn4 with IVS1 and PAT1 introns. (**A**) Schematic representation of the circRtn4 cassette for pCircRNA-IVS1-Rtn4 and pCircRNA-PAT1-Rtn4 constructs. The IVS1 intron is displayed in red and the PAT1 intron in blue colour. (**B**) CircRtn4 formation in N2a cells after transfection with circRtn4 constructs. Control, the empty vector control; BE-Rtn4, pCircRNA-BE-Rtn4; DMo-Rtn4, pCircRNA-DMo-Rtn4; IVS1-Rtn4, pCircRNA-IVS1-Rtn4; PAT1-Rtn4, pCircRNA-PAT1-Rtn4; all T-tests were performed in comparison to formation levels of the control sample, ****P ≤ *0*.*0001*, ***P ≤ *0*.*001*, *n* ≥ 4.

Potentially, the effects of IME might be specific to certain combinations of circRNA and intron sequence. To address whether the insertion of intronic sequences upstream of the circRNA has a general effect on formation, we exchanged a chimeric intron in the pCircRNA-DMo-Rtn4 vector with the IVS1 and PAT1 introns^26,32^. Notably, both introns were reported earlier to trigger mRNA expression and hence were selected for our assay^26,32^. The resulting circRtn4 vectors, pCircRNA-IVS1-Rtn4 and pCircRNA-PAT1-Rtn4 (Fig. 2A) were transfected into N2a cells. RT-qPCR analysis of circRtn4 abundance revealed ~2-fold increase in circRNA formation when IVS1 or PAT1 introns were introduced compared to the intron-less pCircRNA-BE-Rtn4 vector (Fig. 2A,B). Based on these results, we concluded that even other introns are capable of boosting circRNA formation, suggesting that IME-related effects generally impact on circRNA biogenesis.

### Protein product from circRtn4

**Figure 3:**
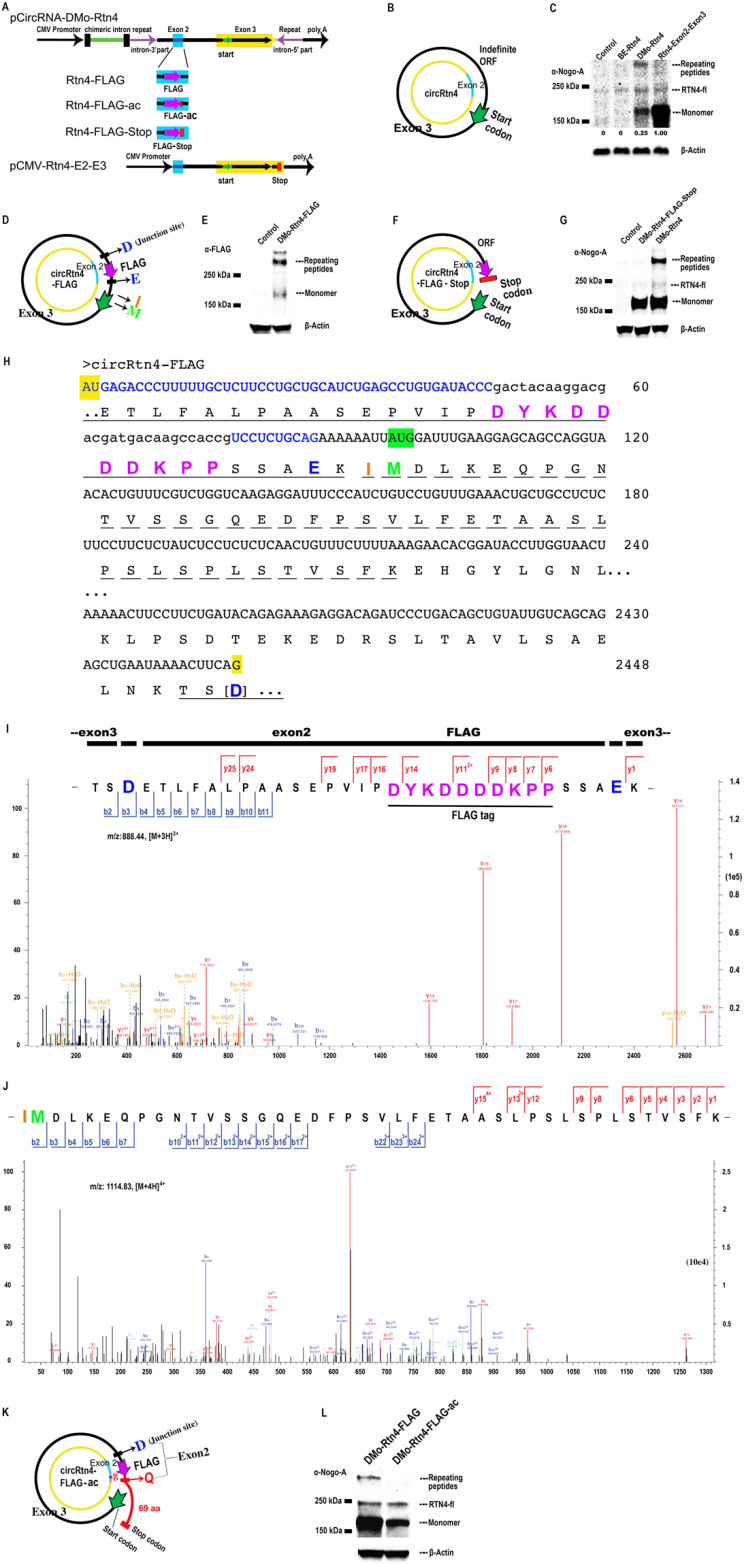
Translation of circRtn4. (**A**) Schematic illustration for the insertion of a FLAG-tag (magenta) with and without stop codon (red) into exon 2 (blue) of the circRtn4 open reading frame. Exons 2 (blue) and 3 (yellow) constitute the circRNA open reading frame; the green arrow, denotes the presumed AUG start codon, magenta arrow denotes the position of the FLAG-tag in pCircRNA-DMo-Rtn4-FLAG and derivatives; the red rectangle denotes the stop codon (UGA) in the pCircRNA-DMo-Rtn4-Stop construct. (**B**) Representation of the “infinite” circRtn4 open reading frame (ORF). The inner circle denotes the circular RNA with exon 2 and exon 3 in blue and yellow. The presumed AUG translation start codon is indicated by a green arrow on the outer circle showing the presumed circRtn4 translation products(s). (**C**) Western blot of circRtn4 translated polypeptides in HEK293 cells detected with antibodies targeting Nogo-A (α-Nogo-A). Control, i.e., the empty vector; BE-Rtn4, pCircRNA-BE-Rtn4; DMo-Rtn4, pCircRNA-DMo-Rtn4; Rtn4-Exon2-Exon3, pCMV-Rtn4-Exon2-Exon3. On the right, polypeptides of higher molecular weights are presumed products of more than one round of circRtn4 circular translation; RTN4-fl indicates the endogenous RTN4 full length protein (Reticulon 4 or Nogo-A); the “monomer” presumably represents a single round of circRtn4 circular translation. The calculated MW of the theoretical “monomer” is 88.2 kDa. Possibly due to its highly acidic pI (4.3) and/or high proline content, it migrates significantly slower in the gel, thus corresponding to 150 kDa^39,40^. For calculating the relative protein level, protein from pCMV-Rtn4-Exon2-Exon3 was set as 1; pCircRNA-DMo-Rtn4 expressed less “monomer” protein by a factor of 0.25. (**D**) The putative open reading frame of Rtn4-FLAG circRNA. Annotation as in B: In addition, the aspartic acid (blue D) is resulting from the junction site of backsplicing; the FLAG-tag peptide is in magenta; the glutamic acid residue (E, highlighted in blue) is resulting from the junction site of exon2-exon3; the green methionine (M) is the presumed start codon and the isoleucine (I, orange) is directly N-terminal to M and supports a further round of translation (see below). (**E**) Western blot hybridisation of pCircRNA-DMo-Rtn4-FLAG expression in N2a cells with the anti-FLAG antibody (α-FLAG). Control, the empty vector; DMo-Rtn4-FLAG, pCircRNA-DMo-Rtn4-FLAG; of note, compared to C, the intensity of repeating peptides is higher than “monomer”; the reason is unknown. (**F**) The putative open reading frame of Rtn4-stop circRNA is delineated. Annotation as in B: In addition, the red bar indicates the in-frame stop codon (UGA). (**G**) Western blot showing expression of pCircRNA-DMo-Rtn4-Stop transfected in HEK293 cells with an anti-Nogo A antibody (α-Nogo-A). Control, the empty vector; DMo-Rtn4-Stop, pCircRNA-DMo-Rtn4-Stop; DMo-Rtn4, pCircRNA-DMo-Rtn4. (**H**) Nucleotide sequence and predicted translation products surrounding the inserted sequences encoding the FLAG peptide (magenta) as part of exon 2, the region where exons 2 and 3 are joined, and the site of circularization for construct circRtn4-FLAG. Exon 2 nucleotide sequence is highlighted in blue, and black upper-case letters indicate Rtn4 exon 3. The nucleotides encoding the FLAG-tag are displayed in black lower-case letters. The last G residue (position 2448) is joined to the first nucleotide by circularization yielding a GAU triplet (highlighted in yellow), encoding aspartic acid (D, bold, blue letter, in brackets). Nucleotides are arranged in blocks of 12. The predicted amino acid sequence is given in the IUPAC one-letter amino acid code. Tryptic peptide 46 (Supplementary Fig. 6) beginning with TSDETL, bridging the circularization site and containing the FLAG peptide (bold and magenta lettering) is underlined. The glutamic acid residue bridging exons 2 and 3 is highlighted as bold blue letter. Unless additional start codon(s) are used, tryptic peptide 52 (Supplementary Fig. 6) starting with IMDLKEQPG (broken line) must be derived from translation into the second circular round across the presumed AUG start codon (marked by a horizontal green arrow). Amino acids are highlighted as above. (**I**) Mass spectrometry of peptide 46, providing direct evidence of circular translation of the junction site in circRtn4-FLAG. Amino acids are highlighted as above. (**J**) Mass spectrometry of peptide 52, providing evidence of circular translation into a second round of circRtn4-FLAG. Amino acids are highlighted as above. (**K**) Open reading frame (ORF) of circRtn4-FLAG-ac. Annotation as in B: In addition, the AC insertion is marked in red and the junction amino acid glutamine (Q, red) is marked. The different ORF beyond the first round of translation is marked in red and the extension of the first round in red and “69 aa”. The substitution to generate the stop codon (UAA) some 207 nucleotides further downstream is not shown in this C-terminal segment (Supplementary Fig. 2B). (**L**) Western blot analysis of pCircRNA-DMo-Rtn4-ac transfected in N2a cells using an anti-Nogo A antibody (α-Nogo-A). DMo-Rtn4-FLAG, pCircRNA-DMo-Rtn4-FLAG; DMo-Rtn4-FLAG-ac, pCircRNA-DMo-Rtn4-FLAG-ac.

The protein coding potential of circRNA was evaluated *via* Western blot with an anti-Nogo-A antibody for the detection of proteins related to circRtn4 expression. As potentially obstructing endogenous RTN4 protein expression is detectable but low in HEK293 cells, we resorted to this cell line for the analysis of circRtn4 translation. As a positive control, we utilized the pCMV-Rtn4 exon 2-exon 3 (Fig. 3A) vector, which yields the linear counterpart of the two circRtn4 exons (Supplementary Fig. 1). The corresponding protein product derived from pCMV-Rtn4 exon 2-exon 3 mRNA is predicted to yield an acidic protein (87.5 kDa, pI 4.3). Presumably, due to the high proportion of acidic amino acids and/or the high content of proline residues (57 out of 798 aa), the polypeptide atypically migrated much slower (~150 kDa) in the SDS-PAGE gel (Fig. 3C; Supplementary Fig. 1A,B)^39,40^. Analysis of pCircRNA-BE-Rtn4 vector transfection showed only traces, if any, of corresponding protein product. In contrast, when protein expression in pCircRNA-DMo-Rtn4 transfection experiments was examined, we detected specific signals for Rtn4-related proteins in HEK293 cells (Fig. 3B,C). In addition to the protein of expected size, we also uncovered signals representing Rtn4-related proteins of significantly higher molecular weight (Fig. 3B,C). Potentially, these proteins are products of continuous translation from circular templates (see below). The protein levels reflected the strong IME-dependent circRtn4 increase, presumably leading to enhanced circRNA translation.

### The “infinite” translation of circRtn4

We noticed that the circRtn4 open reading frame (ORF) lacks a stop codon in one of the reading frames, namely the one encoding the corresponding domain (exons 2 and 3) of the Rtn4 protein. In theory, the RNA contains a circular and consequently “infinite” ORF, which might give rise to proteins of very high molecular weight, representing products of “unterminated” polypeptide synthesis. In agreement, we observed high molecular weight signals by Western blot (under reducing conditions) for Rtn4-related proteins expressed in pCircRNA-DMo-Rtn4 transfected HEK293 cells (Fig. 3C). These large proteins presumably arise from the continuous translation of circRtn4 (Fig. 3B, Supplementary Fig. 1). To verify that these large proteins are a result of circRNA translation, we constructed the pCircRNA-DMo-Rtn4-FLAG plasmid by incorporation of a FLAG-tag into the 5′ portion of circRtn4 gene (circRtn4-FLAG in Fig. 3A,D). Since the FLAG-tag has been inserted upstream of the presumed start codon, the resulting protein only could be detectable in case of continuous, circular translation from the circRtn4-FLAG template and the presumed AUG start codon (Fig. 3A,D). Western blot with anti-FLAG-tag antibodies detected both the “monomer” (single round translation) and higher molecular weight products (two or more rounds of translation) (Fig. 3E). These results implied the continuous translation of the circular circRtn4 ORF (Fig. 3A,D,E,H; Supplementary Fig. 1C,D). To exclude that the larger polypeptides reflected protein aggregation of the “monomer” units, we inserted a stop codon into exon 2 (circRtn4-FLAG-Stop from pCircRNA-DMo-Rtn4-FLAG-Stop, Fig. 3A,F; Supplementary Fig. 2A). Western blot analysis with anti-Nogo-A antibody revealed that the stop codon entirely abolished the high molecular weight products, while the “monomeric” product of circRtn4-FLAG-Stop remained detectable (DMo-Rtn4-FLAG-Stop in Fig. 3G). In case high molecular weight proteins resulted from aggregation, the stop codon should not interfere with their formation. Thus, our data confirmed the expression of protein repeating units as a result of translation from the circular “infinite” ORF of circRtn4.

### Mass spectrometry analysis of circRtn4 translated peptides

For conclusive identification of proteins derived from circRtn4 translation, we performed mass spectrometry analysis for Rtn4 protein from pCircRNA-DMo-Rtn4 plasmid transfected into HEK293 cells (not antibody enriched). We detected 16 unique peptides (Supplementary Fig. 3), which covered about 37% of the “monomer” circRtn4 protein sequence (Supplementary Fig. 4A). Notably, the putative protein sequence of mouse circRtn4 is sufficiently different from the human counterpart (Supplementary Fig. 5). This ensured that peptides detected by mass spectrometry were translated from mouse circRtn4. For further analysis of continuous circRtn4 translation, we resorted to immunoprecipitation (IP) of circRtn4-FLAG derived protein (circRtn4-FLAG-DP) using the anti-FLAG antibody. Mass spectrometry (MS) of the immunoprecipitated proteins revealed peptides covering about 77% of the predicted sequences that were detected in our analysis (Supplementary Figs 4B and 6), underscoring the actual enrichment as a result of anti-FLAG immunoprecipitation.

Importantly, we identified two peptides from the circRtn4-FLAG construct (Fig. 3D,H,I,J), whose sequences supported translation from the circular RNA template. Peptide 46 (Fig. 3I, Supplementary Fig. 6) could have been generated neither from a linear template, nor from an endogenous human host cell RTN4 peptide, as it contains the FLAG sequence. The circularization involving the 3′ end of exon 3 (5′…,CUG,AAU,AAA,ACU,UCA,G 3′) and the 5′ end of exon 2 (5′ AU,GAG,ACC,CUU,UUU,GCU,GCA,… 3′) are responsible for the N-terminus of peptide 46 TSDETLFALPAASEPVIPDYKDDDDKPPSSAEK. The amino acid preceding the N-terminal threonine predicts a basic residue (K or R), due to the specificity of trypsin that has been used to generate peptides for mass spectrometry, which indeed is lysine near the 3′ end of exon 2 (Fig. 3I). The third amino acid of peptide 46 (Fig. 3I, Supplementary Fig. 6) was contributed by a composite codon precisely at the circularization site (between nucleotides 2448, 1, and 2 in Fig. 3H): it includes the 3′-most G nucleotide of exon 3 and the two 5′-most nucleotides of exon 2, namely AU yielding GAU, encoding aspartic acid (D). The fourth amino acid, glutamic acid (E), is the first complete triplet of exon 2 (Fig. 3H,I). The peptide also harbors the FLAG peptide sequence. In this context, it should be noted that trypsin is known to cleave incompletely or not at all, when Lys or Arg are followed by acidic residues (aspartic acid, D, or glutamic acid, E). Therefore, the peptide extends beyond the internal lysine that is followed by four aspartic acid residues. Importantly, the peptide 46 provided compelling evidence of circRnt4-FLAG translation, as such a junction peptide could only be translated from a circular template. Peptide 52 (Fig. 3J, Supplementary Fig. 6) is immediately adjacent (C-terminal) to peptide 46 and overlaps the fusion of exons 2 and 3 (Fig. 3D,K). Some evidence (i.e., length of the “monomeric” translation product, A/U-rich sequence preceding the AUG codon^41^) points to initiation at AUG (highlighted in green, position 96–98, Fig. 3H). If this is the case, peptide 52 includes the junction site for more than one round of circular polypeptide synthesis. Notably, the first amino acid of the tryptic peptide (isoleucine, I) must be preceded by a K or R, which is the case with lysine. The second amino acid is methionine (M). Even though peptide 52 harbours a lysine (K) at position 5, it is much longer. Once more, the basic residue is N-terminal to an acidic residue (glutamic acid residue, E) resulting in incomplete tryptic digestion (Fig. 3D,H; see also peptide 18, Supplementary Fig. 6,). We did not detect a peptide devoid of the isoleucine residue, such as MDLKEQPGN… (or DLKEQPGN…, if the methionine is post-translationally removed). Such a peptide would be expected, if the actual start occurred at the presumed AUG codon (green, position 96–98, Fig. 3H). Hence, we cannot rule out different translation start codons. Furthermore, due to the enrichment with antibody in case of FLAG-tag, we would not have been able to detect additional products translated from the two other reading frames, although they would be significantly smaller, at most about 6.8 kDa (see Supplementary Fig. 1D)

Finally, we incorporated two additional nucleotides (AC) after the FLAG-tag in the pCircRNA-DMo-Rtn4-FLAG construct (Fig. 3A,K, Supplementary Fig. 2B) to generate pCircRNA-DMo-Rtn4-FLAG-ac vector. The insertion of the dinucleotide sequence into circRtn4-FLAG resulted in a reading frame shift, yielding (after additionally converting an encountered stop codon to a triplet encoding glutamine, Q) a novel ORF with an additional 69 amino acids at the presumed circRtn4-FLAG-DP “monomeric” C-terminus (Fig. 3A,K,L). This new ORF encounters a *bona fide* stop codon (UAA) further downstream, thus continued translation would be abolished shortly after the first round (see Supplementary Fig. 2B). Indeed, Western blot analysis of pCircRNA-DMo-Rtn4-FLAG-ac transfections in N2a cells using NogoA antibody once more revealed signals only for the expression of the Rtn4 “monomer” (Fig. 3A,K,L).

Furthermore, IVS1 and PAT1 introns increased the formation of circRtn4 and its translated protein in N2a cells as well (Supplementary Fig. 7). Based on several lines of evidence, we concluded that IME is capable of significantly increasing translation of circRtn4, indicating that other circRNAs could also be efficiently translated in mammalian cells. Moreover, our data clearly revealed that protein synthesis from a circular “infinite” ORF produces what appears to be “monomer” and “multimer” repeating polypeptides in living cells.

### Employing pCircRNA-DMo vector to generate other circRNAs

**Figure 4:**
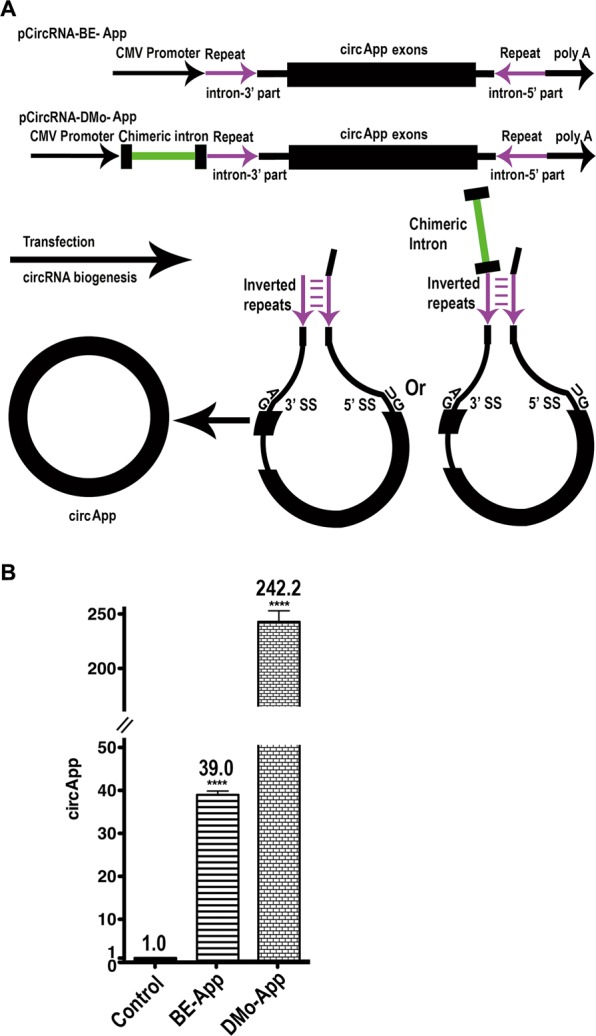
IME promotes circApp generation. (**A**) Schematic representation for the mouse circApp cassette of pCircRNA-BE-App and pCircRNA-DMo-App constructs. (**B**) CircApp formation in N2a cells after transfection with circApp constructs. Control, empty vector; BE-App, pCircRNA-BE-App; DMo-App, pCircRNA-DMo-App; all T-tests were performed in comparison to levels of the control sample ****P ≤ *0*.*0001*, *n* ≥ 4.

Here we demonstrated that insertion of IME significantly elevated circRtn4 formation and translation, offering a useful tool for the analysis of circRNA functions. We recently used the pCircRNA-DMo vector to express additional circRNAs. For example, the cDNA of a part of mouse amyloid precursor protein (App) corresponding to circular RNA mmu_circ_0000705^42^ was inserted into pCircRNA-BE and pCircRNA-DMo vectors (Fig. 4A). Transfection of these vectors in N2a cells demonstrated that pCircRNA-DMo generated circApp at levels about 6.2 times higher than using the pCircRNA-BE plasmid (Fig. 4B).

In addition, we demonstrated that the processes during circApp formation employing these vectors underwent splicing events leading to the same mature RNA sequence as wild type circApp from mouse brain (data not shown). These results were corroborated with other human circRNA, which again confirmed that IME enhanced circRNA formation and translation (Dingding Mo *et al*., unpublished data).

This demonstrated that the boosting effect of IME on circRNA is not limited to circRtn4. The approaches described are a generally applicable to enhance circRNA formation and translation for investigation of circRNA functions including their poorly understood translational capacity.

## Discussion

Perhaps the most intriguing question regarding circRNAs centers around their potential functions. Several interesting hypotheses have been put forward, yet clear functions for most circRNAs remain elusive^19,43^. CircRNAs are chiefly derived from protein coding genes and are produced by backsplicing of hnRNA in parallel to processing into linear canonical mRNA. Since these RNA species are mostly located within the cytoplasm and some of them co-sediment with ribosomes, an appealing idea is that circRNAs also serve as templates for protein synthesis^19,37,44,45^. Previous reports demonstrated that several *in vitro* synthesized circular RNAs are translated *in vitro* and *ex vivo*^46,47^. For example, an artificially split GFP (green fluorescence protein) circRNA can produce intact GFP protein^48^. Furthermore, it has been reported that endogenous circ-ZNF609 is translated into protein within cell lines, especially under stress conditions^49^. A circRNA generated from the muscleblind locus serves as a template for a protein in *Drosophila* brain^50^ and N6-methyladenosine (m6A) modification of circRNA causes efficient initiation of circRNA translation^51^. A circRNA from β-catenin can produce protein and promote liver cancer cell growth^52^. The oncoprotein encoded by circE7 from human papillomavirus (HPV) is weakly expressed, yet has transforming activity^53^.

Previous studies of circRNA protein coding functions were severely hampered by the lack of efficient molecular tools to specifically increase their expression levels^19^. In 2013, Hansen *et al*. were the first to report the use of an artificially inverted intronic sequence to promote circRNA biogenesis^13^ and subsequently several other groups reported circRNA vectors based on a similar strategy^20,21,23,48^. These constructs provided valuable insights into circRNA formation and were a starting point for the design of the circRNA vectors presented here. Efficient protein production under physiological conditions from these earlier circRNA vectors remains underreported^54^ (Table 2). For example, the protein product from circ-ZNF609 only amounted to 0.1% compared to the product translated from the linear mRNA in HeLa cells when it was expressed in the circRNA mini vector ZKSCAN1 (Table 2) (Figure S6B in Kramer *et al*., 2015)^49,55^. Moreover, Stagsted *et al*. (2019) examined protein expression from circRNAs harboring exons that contain start codons annotated on linear mRNAs (so-called AUG cirRNAs), but failed to detect any translation products (Table 2)^54^. This could be due to the method itself^54^ or the fact that start codons selected for their function in *bona fide* linear mRNAs do not function in the context of circular RNAs. Similarly, we initially constructed pCircRNA-BE-Rtn4 with the method by Hansen *et al*., leading to considerable amounts of circRtn4 RNA detectable by RT-PCR and Northern blots. However, the vector failed to sufficiently sustain Rtn4-related protein synthesis in cell lines (Table 2). Here we demonstrated that intron-mediated enhancement (IME) is a suitable tool to boost circRtn4 formation leading to significant levels of translation, compared to very low or even lack of protein synthesis with the previously published vector systems (Table 2)^36^. In human HEK293 cells, circRtn4 generated with the pCircRNA-DMo vector amounted to at least 25% of protein product compared to its linear mRNA counterpart. Thus, for the purpose of protein production, our circRNA vector showed significant improvement (roughly 250-fold increase compared to the circRNA mini vector ZKSCAN1^21,49^. Further studies are necessary to establish whether one (or a few) internal start codons are responsible for the significant levels of translation in our system and what are the sequence and/or structural environments in the circRNA that are capable of supporting efficient translation initiation or whether trans translation factors/elements are involved (see below). The versatility of this method was further demonstrated by the use of two other introns that also boosted circRNA formation. Analysis of different circRNAs showed that IME-dependent induction is a general effect, underscoring its general utility. Interestingly, since the majority of abundant circRNAs are derived from central exon(s) of host genes^20^, their flanking introns may contribute IME-like effects that promote circRNA formation and translation.

**Table 2:** Comparison of protein levels from various circRNA vectors.

circRNA expressed by different vectors	circRNA formation	co-expression of linear RNA shown in Northern blot	circRNA encoded protein detected by Western blot	ratio to the protein produced by linear mRNA	protein detected by mass spectrometry	References
circLPAR1, circSLC8A1, circCDYL generated by Hansen’s method	yes	yes	no	not applicable	not applicable	^13, 54^
circRtn4 generated by pCircRNA-BE	yes	not detected	no	not applicable	not applicable	this study^36^
circRtn4 expressed by pCircRNA-DMo	yes improved	not detected	yes, strong	**25%**	yes; **76**.**8%** coverage for IP-MS with anti-FLAG antibody	this study^36^
circ-ZNF609 expressed by circRNA mini vector ZKSCAN1	yes	yes	yes, but weak	**0**.**1%**	yes; **little** coverage for IP-MS with anti-FLAG antibody	^21, 49^
circMbl expressed by vector with Mbl minigenes	yes	yes	yes	not determined	not determined	^50^
circE7 expressed by vector with quaking (QKI) protein-binding sites	yes	not determined	yes	not determined	not determined	^12, 53^

Published circRNAs with protein coding capacity are presented. Not determined: the experiment was not performed or provided in the reference. No data for linear mRNA expression was provided for circMbl and circE7, therefore the rate of protein synthesis from vector-derived circRNA could not be compared. A single peptide mass spectrum from the endogenous circMbl of *Drosophila* brain was obtained, but no mass spectrometry from protein of the vector-derived circRNA was performed.

Our IME based improvement of circRNA expression can help accelerate functional studies including generation of truncated protein variants. Although it is possible that the majority of circRNAs are mere by-products and represent nothing but noise from low frequency backsplicing events, recent findings indicate that a significant number of circRNAs are translatable yielding additional protein variants^43,56^.

Here we demonstrated that circRtn4 is efficiently translated into what appear to be “monomer” polypeptides as well as larger “multimeric” polypeptides, in case the size of the circular RNA is divisible by three and a stop codon is not encountered beyond the translational start site. Several rounds of translation had previously been observed with artificial circRNAs^46,47^. Here, we confirmed this mechanism with natural circRNAs. Our finding suggests that other circRNAs may also contain “infinite” ORFs, thus adding new dimensions to RNA translation and protein diversity^57^. Even with circles not divisible by 3, variant C-termini could be generated by circRNA translation if a single round of translation does not encounter a translation stop codon but will translate out of frame at the beginning of the second round and then encounters a stop codon further downstream. This would correspondingly extend the “monomer” at the C-terminus with a very different polypeptide portion and, apart from alternative spicing, further increase protein diversity out of a single gene. This scenario could be mirrored in our circRtn4-FLAG-ac construct, where translation out of frame would add about 69 amino acids to the “first-round” polypeptide (Supplementary Fig. 2B). However, no MS data were collected from this particular construct. Interestingly, from our Western blots (Fig. 3C,E; Supplementary Fig. 7) it appears that the theoretical “infinite” translation in circRtn4 and circRtn4-FLAG produces significant amounts of a polypeptide that stops after a single round. Larger bands, presumably corresponding to dimers and trimers were present at varying levels depending on the construct (Fig. 3; Supplementary Fig. 7); this differs from *in vitro* studies, where an excess of products of multiple rounds of translation are apparent^46^. Expectedly, our constructs with stop codons only produced a single band (circRtn4-FLAG-Stop and circRtn4-FLAG-Stop-ac; Fig. 3F,G,K,L).

Unresolved remains the exact locus of translation initiation in our constructs. In theory, any of the 16 AUG codons that could initiate a full round (or more) of translation on the circular template (i.e., not encountering a stop codon) could yield a polypeptide, at least covering one circle of translation (Supplementary Fig. 1A). However, in the Western blot, construct circRtn4-FLAG-Stop did not exhibit shorter polypeptides (Fig. 3F,G), unless they all would be undetectable with the α-Nogo-A antibody employed. Similarly, shorter peptides derived from other reading frames (Supplementary Fig. 1B) would not be detected in Western blots. Likewise, due to the polypeptide enrichment step via immunoprecipitation with FLAG antibody, the corresponding peptides would not be detected in mass spectrometry. On the RNA sequence, we could not detect any sequences resembling internal ribosome entry sites (IRES). It has been suggested that A/U-rich regions surrounding AUG start codons, would support translation initiation^41^. This is the case for the AUG codon at position 66–68 (Supplementary Fig. 1A) corresponding to position 96–98 in Fig. 3H. The “monomer” bands generated from circRtn4-FLAG-Stop and circRtn4-FLAG-Stop-ac, both harbouring in frame stop codons (Fig. 3F,G,K,L), strongly point to the aforementioned AUG (or one in its vicinity further downstream) as the major or even only start codon. With the current vector system, ensuring reasonable levels of circRNA translation, we should be able to solve these questions by delineating the sequences/structures favouring translation initiation. For example, are there specific sequences in circRNAs that serve as the IRES-like elements for cap-independent translation initiation in the absence of 5′ cap and 3′ poly(A) elements recognized by the translation machinery^41^? Also, the question whether a coupling mechanism of circRNA backsplicing might interact with translation initiation in IME remains to be investigated.

Even more mysterious was the aforementioned observation that significant levels of translation appear to terminate after the first or further rounds of circular translation as the polypeptide(s) derived from the circRtn4 ORF are restricted in size (Fig. 3B,C). This indicated that there would be a hitherto unknown mechanism of translational termination for at least some “infinite” ORFs of circular RNAs. Once more, our vector system should make it possible to shed light on this, thus far, unexplainable observation. Hence, mechanism of translational initiation and termination for the corresponding “monomeric” polypeptides have to be explored and analysed in broader detail.

In conclusion, IME provides convincing benefits for circRNA formation and translation, thus serving as an excellent tool to investigate various circRNA coding functions and modes of translation.

## Materials and Methods

### Plasmid construction

#### Generation of pCircRNA-BE-Rtn4

For construction of circRtn4 plasmid, the genomic region containing circRtn4 exons (chr11: 29, 704, 497–29, 708, 881, mouse GRCm38/mm10) including partial 5′ and 3′ flanking intronic sequences (1014 bp and 111 bp) were amplified by PCR from genomic DNA templates isolated from mouse N2a cell using oligonucleotides listed in Supplementary Table 1. The product was inserted into pCMV-MIR (OriGene) containing the CMV promoter for transcription. The resulting construct is referred to as control-2 (Fig. 1A). Inverted repeats enhance efficiency of backsplicing and the formation of circRNAs^13,20^. For this purpose, a region of 800 nucleotides representing 5′ intronic portions (corresponding to chr11: 29, 704, 521-29, 705, 320) of control-2 was selected^13^. This region was incorporated into the 3′ flanking intron to generate the downstream portion of the inverted repeat. Therefore, its relative orientation in the resulting cassette was inverted with respect to its 5′ intronic counterpart^4,13^. As the flanking introns lack both 5′ and 3′ splice sites, they were incapable of supporting the canonical splicing reaction and to generate linear mRNAs. The resulting construct was designated as pCircRNA-BE-Rtn4.

#### Generation of pCircRNA-DMo-Rtn4

pCircRNA-DMo-Rtn4 was constructed from pCircRNA-BE-Rtn4 vector with the insertion of a chimeric intron, derived from pCI-neo-FLAG, upstream from the circRNA domain (oligonucleotides employed are given in Supplementary Table 1). The mouse *Rtn4*-derived gene components and their hypothetical translation products are provided in Supplementary Fig. 1A–D.

#### Generation of pCircRNA-BE and pCircRNA-DMo

For the construction of general vectors for circRNA expression, multiple restriction endonuclease sites (*Bgl*II, *Nhe*I, *Bmt*I, *Eco*RV, *Not*I, *Sac*II, and *Xba*I) were inserted in place of the original circRtn4 exon of pCircRNA-BE-Rtn4 or pCircRNA-DMo-Rtn4, leading to vectors pCircRNA-BE or pCircRNA-DMo for general use (Supplementary Fig. 8) (oligonucleotides employed are given in Supplementary Table 1).

#### Generation of pCircRNA-IVS1-Rtn4 and pCircRNA-PAT1-Rtn4

The chimeric intron of pCircRNA-DMo-Rtn4 was replaced by the IVS1 intron or PAT1 intron 1 to construct pCircRNA-IVS1-Rtn4 and pCircRNA-PAT1-Rtn4 plasmids^26,32^ (oligonucleotides employed are given in Supplementary Table 1).

#### Generation of pCMV-Rtn4-Exon2-Exon3

Linear mRNA generation of Rtn4 exon 2-exon 3 was achieved by inserting mouse Rtn4 exon 2/intron2/exon 3 into the pCMV-MIR vector (OriGene); resulting in construct pCMV-Rtn4-Exon2-Exon3 (oligonucleotides employed are given in Supplementary Table 1).

#### Generation of pCircRNA-DMo-Rtn4-FLAG, pCircRNA-DMo-Rtn4-FLAG-Stop and pCircRNA-DMo-Rtn4-FLAG-ac

A sequence encoding the FLAG-tag (DYKDDDDKPP) was inserted into exon 2 of pCircRNA-DMo-Rtn4 (47 nt after the junction site of the circle), resulting in vector pCircRNA-DMo-Rtn4-FLAG (Fig. 3H). An additional triplet (TGA) inserted 3′ to the FLAG encoding sequence yielded pCircRNA-DMo-Rtn4-FLAG-Stop (Supplementary Fig. 2A). pCircRNA-DMo-Rtn4-FLAG-ac was constructed by addition of an AC dinucleotide 3′ to the FLAG-tag of pCircRNA-DMo-Rtn4-FLAG (oligonucleotides employed are given in Supplementary Table 1). In order to allow translation for 69 additional codons, an in-frame stop codon TAG was changed into CAG (glutamine, Q) by a point mutation at position 107 (Supplementary Fig. 2B).

#### Generation of pCircRNA-BE-App and pCircRNA-DMo-App

The cDNA of mouse CircApp (mmu_circ_0000705^42^) was inserted into pCircRNA-BE and pCircRNA-DMo vectors to construct plasmids pCircRNA-BE-App and pCircRNA-DMo-App (oligonucleotides employed are given in Supplementary Table 1).

### Plasmid preparation

Plasmid DNA was purified with an EndoFree Plasmid Maxi Kit (QIAGEN). To ensure the integrity of the resulting circRNA vectors, all constructs were verified by double restriction endonuclease digestion and DNA sequencing (data not shown).

### Cell culture and plasmid DNA transfection

Cell lines N2a, HeLa, HEK293 were cultured in Dulbecco’s modified Eagle medium (DMEM, Invitrogen), supplemented with 10% fetal bovine serum (Gibco), 10 mM sodium pyruvate (Sigma), 100 U/ml penicillin and 100 U/ml streptomycin (Gibco) at 37 °C in 5% (v/v) CO_2_. N2a-swe.10 cells were cultured in a specialized medium as previously described^58^. For transient transfection, 2.5 μg of plasmid DNA diluted in 150 μl Opti-MEM (Invitrogen) were mixed with 5 μl lipofectamine 2000 diluted in 150 μl Opti-MEM; the resulting transfection mix was added to approximately half million cells in 6 well plates. After 24 hours, the transfection mix was replaced by fresh DMEM medium. Three days after transfection, cells were harvested for total RNA and protein extraction.

GFP (green fluorescence protein), which was expressed from the SV40 promoter within the pCMV-MIR backbone, was utilized as a reporter gene for monitoring transfection efficiency; GFP expression was measured by Western blot with an antibody directed against GFP (#2555, Cell signalling Technology). Almost equal GFP expression levels were observed for independent experiments, indicating comparable transfection efficiencies for assays with different circRNA plasmids (Supplementary Fig. 9).

### Total RNA isolation and qRT-PCR

Total RNAs from N2a, N2a-swe.10, HeLa and HEK293 cells were isolated using the TRIzol reagent (Ambion) according to the manufacturer’s recommendations. For cDNA synthesis, 0.5 µg of total RNA served as template for reverse transcription with the SuperScript®III First-Strand Synthesis System (Invitrogen) and random hexamers for priming. Quantitative PCR amplification was performed with the 7900HT Fast Real Time PCR System (Applied Biosystems) using the Power SYBR Green PCR Master Mix (Applied Biosystems). Fold differences between treated samples versus control samples were calculated using the 2^−ΔΔ*C*^_T_ method with β-Actin mRNA as internal control^59^.

### Northern blots

Northern blots were performed with NorthernMax Kit (AM1940, Ambion) as previously described with minor changes^38^. In brief, 15 µg of total RNA of transfected HEK293 were separated by 1% agarose gel electrophoresis and transferred to positively charged nylon membranes (Amersham Hybond-N^+^, GE Healthcare). DNA oligonucleotide probe Rtn4-NB-R1 was 5′ end-labelled with P^32^ and incubated with the blot membrane at 42 °C and washed according to the provided protocol in the kit.

Rtn4-NBR1: 5′ TCCTGAACTAAATCTGGCGTTAGACCTTCAGGCATGGTTGCCACTACTGCCTCAGTCACC 3′

For RNase R treatment, 15 µg of total RNAs were digested with 10 units of RNase R (RNR07250, Epicentre) for 1 hour at 37 °C.

### Western blots

Total protein extracts from about 5 million HEK293 or N2a cells were prepared in RIPA buffer (50 mM Tris-HCl pH 8.0, 150 mM NaCl, 0.1% (w/v) SDS, 0.5% (w/v) Na-Deoxycholate, 1% (v/v) NP40, 1 x Roche cOmplete Protease Inhibitor and 1 x PhosSTOP Phosphatase Inhibitor). Total protein (40 μg) was fractioned on gradient gels (Any kD™ Criterion™ TGX Stain-Free™ Protein Gel, #5678124, Bio-Rad) with reducing loading buffer (5% β-mercaptoethanol) and transferred to nitrocellulose membranes (10600002, Amersham). The following antibodies Nogo-A (#13401, Cell signalling Technology), FLAG (F3165, Sigma) and β-actin (A5441, Sigma) were used for immunoblotting in 5% milk-TBST buffer. The filters were washed with TBST buffer and incubated with horseradish peroxidase conjugated secondary antibodies (goat-anti-rabbit, IgG (H + L), G21234; goat-anti-mouse IgG (H + L), G21040; Life technologies) and signals were developed with ECL solution (RPN2109, Amersham) and visualized with the ChemiDoc MP Imaging System (Bio-Rad).

### LC-MSMS analysis

HEK293 cells, which were transfected with pCircRNA-DMo-Rtn4 and empty plasmids for control were lysed and digested as previously described^60^. Briefly, cell pellets were heated and sonicated in lysis buffer (100 mM Tris-HCl, 6 M guanidine hydrochloride [GuHCl], 10 mM TCEP [Tris (2-carboxyethyl) phosphine], 40 mM CAA [chloroacetamide]). After centrifugation, diluted supernatants were digested by trypsin (V5280, Promega) overnight; the resulting peptides were purified with C18-SD StageTip^60,61^. This peptide preparation was analysed by the Orbitrap Fusion mass spectrometer (Thermo Fisher Scientific) with a nano-electrospray ion source, coupled to the EASY-nLC 1000 (Thermo Fisher Scientific) UHPLC system for separation. MaxQuant version 1.5.3.8 with an integrated Andromeda search engine was utilized for analysis of LC-MS/MS raw data^61,62^. The detailed method is provided in Supplementary Materials and Methods.

### Immunoprecipitation and mass spectrometry

Five million of N2a cells transfected with the pCircRNA-DMo-Rtn4-FLAG plasmid were collected, lysed and bound to the ANTI-FLAG-M2 affinity gel (FLAG Immunoprecipitation Kit, Sigma-Aldrich, FLAGIPT1) according to the manufacture’s recommendations. After washing with PBS buffer, the elution buffer containing 5 ng/µl trypsin, 50 mM Tris-HCL, TCEP (Tris(2-carboxyethyl) phosphine), 5 mM chloroacetamide pH 7.5, was added to the resin and incubated for 30 minutes at room temperature with gentle agitation. The resulting supernatants were transferred to fresh tubes and incubated at 37 °C overnight to ensure a complete tryptic digestion. All other procedures were performed as previously described in the section of “LC-MSMS analysis”.

## Supplementary information

Supplementary information
