# Supplementary material for: A universal approach to investigate circRNA protein coding function
Source: Sci Rep. 2019 Aug 12;9:11684. doi: 10.1038/s41598-019-48224-y (PMC6690939; doi:10.1038/s41598-019-48224-y)
Supplement: Supplementary file 1 — Supplementary information [file 41598_2019_48224_MOESM1_ESM.pdf]

Supplementary information

A universal approach to investigate circRNA protein coding function

Running title: Intron-mediated enhancement boosts circRNA translation

Dingding Mo[1, 2, *], Xinping Li[1], Carsten A. Raabe[3, 4, 5], Di Cui[1], Jeanne-Franca Vollmar[1], Timofey S. Rozhdestvensky[2], Boris V. Skryabin[2], Juergen Brosius[3, 6]

1. Max Planck Institute for Biology of Ageing, Joseph-Stelzmann-Strasse 9b, 50931 Cologne, Germany.
2. Medical Faculty, Core Facility Transgenic Animal and Genetic Engineering Models (TRAM), University of Münster, Von-Esmarch-Str. 56, D-48149 Münster, Germany.
3. Institute of Experimental Pathology, Centre for Molecular Biology of Inflammation (ZMBE), University of Münster, Von-Esmarch-Str. 56, D-48149 Münster, Germany.
4. Brandenburg Medical School (MHB), Fehrbelliner Strasse 38, D-16816 Neuruppin, Germany.
5. Institute of Medical Biochemistry, Centre for Molecular Biology of Inflammation (ZMBE), University of Münster, Von-Esmarch-Strasse 56, D-48149 Münster, Germany.
6. Institutes for Systems Genetics, West China Hospital, Sichuan University, Chengdu 610041, China.

*. To whom correspondence should be addressed. E-mail: modingding@163.com

Supplementary Fig. 1

A.

```
MEDIDQSSLV SSSADSPPRP PPAFKYQFVT EPEDEEDEED EEEEEDDEDL
        60         70         80         90        100
EELEVLERKP AAGLSAAPVP PAAAPLLDFS SDSVPPAPRG PLPAAPPTAP
       110        120        130        140        150
ERQPSWERSP AASAPSLPPA AAVLPSKLPE DDEPPARPPA PAGASPLAEP
       160        170        180        190        200
AAPPSTPAAP KRRGSGSVDE TLFALPAASE PVIPSSAEKI MDLKEQPGNT
       210        220        230        240        250
VSSGQEDFPS VLFETAASLP SLSPLSTVSF KEHGYLGNLS AVASTEGTIE
       260        270        280        290        300
ETLNEASREL PERATNPFVN RESAEFSVLE YSEMGSSFNG SPKGESAMLV
       310        320        330        340        350
ENTKEEVIVR SKDKEDLVCS AALHNPQESP ATLTKVVKED GVMSPEKTMD
       360        370        380        390        400
IFNEMKMSVV APVREEYADF KPFEQAWEVK DTYEGSRDVL AARANMESKV
       410        420        430        440        450
DKKCFEDSLE QKGHGKDSES RNENASFPRT PELVKDGSRA YITCDSFSSA
       460        470        480        490        500
TESTAANIFP VLEDHTSENK TDEKKIEERK AQIITEKTSP KTSNPFLVAI
       510        520        530        540        550
HDSEADYVTT DNLSKVTEAV VATMPEGLTP DLVQEACESE LNEATGTKIA
       560        570        580        590        600
YETKVDLVQT SEAIQESIYP TAQLCPSFEE AEATPSPVLP DIVMEAPLNS
       610        620        630        640        650
LLPSTGASVA QPSASPLEVP SPVSYDGIKL EPENPPPYEE AMSVALKTSD
       660        670        680        690        700
SKEEIKEPES FNAAAQEAEA PYISIACDLI KETKLSTEPS PEFSNYSEIA
       710        720        730        740        750
KFEKSVPDHC ELVDDSSPES EPVDLFSDDS IPEVPQTQEE AVMLMKESLT
       760        770        780        790        800
EVSETVTQHK HKERLSASPQ EVGKPYLESF QPNLHITKDA ASNEIPTLTK
       810        820        830        840        850
KETISLQMEE FNTAIYSNDD LLSSKEDKMK ESETFSDSSP IEIIDEFPTF
       860        870        880        890        900
VSAKDDSPKE YTDLEVSNKS EIANVQSGAN SLPCSELPCD LSFKNTYPKD
       910        920        930        940        950
EAHVSDEFSK SRSSVSKVPL LLPNVSALES QIEMGNIVKP KVLTKEAEEK
       960        970        980        990       1000
LPSDTEKEDR SLTAVLSAEL NKTSVVDLLY WRDIKKTGVV FGASLFLLLS
      1010       1020       1030       1040       1050
LTVFSIVSVT AYIALALLSV TISFRIYKGV IQAIQKSDEG HPFRAYLESE
      1060       1070       1080       1090       1100
VAISEELVQK YSNSALGHVN STIKELRRLF LVDDLVDSLK FAVLMWVFTY
      1110       1120       1130       1140       1150
VGALFNGLTL LILALISLFS IPVIYERHQA QIDHYLGLAN KSVKDAMAKI
      1160
QAKIPGLKRK AE
```

A. Full protein sequence of mouse Rtn4.

The contributions of exon 2 and 3 are highlighted in yellow and green, respectively. The glutamic acid codon (represented by E at position 188 of the protein sequence) is contributed by both exons, while the aspartic acid residue D at position 169 (highlighted in blue) resulted from circularization and remains D in the product of circular translation.

B.

```
MDLKEQPGNT VSSGQEDFPS VLFETAASLP SLSPLSTVSF KEHGYLGNLS
        10         20         30         40         50
AVASTEGTIE ETLNEASREL PERATNPFVN RESAEFSVLE YSEMGSSFNG
        60        270         80         90        100
SPKGESAMLV ENTKEEVIVR SKDKEDLVCS AALHNPQESP ATLTKVVKED
       110        120        130        140        150
GVMSPEKTMD IFNEMKMSVV APVREEYADF KPFEQAWEVK DTYEGSRDVL
       160        170        180        190        200
AARANMESKV DKKCFEDSLE QKGHGKDSES RNENASFPRT PELVKDGSRA
       210        220        230        240        250
YITCDSFSSA TESTAANIFP VLEDHTSENK TDEKKIEERK AQIITEKTSP
       260        270        280        290        300
KTSNPFLVAI HDSEADYVTT DNLSKVTEAV VATMPEGLTP DLVQEACESE
       310        320        330        340        350
LNEATGTKIA YETKVDLVQT SEAIQESIYP TAQLCPSFEE AEATPSPVLP
       360        370        380        390        400
DIVMEAPLNS LLPSTGASVA QPSASPLEVP SPVSYDGIKL EPENPPPYEE
       410        420        430        440        450
AMSVALKTSD SKEEIKEPES FNAAAQEAEA PYISIACDLI KETKLSTEPS
       460        470        480        490        500
PEFSNYSEIA KFEKSVPDHC ELVDDSSPES EPVDLFSDDS IPEVPQTQEE
       510        520        530        540        550
AVMLMKESLT EVSETVTQHK HKERLSASPQ EVGKPYLESF QPNLHITKDA
       560        570        580        590        600
ASNEIPTLTK KETISLQMEE FNTAIYSNDD LLSSKEDKMK ESETFSDSSP
       610        620        630        640        650
IEIIDEFPTF VSAKDDSPKE YTDLEVSNKS EIANVQSGAN SLPCSELPCD
       660        670        680        690        700
LSFKNTYPKD EAHVSDEFSK SRSSVSKVPL LLPNVSALES QIEMGNIVKP
       710        720        730        740        750
KVLTKEAEEK LPSDTEKEDR SLTAVLSAEL NKTSARAETH LRRGSGSK
       760        770        780        790      798
```

B. Protein sequence of linear counterpart mRNA expressed by pCMV-Rtn4-Exon2-Exon3. The contributions of exon 3 are highlighted in green. The amino acid sequence from the vector is in gray.

C.

```
13
AUGAGACCCUUU UUGCUCUUCCUG CUGCAUCUGAGC CUGUGAUACCCU CCUCUGCAGAAA   60
  E   T   L   F    A   L   P   A    A   S   E   P    V   I   P   S    S   A   E   K

AAAUUAUGGAUU UGAAGGAGCAGC CAGGUAACACUG UUUCGUCUGGUC AAGAGGAUUUCC  120
  I   M   D   L    K   E   Q   P    G   N   T   V    S   S   G   Q    E   D   F   P...

CAUCUGUCCUGU UUGAAACUGCUG CCUCUCUUCCUU CUCUAUCUCCUC UCUCAACUGUUU  180

CUUUUAAAGAAC ACGGAUACCUUG GUAACUUAUCAG CAGUGGCAUCCA CAGAAGGAACUA  240
                  15
UUGAAGAAACUU UAAAUGAAGCUU CUAGAGAAUUGC CAGAGAGGGCAA CAAAUCCAUUUG  300

UAAAUAGAGAGU CAGCAGAGUUUU CAGUAUUAGAAU ACUCAGAAAUGG GAUCAUCUUUCA
10
AUGGCUCCCCAA AAGGAGAGUCAG CCAUGUUAGUAG AAAACACUAAGG AAGAAGUAAUUG

UGAGGAGUAAAG ACAAAGAGGAUU UAGUUUGUAGUG CAGCCCUUCAUA AUCCACAAGAGU
```

CACCUGCGACCC UUACUAAAGUGG UUAAAGAAGACG GAGUUAUGUCUC CAGAAAAGACAA

2 11

UGGACAUUUUUA AUGAAAUGAAAA UGUCAGUGGUAG CACCUGUGAGGG AAGAGUAUGCAG 600

6 51 46

AUUUUAAGCCAU UUGAACAAGCAU GGGAAGUGAAAG AUACUUAUGAGG GAAGUAGGGAUG

2

UGCUGGCUGCUA GAGCUAAUAUGG AAAGUAAAGUGG ACAAAAAAUGCU UUGAAGAUAGCC

20 12 10

UGGAGCAAAAAa GUCAUGGGAAGG AUAGUGAAAGCA GAAAUGAGAAUG CUUCUUUCCCCA

GUACCCCAGAAC UUGUGAAGGACG GCUCCAGAGCGU ACAUCACCUGUG AUUCCUUUAcCU

CAGCAACCGAGA GUACUGCAGCAA ACAUUUUCCCUG UGCUAGAAGAUC ACACUUCAGAAA 900

4

AUAAAACAGAUG AAAAAAAAAUAG AAGAAAGGAAGG CCCAAAUUAUAA CAGAGAAGACUA

14 9

GCCCCAAAACGU CAAAUCCUUUCC UUGUAGCAAUAC AUGAUUCcGAGG CAGAUUAUGUCA

CAACAGAUAAUU UAUCAAAGGUGA CUGAGGCAGUAG UGGCAACCAUGC CUGAAGGUCUAA

14

CGCCAGAUUUAG UUCAGGAAGCAU GUGAAAGUGAAC UGAACGAAGCCA CAGGUACAAAGA

46

UUGCUUAUGAAA CAAAAGUGGACU UGGUCCAGACAU CAGAAGCUAUAC AAGAGUCAAUUU 1200

ACCCCACAGCAC AGCUUUGCCCAU CAUUUGAGGAAG CUGAAGCAACUC CGUCACCAGUUU

UGCCUGAUAUUG UUAUGGAAGCUC CAUUAAAUUCUC UCCUUCCAAGCA CUGGUGCUUCUG

3

UAGCGCAGCCCA GUGCAUCCCCAC UAGAAGUACCGU CUCCAGUUAGUU AUGACGGUAUAA

AGCUUGAGCCUG AAAAUCCCCCAC CAUAUGAAGAAG CCAUGAGUGUAG CACUAAAAACAU

17

CGGACgCAAAGG AAGAAAUUAAAG AGCCUGAAAGUU UUAAUGCAGCUG UUCAGGAAGCAG 1500

1

AAGCUCCUUAUA UAUCCAUUGCAU GUGAUUUAAUUA AAGAAACAAAGC UCUCCACUGAGC

CAAGUCCAGAGU UCUCUAAUUAUU CAGAAAUAGCAA AAUUUGAGAAGU CGGUGCCUGAUC

28 14

ACUGUGAGCUCG UGGAUGAUUCCU CACCgGAAUCUG AACCAGUUGACU UAUUUAGUGAUG

AUUCAAUUCCUG AcGUCCCACAAA CACAAGAGGAGG CUGUGAUGCUAA UGAAGGAGAGUC

UCACUGAAGUGU CUGAGACAGUAA CACAACACAAAC AUAAGGAGAGAC UUAGUGCUUCAC 1800

CUCAGGAGGUAG GAAAGCCAUAUU UAGAGUCUUUUC AGCCCAAUUUAC AUAUUACAAAAG

8 5

AUGCUGCAUCUA AUGAAAUUCCAA CAUUGACCAAAA AGGAGACAAUUU CUUUGCAAAUGG

11

AAGAGUUUAAUA CUGCAAUUUAUU CCAAUGAUGACU UACUUUCUUCUA AGGAAGACAAAA

19

```
UGAAAGAAAGUG AAACAUUUUCCG AUUCAUCUCCCA UUGAGAUAAUAG AUGAGUUUCCCA
                    9
CAUUUGUCAGUG CUAAAGAUGAUU CUCCUAAGGAGU ACACUGACCUAG AAGUAUCCAACA 2100
            58
AAAGUGAAAUUG CUAAUGUCCAGA GCGGGGCCAAUU CGUUGCCUUGCU CAGAAUUGCCCU
                                      32        29        26
GUGACCUUUCUU UCAAGAAUACAU AUCCUAAAGAUG AAGCACAUGUCU CAGAUGAAUUCU
                                                  8
CCAAAAGUAGGU CCAGUGUAUCUA AGGUGCCCUUAU UGCUUCCAAAUG UUUCUGCUUUGG
               ➔
AAUCUCAAAUAG AAAUGGGCAACA UAGUUAAACCCA AAGUACUUACGA AAGAAGCAGAGG

AAAAACUUCCUU CUGAUACAGAGA AAGAGGACAGAU CCCUGACAGCUG UAUUGUCAGCAG 2400
  K  L  P  S   D  T  E  K   E  D  R  S   L  T  A  V   L  S  A  E
AGCUGAAUAAAA CUUCAG                                              2418
  L  N  K  T   S [D]
```

C. Nucleotide sequence of circRtn4.
The nucleotides contributed by exon 2 are shown in blue and those from exon 3 in black lettering; the junction is depicted by a vertical arrow. Due to the circular nature of the RNA, the nucleotide at position 2418 is fused to nucleotide 1. If this circular RNA is translated, it generates a GAU codon (highlighted in yellow) coding for aspartic acid, denoted by a [D]. All possible AUG start codons are shown in green and stop codons in red. Where start and stop codons overlap, only the non-overlapping parts of stop codons are red. The numbers above AUG codons indicates the amino acid lengths of hypothetical reading frames, while the 16 AUG codons marked by horizontal arrows are hypothetically proceeding uninterruptedly by any stop codon, thus circling the RNA template. Circumstantial evidence points to a start at the second AUG marked by a green arrow. Part of the amino acid sequence of the open reading frame is shown in the one-letter code beneath the nucleic acid sequence. Nucleotides are arranged in blocks of 12.

D.

```
Frame 3
ETLFALPAASEPVIPSSAEKIMDLKEQPGNTVSSGQEDFPSVLFETAASLPSLSPLSTVSFKEHGYLGN
LSAVASTEGTIEETLNEASRELPERATNPFVNRESAEFSVLEYSEMGSSFNGSPKGESAMLVENTKEEV
IVRSKDKEDLVCSAALHNPQESPATLTKVVKEDGVMSPEKTMDIFNEMKMSVVAPVREEYADFKPFEQA
WEVKDTYEGSRDVLAARANMESKVDKKCFEDSLEQKSHGKDSESRNENASFPSTPELVKDGSRAYITCD
SFTSATESTAANIFPVLEDHTSENKTDEKKIEERKAQIITEKTSPKTSNPFLVAIHDSEADYVTTDNLS
KVTEAVVATMPEGLTPDLVQEACESELNEATGTKIAYETKVDLVQTSEAIQESIYPTAQLCPSFEEAEA
TPSPVLPDIVMEAPLNSLLPSTGASVAQPSASPLEVPSPVSYDGIKLEPENPPPYEEAMSVALKTSDAK
EEIKEPESFNAAVQEAEAPYISIACDLIKETKLSTEPSPEFSNYSEIAKFEKSVPDHCELVDDSSPESE
PVDLFSDDSIPDVPQTQEEAVMLMKESLTEVSETVTQHKHKERLSASPQEVGKPYLESFQPNLHITKDA
ASNEIPTLTKKETISLQMEEFNTAIYSNDDLLSSKEDKMKESETFSDSSPIEIIDEFPTFVSAKDDSPK
EYTDLEVSNKSEIANVQSGANSLPCSELPCDLSFKNTYPKDEAHVSDEFSKSRSSVSKVPLLLPNVSAL
ESQIEMGNIVKPKVLTKEAEEKLPSDTEKEDRSLTAVLSAELNKTS
```

```
Frame 2
-DPFCSSCCI-ACDTLLCRKNYGFEGAAR-HCFVWSRGFPICPV-NCCLSSFSISSLNCFF-RTRIPW-
LISSGIHRRNY-RNFK-SF-RIAREGNKSICK-RVSRVFSIRILRNGIIFQWLPKRRVSHVSRKH-
```

GRSNCEE-RQRGFSL-CSPS-STRVTCDPY-SG-RRRSYVSRKDNGHF--NENVSGSTCEGRVCRF-
AI-TSMGSERYL-GK-GCAGC-S-YGK-SGQKML-R-PGAKKSWEG--KQK-
ECFFPQYPRTCEGRLQSVHHL-FLYLSNREYCSKHFPCARRSHFRK-NR-KKNRRKEGPNYNRED-
PQNVKSFPCSNT-FRGRLCHNR-FIKGD-GSSGNHA-RSNARFSSGSM-K-TERSHRYKDCL-
NKSGLGPDIRSYTRVNLPHSTALPII-GS-SNSVTSFA-
YCYGSSIKFSPSKHWCFCSAAQCIPTRSTVSS-L-RYKA-A-KSPTI-RSHECSTKNIGRKGRN-RA-
KF-CSCSGSRSSLYIHCM-FN-RNKALH-AKSRVL-LFRNSKI-EVGA-SL-ARG-FLTGI-TS-LI--
-FNS-RPTNTRGGCDANEGESH-SV-DSNTTQT-GET-CFTSGGRKAIFRVFSAQFTYYKRCCI--
NSNIDQKGDNFFANGRV-YCNLFQ--LTFF-GRQNERK-NIFRFISH-DNR-VSHICQC-R-FS-GVH-
PRSIQQK-NC-CPERGQFVALLRIAL-PFFQEYIS-R-STCLR-ILQK-VQCI-
GALIASKCFCFGISNRNGQHS-TQSTYERSRGKTSF-YRERGQIPDSCIVSRAE-NF

Frame 1
MRPFLLFLLHLSL-YPPLQKKLWI-
RSSQVTLFRLVKRISHLSCLKLLPLFLLYLLSQLFLLKNTDTLVTYQQWHPQKELLKKL-
MKLLENCQRGQQIHL-IESQQSFQY-NTQKWDHLSMAPQKESQPC--KTLRKK-L-GVKTKRI-
FVVQPFIIHKSHLRPLLKWLKKTELCLQKRQWTFLMK-KCQW-HL-GKSMQILSHLNKHGK-
KILMREVGMCWLLELIWKVKWTKNALKIAWSKKVMGRIVKAEMRMLLSPVPQNL-
RTAPERTSPVIPLPQQPRVLQQTFSLC-KITLQKIKQMKKK-KKGRPKL-QRRLAPKRQILSL-
QYMIPRQIMSQQIIYQR-LRQ-WQPCLKV-RQI-FRKHVKVN-
TKPQVQRLLMKQKWTWSRHQKLYKSQFTPQHSFAHHLRKLKQLRHQFCLILLWKLH-ILSFQALVLL-
RSPVHPH-KYRLQLVMTV-SLSLKIPHHMKKP-V-H-
KHRTQRKKLKSLKVLMQLFRKQKLLIYPLHVI-LKKQSSPLSQVQSSLIIQK-
QNLRSRCLITVSSWMIPHRNLNQLTYLVMIQFLTSHKHKRRL-C--RRVSLKCLRQ-
HNTNIRRDLVLHLRR-ESHI-SLFSPIYILQKMLHLMKFQH-
PKRRQFLCKWKSLILQFIPMMTYFLLRKTK-KKVKHFPIHLPLR--MSFPHLSVLKMILLRSTLT-
KYPTKVKLLMSRAGPIRCLAQNCPVTFLSRIHILKMKHMSQMNSPKVGPVYLRCPYCFQMFLLWNLK-
KWAT-LNPKYLRKKQRKNFLLIQRKRTDP-QLYCQQS-IKLQ

D. circRtn4 computationally translated in all three reading frames.
Only frame 3 is open. Methionine residues are highlighted in green and longer potential ORFs in other reading frames of hypothetical polypeptides are highlighted in blue.

A. circRtn4-FLAG-Stop

```
AUGAGACCCUUU UUGCUCUUCCUG CUGCAUCUGAGC CUGUGAUACCCg actacaaggacg   60
..E  T  L  F   A  L  P  A   A  S  E  P   V  I  P  D   Y  K  D  D

                                          ➔
acgatgacaagc caccgtgaUCCU CUGCAGAAAAAA UUAUGGAUUUGA AGGAGCAGCCAG  120
  D  D  K  P   P  -                      M  D  L  K   E  Q  P  G...
```

B. circRtn4-FLAG-ac

```
AUGAGACCCUUU UUGCUCUUCCUG CUGCAUCUGAGC CUGUGAUACCCg actacaaggacg   60
  E  T  L  F   A  L  P  A   A  S  E  P   V  I  P  D   Y  K  D  D

acgatgacaagc caccgacUCCUC UGCAGAAAAAAU UAUGGAUUUGcA GGAGCAGCCAGG  120
  D  D  K  P   P  T  P  L   Q  K  K  L   W  I  C  R   S  S  Q  V
                                        M  D  L  Q   E  Q  P  G

UAACACUGUUUC GUCUGGUCAAGA GGAUUUCCCAUC UGUCCUGUUUGA AACUGCUGCCUC  180
  T  L  F  R   L  V  K  R   I  S  H  L   S  C  L  K   L  L  P  L
 N  T  V  S   S  G  Q  E   D  F  P  S   V  L  F  E   T  A  A  S
UCUUCCUUCUCU AUCUCCUCUCUC AACUGUUUCUUU UAAAGAACACGG AUACCUUGGUAA  240
  F  L  L  Y   L  L  S  Q   L  F  L  L   K  N  T  D   T  L  V  T
 L  P  S  L   S  P  L  S   T  V  S  F   K  E  H  G   Y  L  G  N
CUUAUCAGCAGU GGCAUCCACAGA AGGAACUAUUGA AGAAACUUUAAA UGAAGCUUCUAG  300
  Y  Q  Q  W   H  P  Q  K   E  L  L  K   K  L  -
 L  S  A  V   A  S  T  E   G  T  I  E   E  T  L  N   E  A  S ... etc.
```

Supplementary Fig. 2

Additional constructs circRtn4-Stop (A) and circRtn4_FLAG-ac (B). Blue lettering indicates nucleotide sequence contributions of *Rtn4* exon 2 and black upper-case lettering contributions of *Rtn4* exon 3. Nucleotides are arranged in blocks of 12. The nucleotides for the FLAG sequence are in black lower-case letters. The corresponding amino acids are depicted in bold and purple (IUPAC one-letter amino acid code). The putative start codon in green. A) A translation stop codon was introduced after the sequence encoding the FLAG peptide, presumably after almost a full circle of translation (indicated in red). The predicted Rtn4 polypeptide variant (IUPAC one-letter amino acid code) is ~800 amino acids in length. B) The insertion of two nucleotides (ac) at positions 78/79 and a single nucleotide exchange at position 107 (c) are shown in lower case letters highlighted in blue. The correspondingly changed amino acid Q at position 4 (highlighted in blue) of the assumed Rtn4-derived amino acid sequence is also highlighted in blue. The latter was changed in order to remove a stop codon early in the second round of translation. The predicted polypeptide sequence is given in the IUPAC one-letter amino acid code. Translation product for the first round on the circle is shown in the second aa sequence row. Translation could theoretically proceed for about another ~69 amino acid into a next round of translation, provided that AUG start codon 98-100 is being used for translation initiation. The amino acid sequence of the second round of translation would leave the reading frame after the FLAG peptide at the glutamine

residue and proceed (top amino acid sequence immediately under the nucleotide sequence) until the stop codon at position 285-287 (red) is encountered. The predicted protein would be ~869 amino acids in length.

Supplementary Fig. 3

16 mass spectra of the tryptic peptides from circRtn4 derived protein

The left coordinate is the relative intensity, the right coordinate is the absolute intensity, the horizontal coordinate is m/z.

Scan	Method	Score	m/z
16346	FTMS; HCD	144.1	632.28

Supplementary Fig. 3
1 of 16 peptide mass spectra

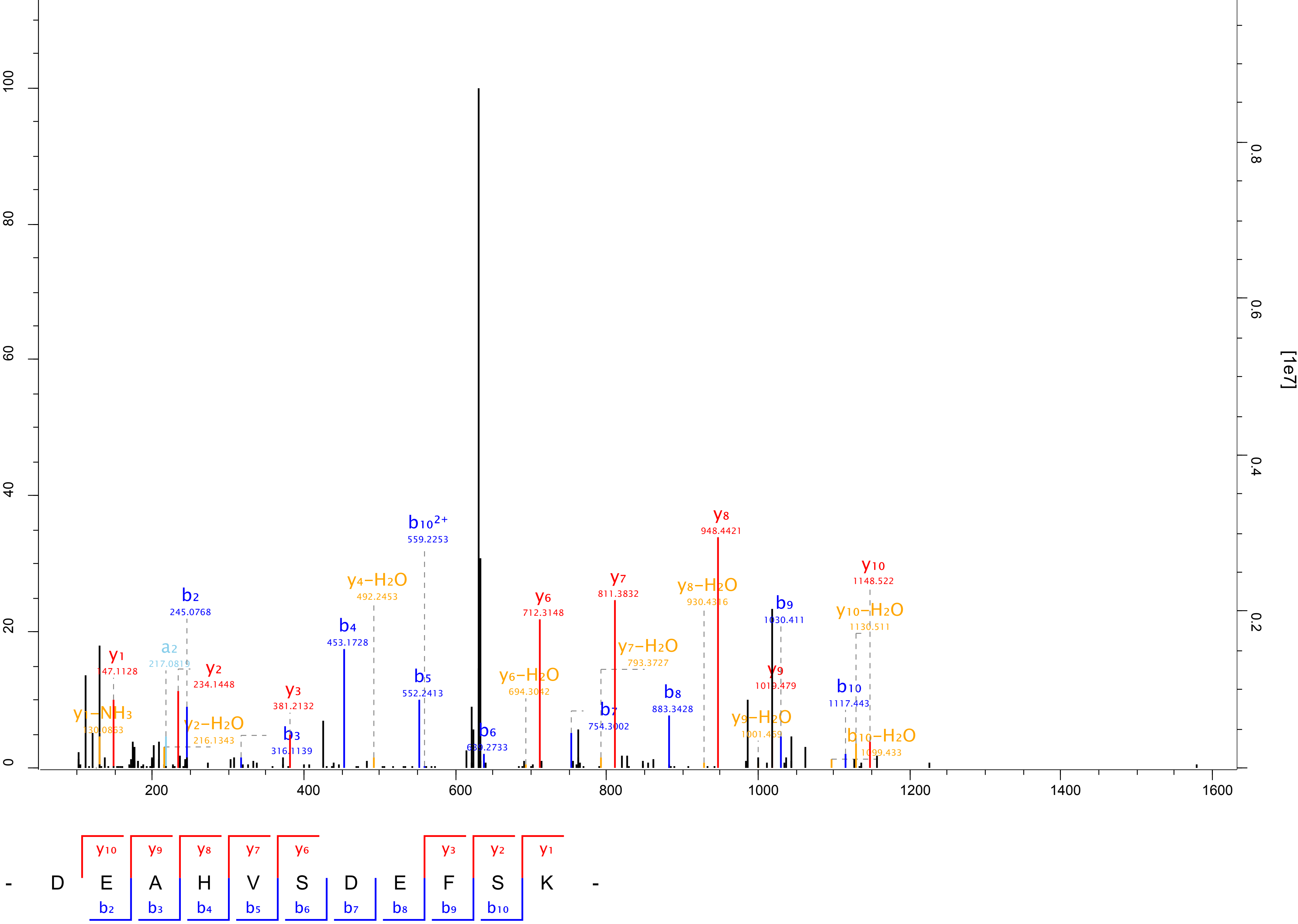

Scan	Method	Score	m/z
109034	FTMS; HCD	75.44	1089.21

Supplementary Fig. 3
2 of 16 peptide mass spectra

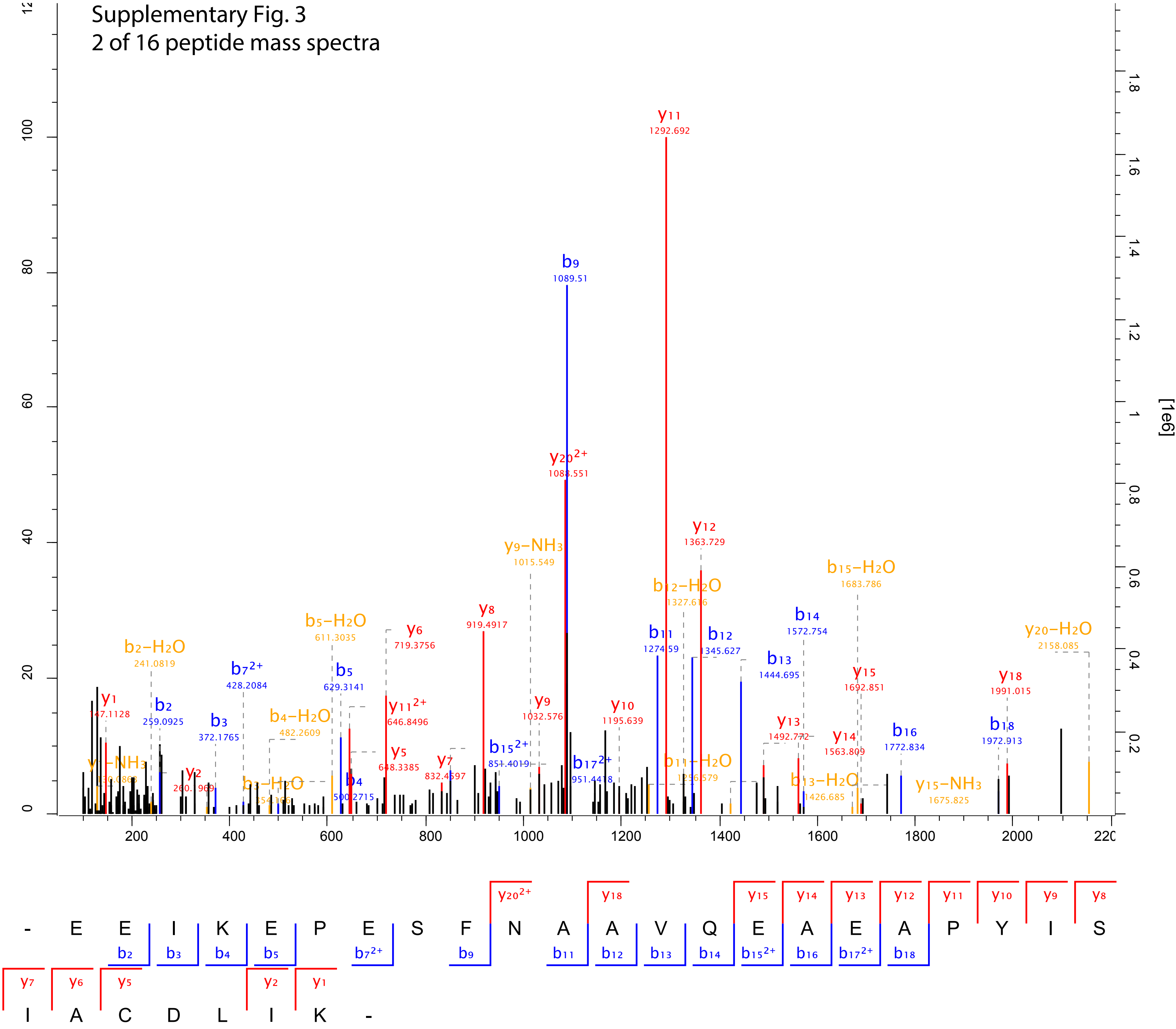

Scan	Method	Score	m/z
88482	FTMS; HCD	62.81	672.99

Supplementary Fig. 3

3 of 16 peptide mass spectra

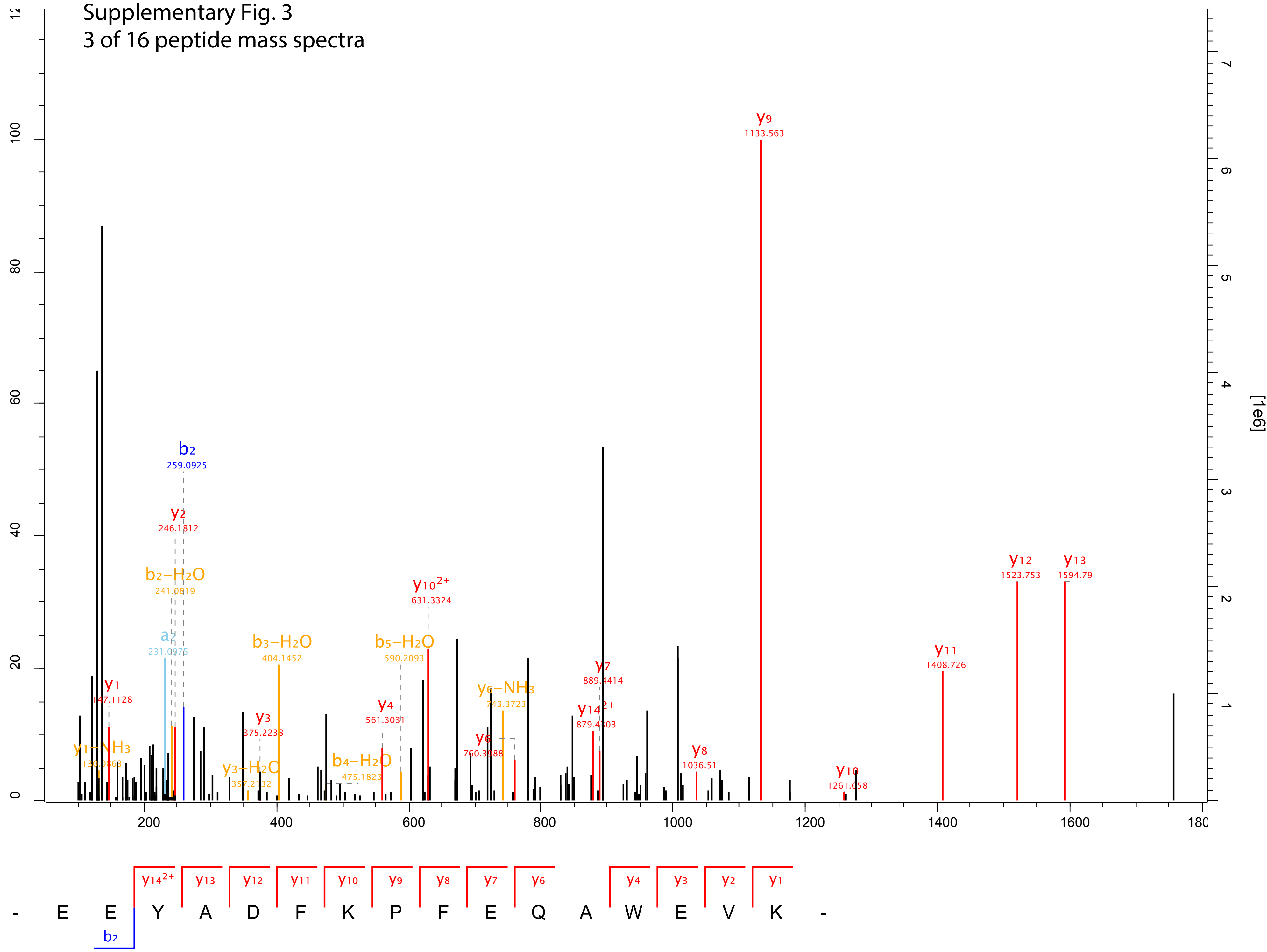

Supplementary Fig. 3
4 of 16 peptide mass spectra

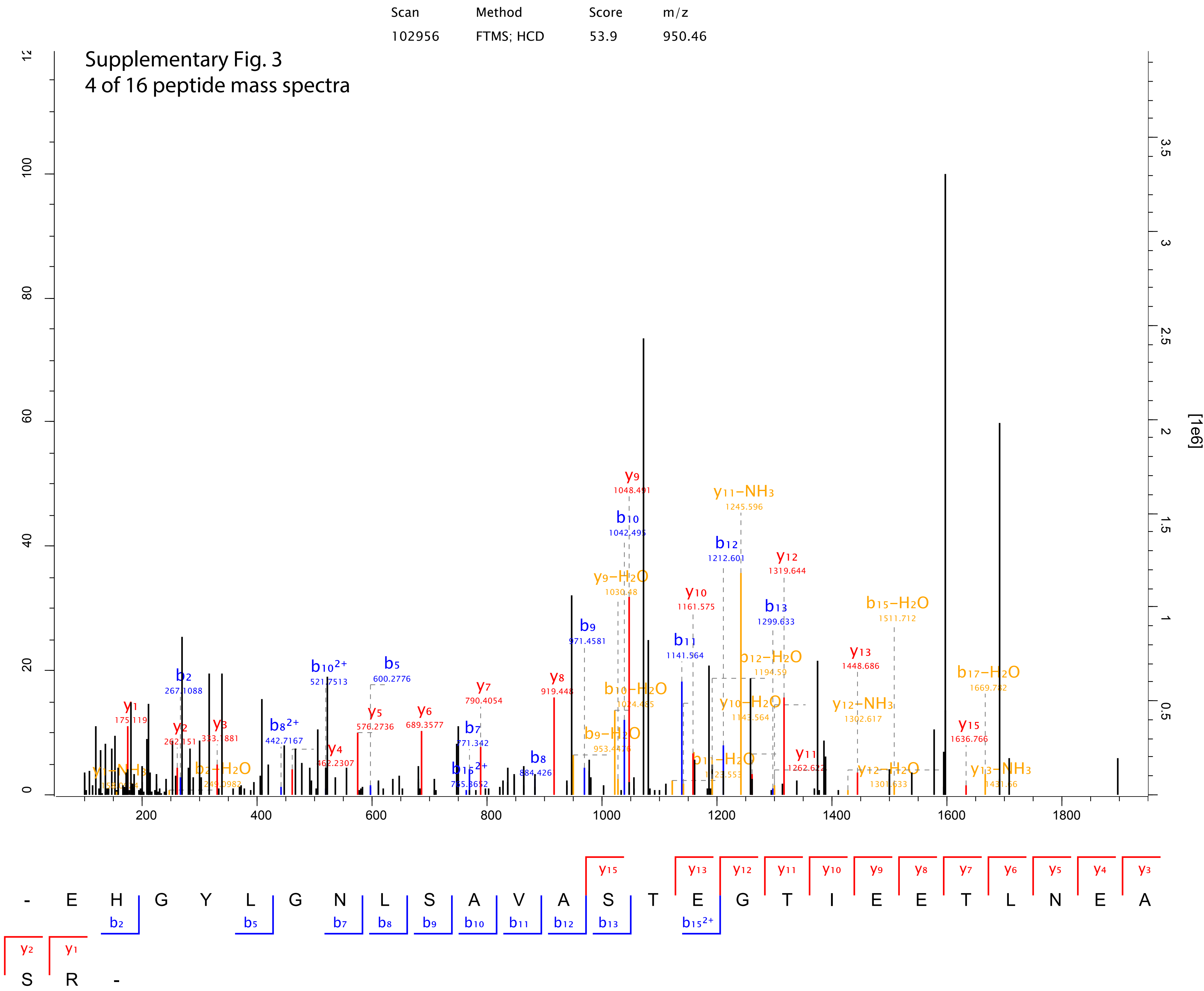

Supplementary Fig. 3
5 of 16 peptide mass spectra

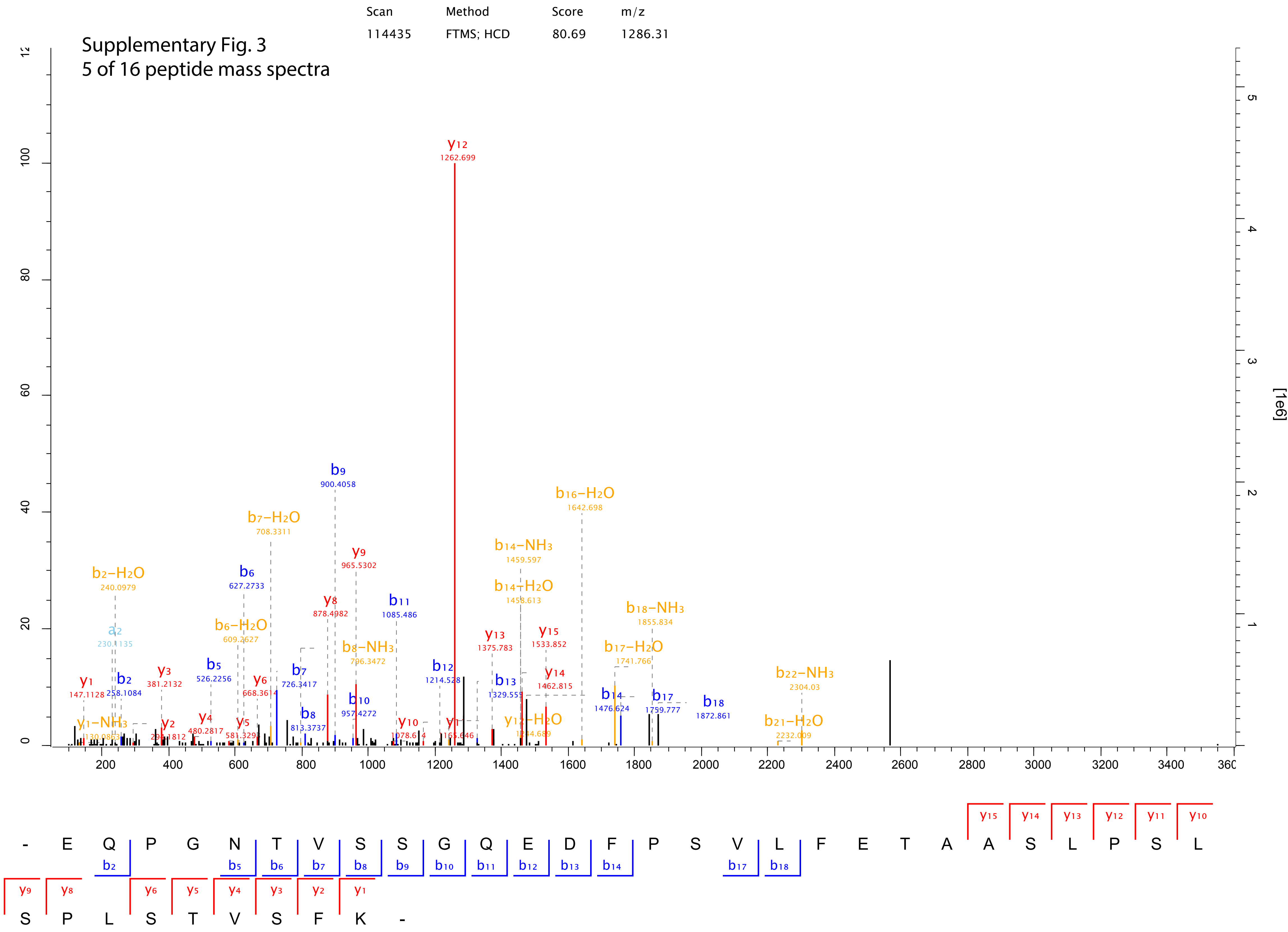

Scan	Method	Score	m/z
40472	FTMS; HCD	69.26	529.93

Supplementary Fig. 3
6 of 16 peptide mass spectra

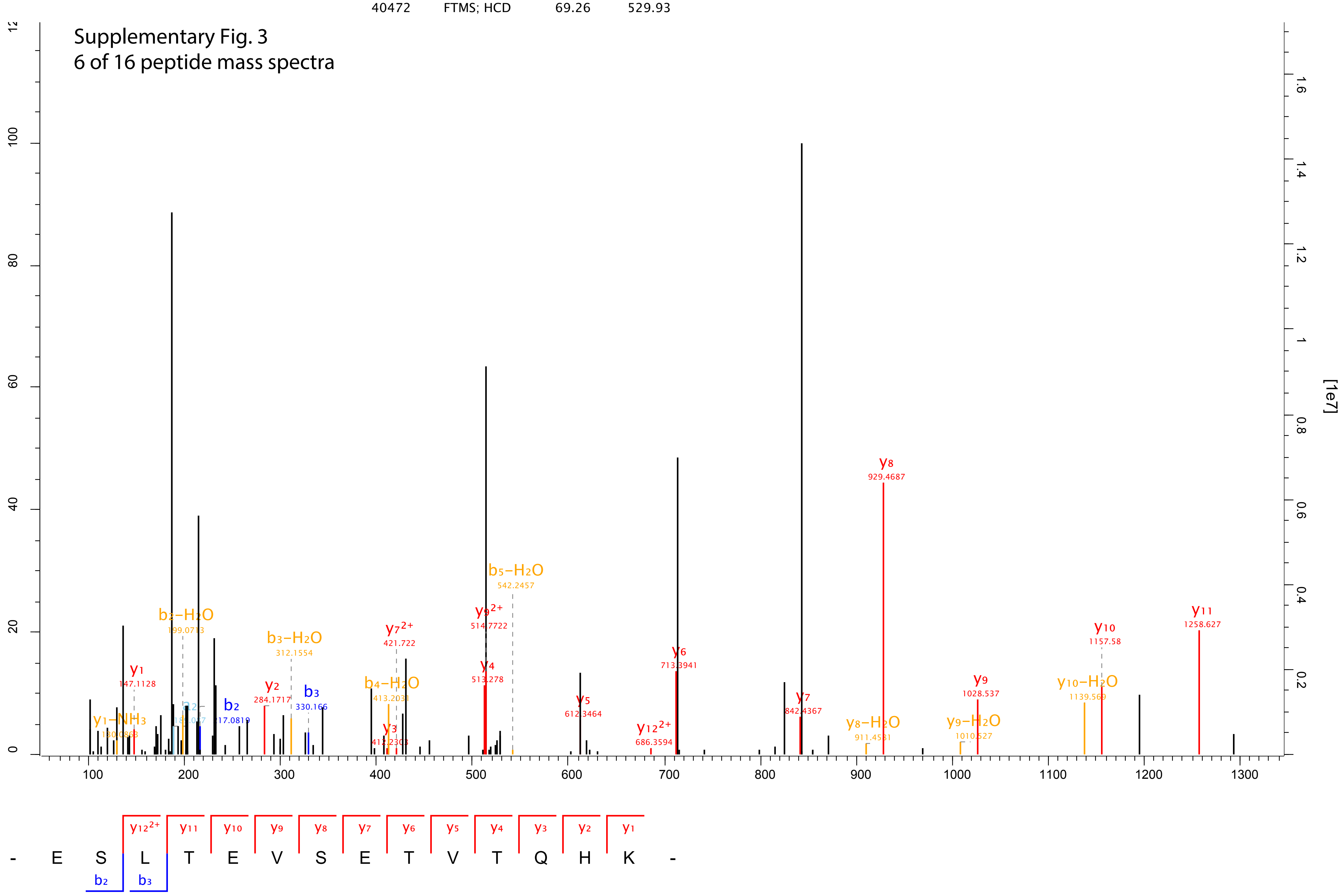

Scan	Method	Score	m/z
26668	FTMS; HCD	88.06	599.29

Supplementary Fig. 3
7 of 16 peptide mass spectra

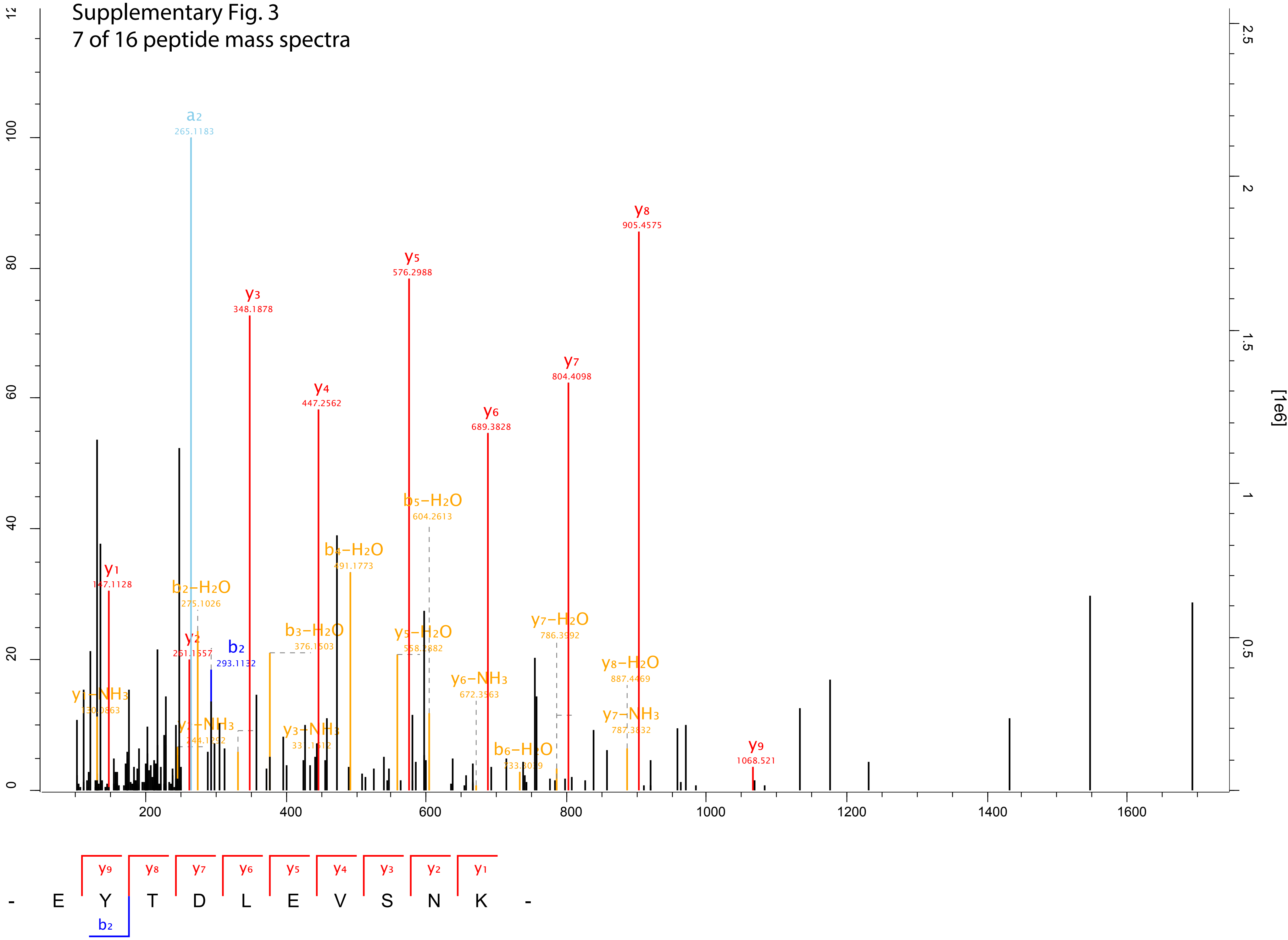

Supplementary Fig. 3
8 of 16 peptide mass spectra

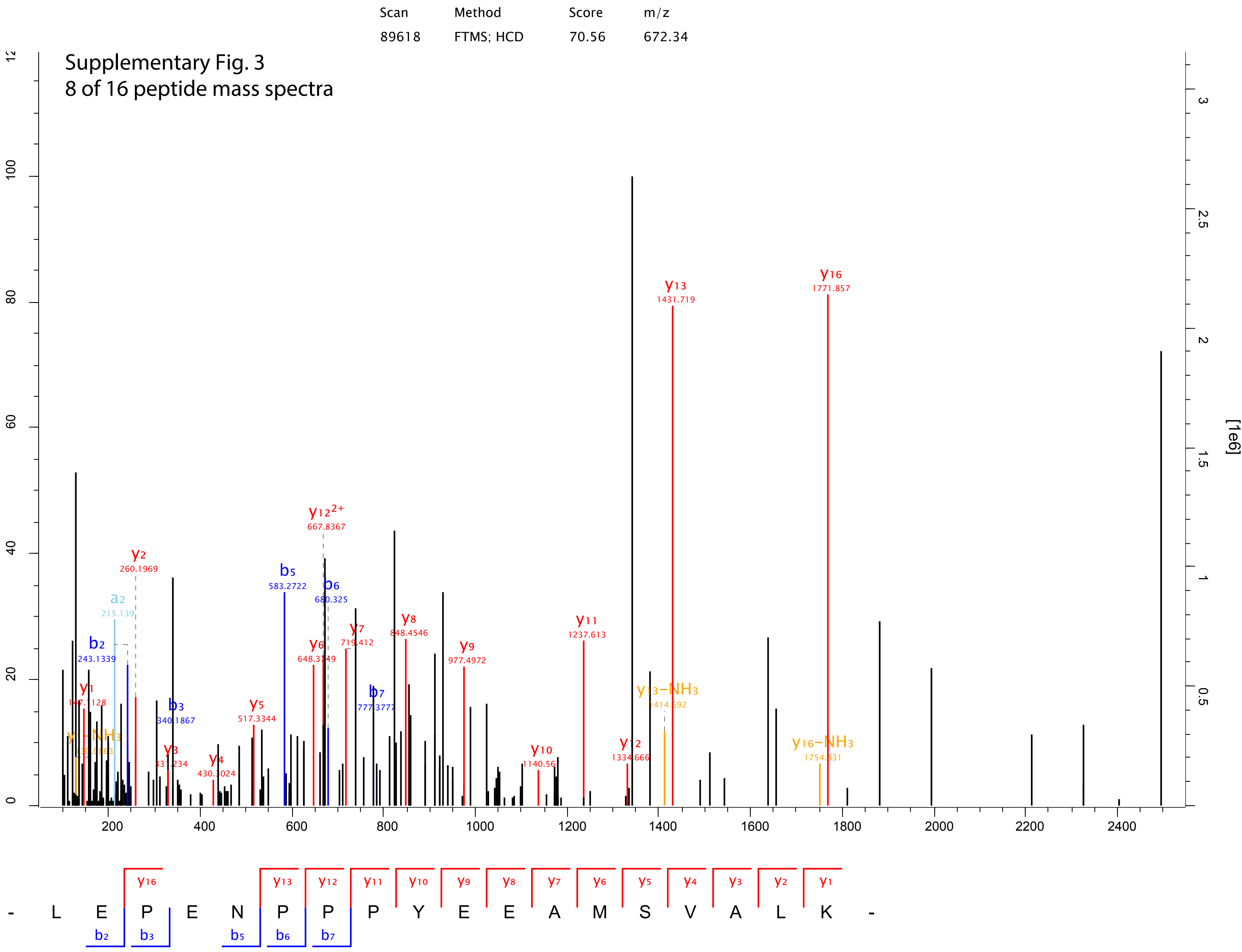

Scan	Method	Score	m/z
64737	FTMS; HCD	105.89	949.96

Supplementary Fig. 3
9 of 16 peptide mass spectra

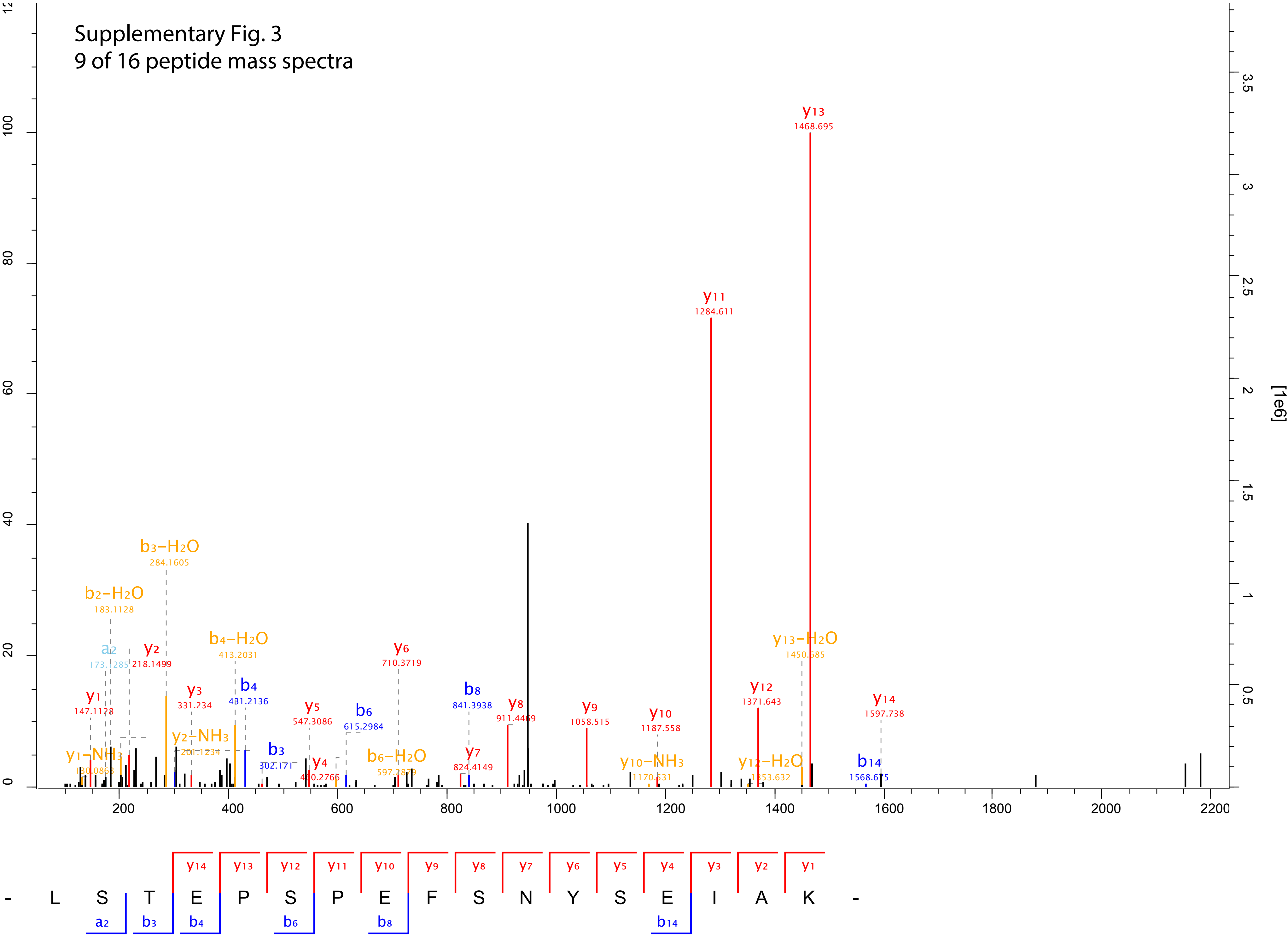

Raw file	Scan	Method	Score	m/z
170901_QE_Li_DMo_6D_DMo_RTN4_02	96317	FTMS; HCD	35.16	952.48

Supplementary Fig. 3
10 of 16 peptide mass spectra

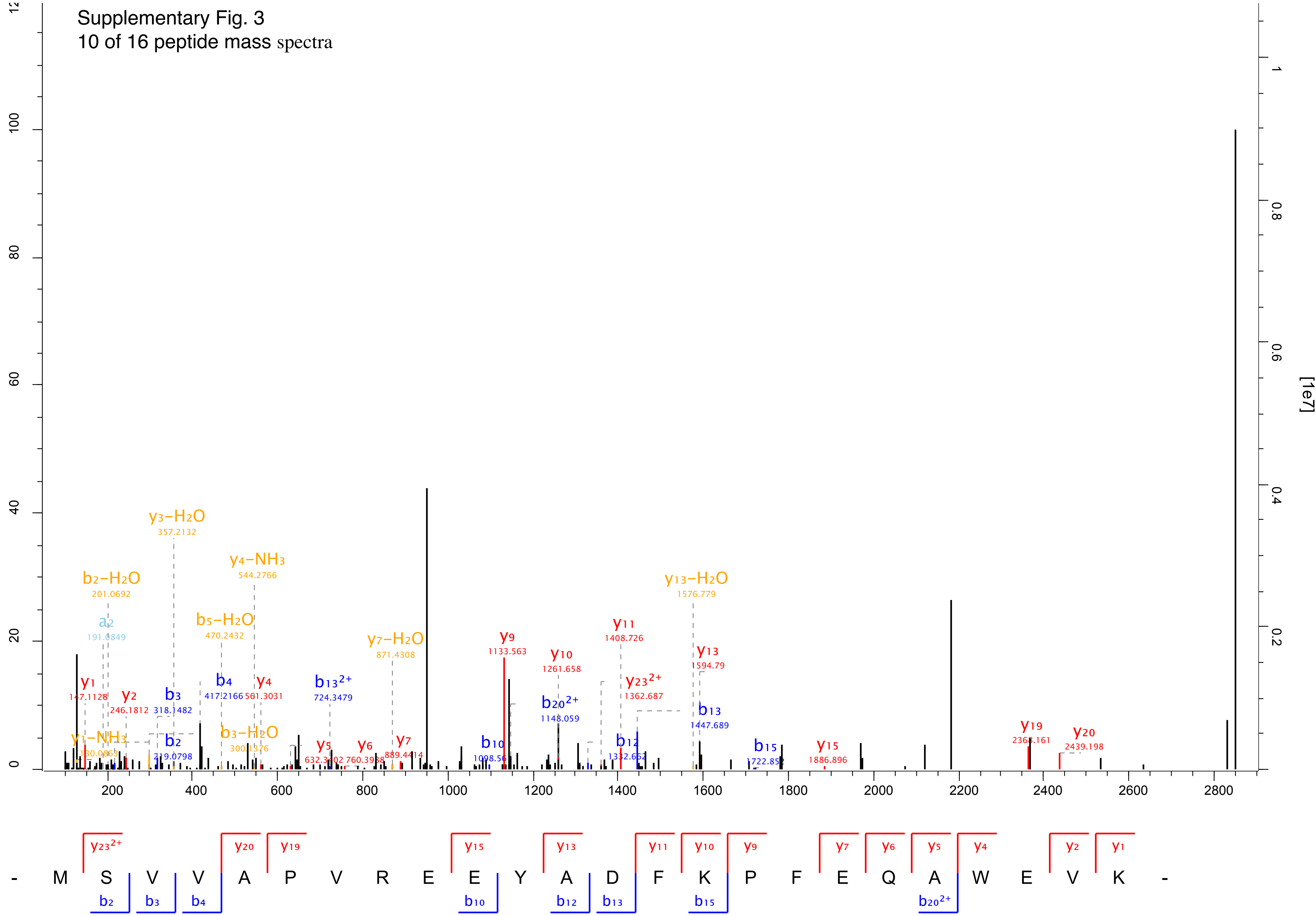

Scan	Method	Score	m/z
58602	FTMS; HCD	146.59	766.89

Supplementary Fig. 3
11 of 16 peptide mass spectra

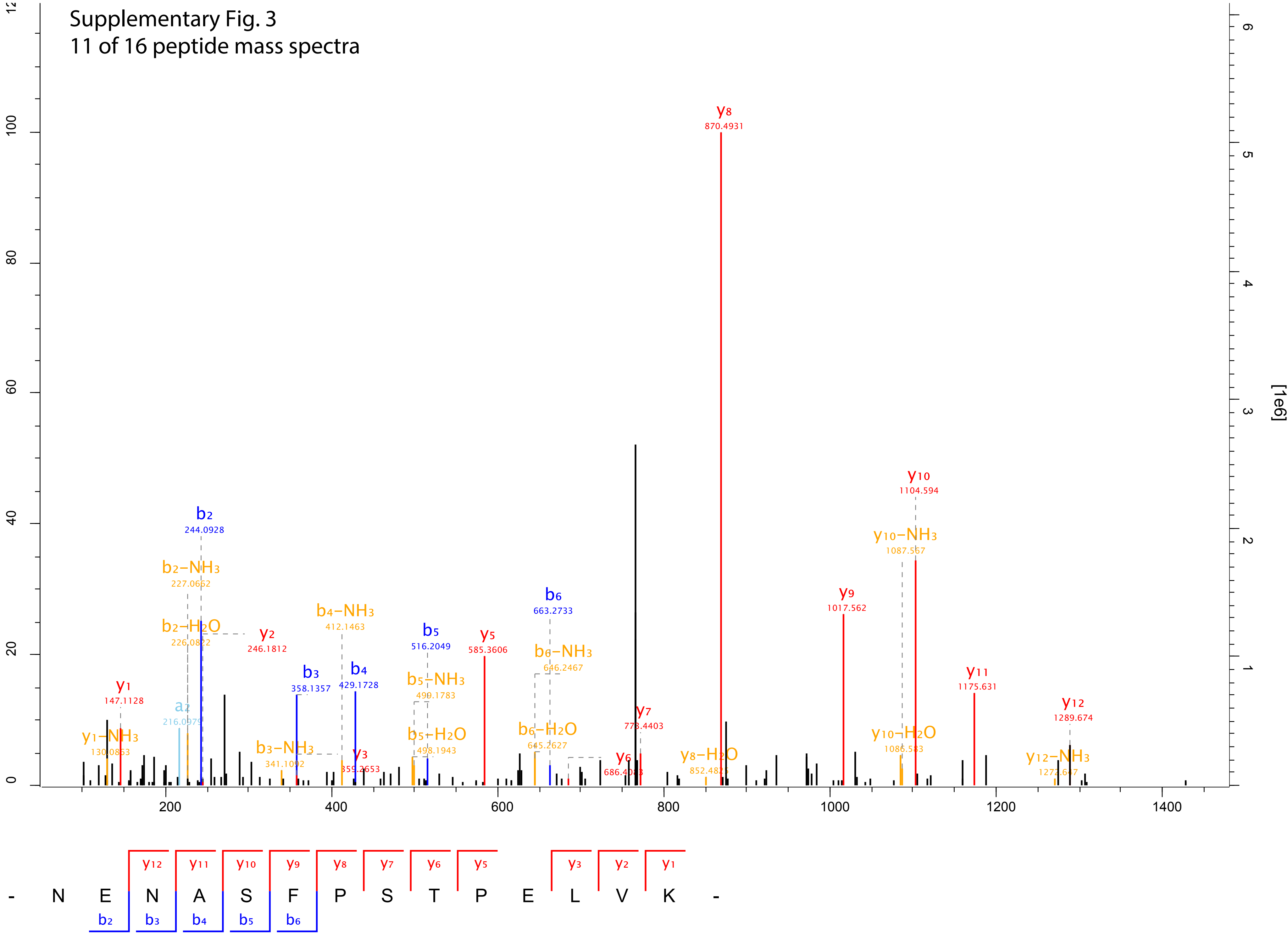

Scan	Method	Score	m/z
93362	FTMS; HCD	74.66	909.1

Supplementary Fig. 3

12 of 16 peptide mass spectra

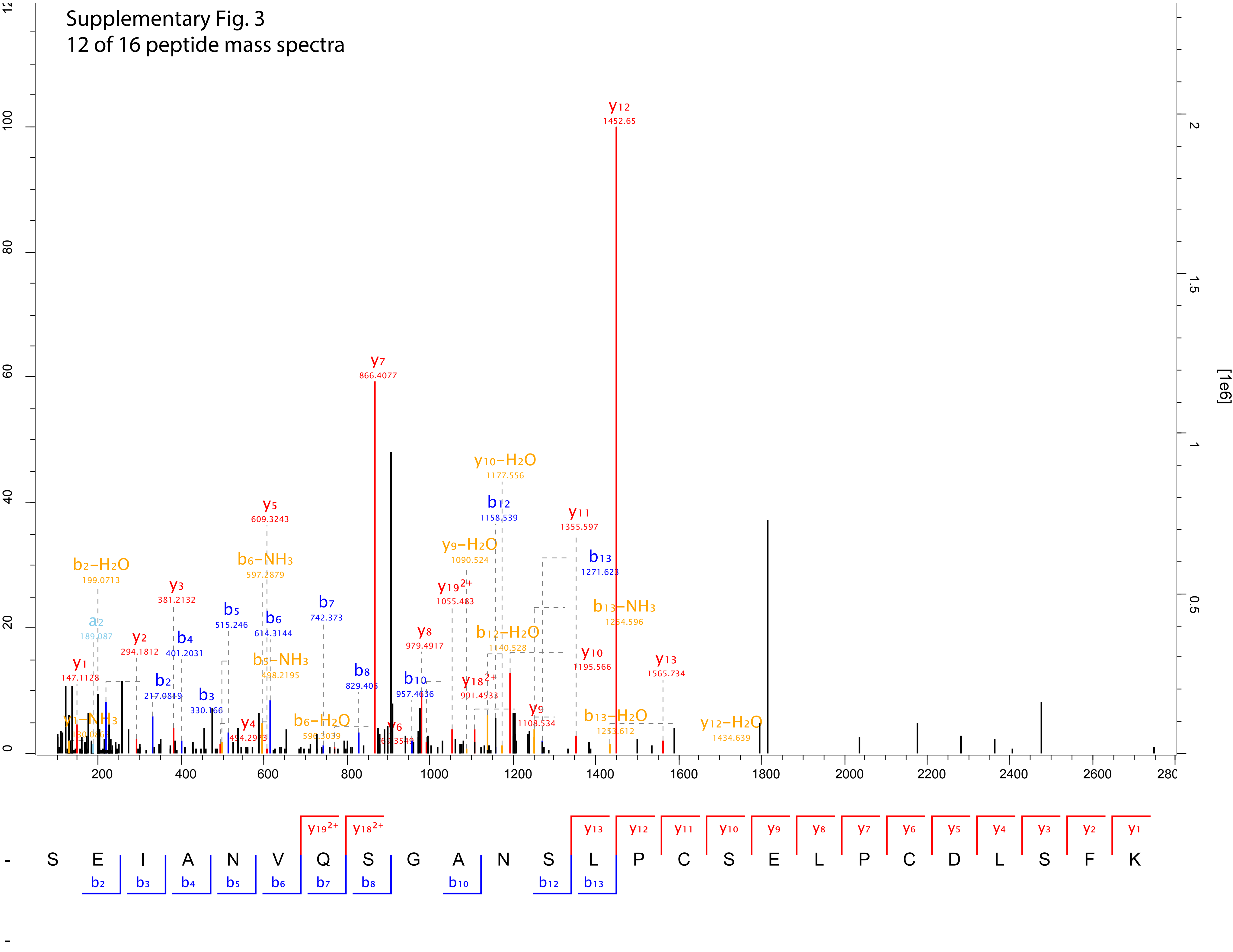

Scan	Method	Score	m/z
41459	FTMS; HCD	179.64	914.46

Supplementary Fig. 3
13 of 16 peptide mass spectra

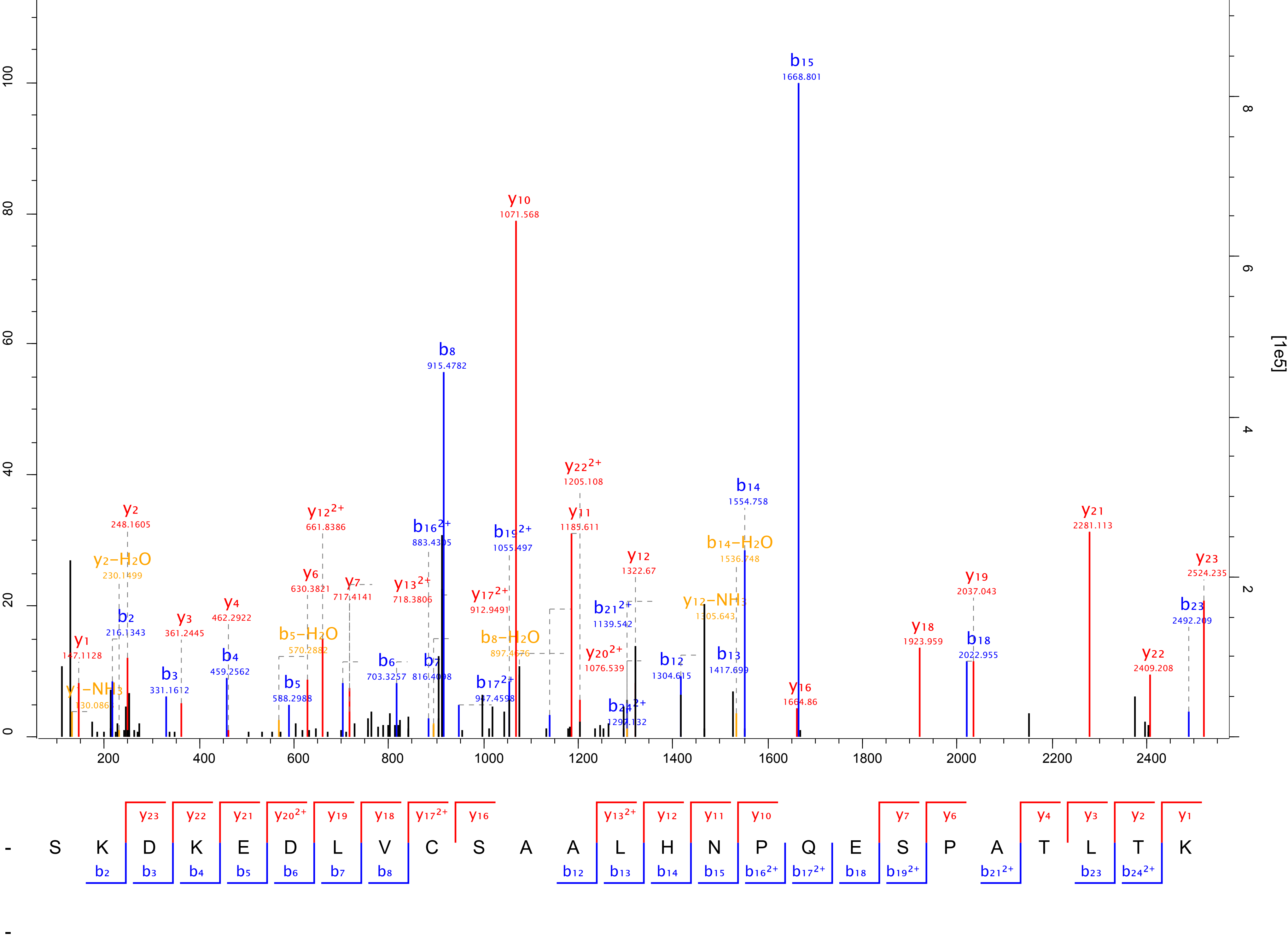

Supplementary Fig. 3
14 of 16 peptide mass spectra

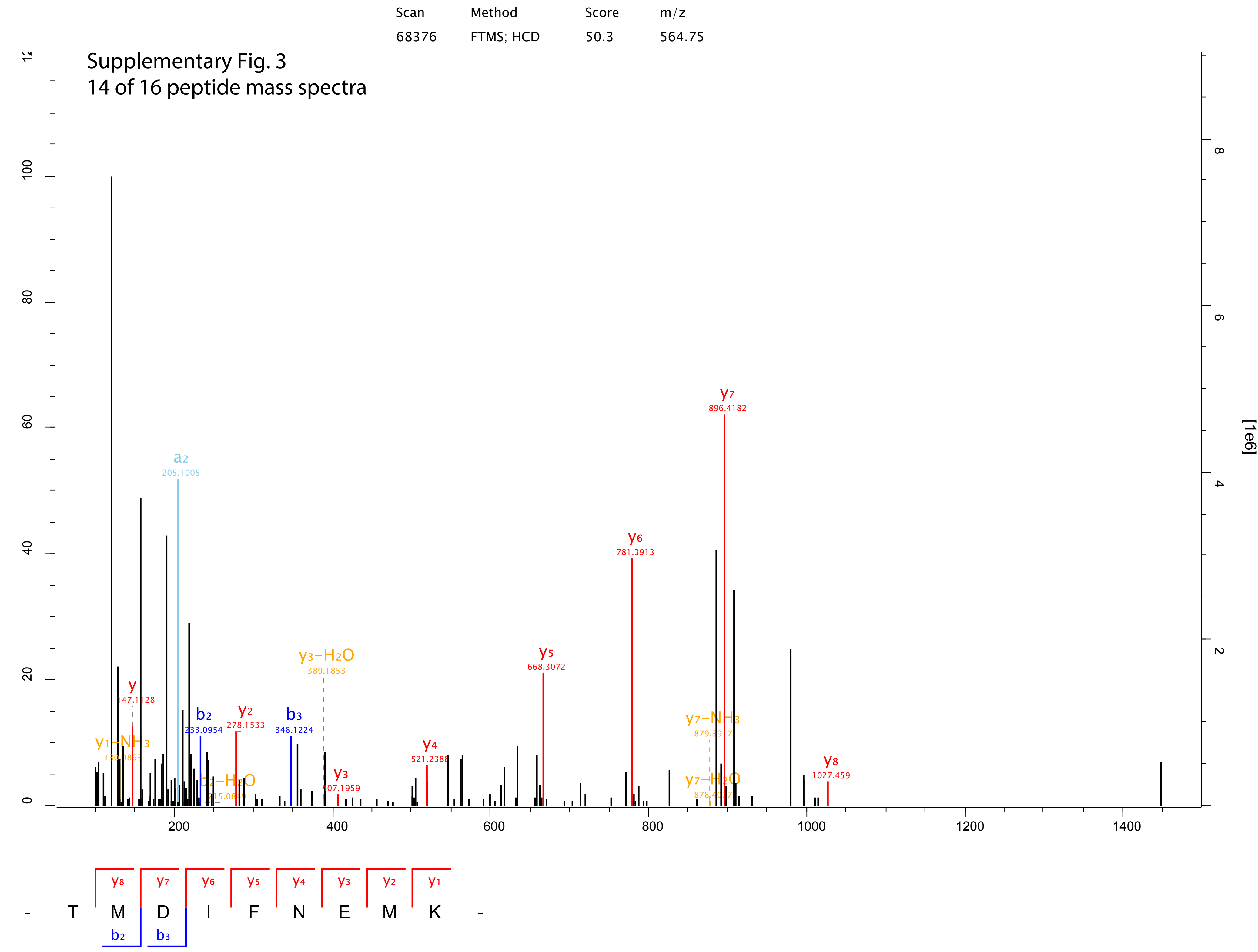

Supplementary Fig. 3
15 of 16 peptide mass spectra

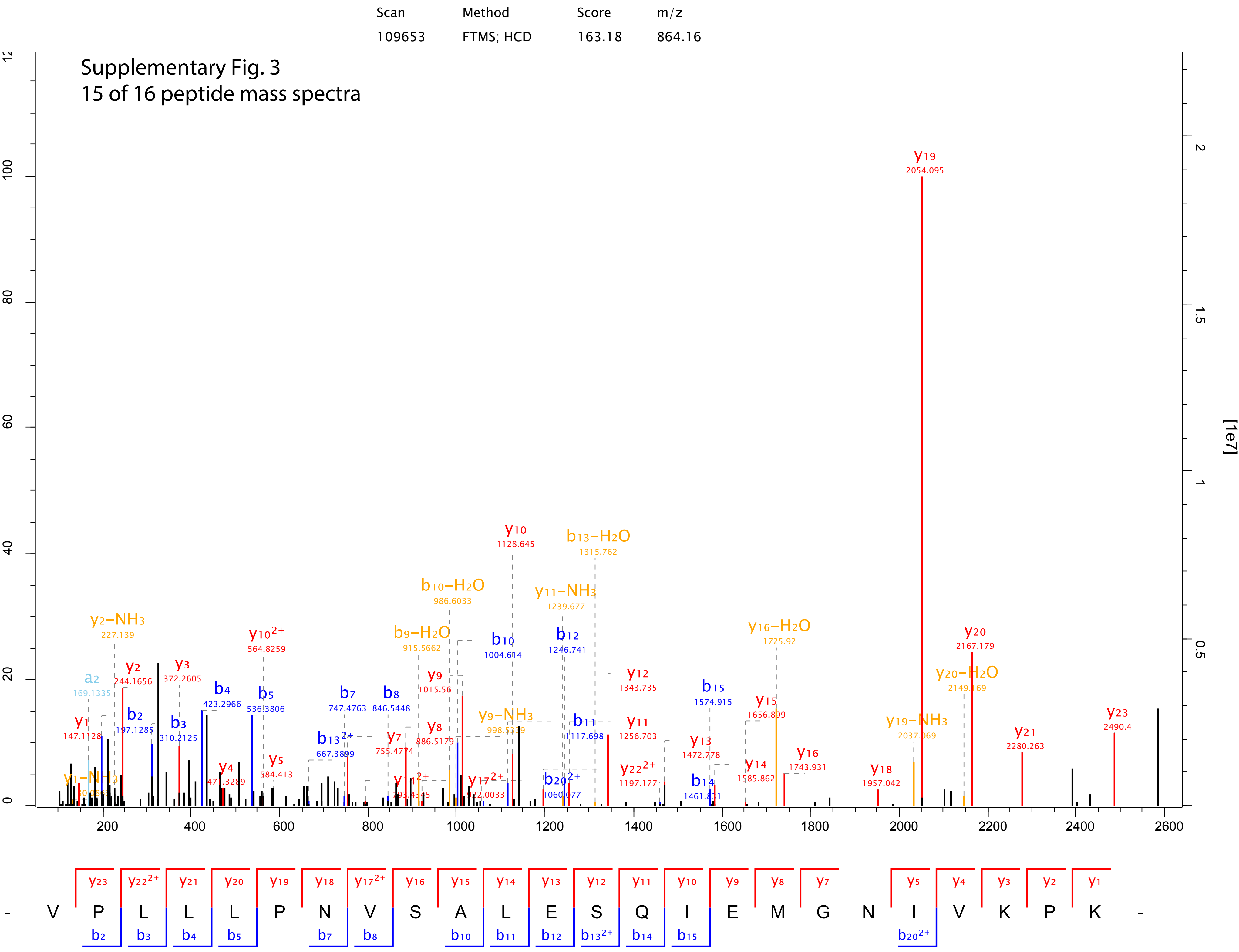

Scan	Method	Score	m/z
11487	FTMS; HCD	114.21	659.34

Supplementary Fig. 3
16 of 16 peptide mass spectra

Supplementary Fig. 6
16 of 52 peptide mass spectra

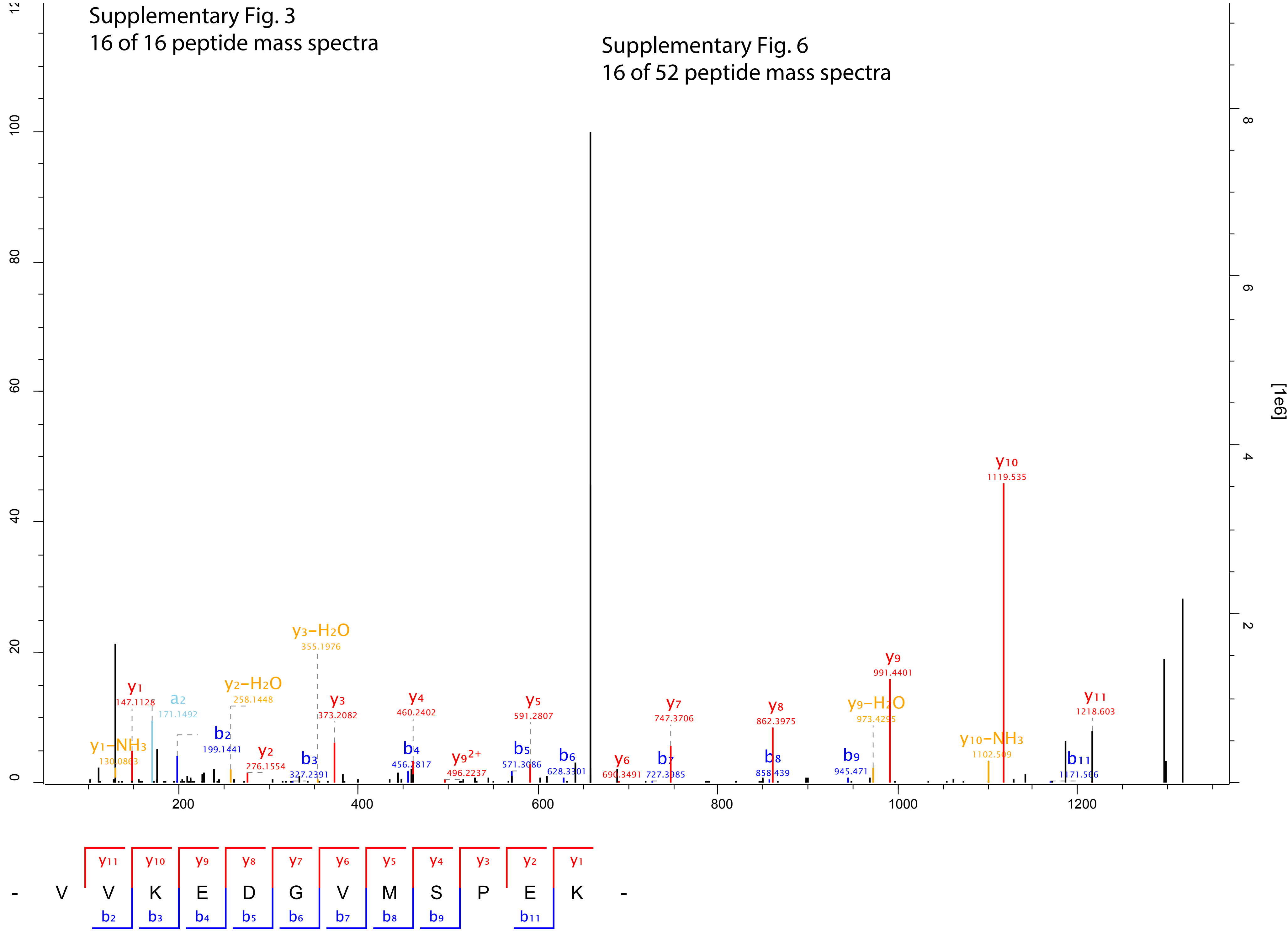

A.

MDLK_EQPGNTVSSGQEDFPSVLFETAASLPSLSPLSTVSFK_EHGYLGNLSAVASTEGTIEE
TLNEASR_ELPERATNPFVNRESAEFSVLEYSEMGSSFNGSPKGESAMLVENTKEEVIVR_S
KDKEDLVCSAALHNPQESPATLTK_VVKEDGVMSPEK_TMDIFNEMK_MSVVAPVREEYAD
FKPFEQAWEVK_DTYEGSRDVLAARANMESKVDKKCFEDSLEQKSHGKDSESR_NENASF
PSTPELVK_DGSRAYITCDSFTSATESTAANIFPVLEDHTSENKTDEKKIEERKAQIITEKTSP
KTSNPFLVAIHDSEADYVTTDNLSKVTEAVVATMPEGLTPDLVQEACESELNEATGTKIA
YETKVDLVQTSEAIQESIYPTAQLCPSFEEAEATPSPVLPDIVMEAPLNSLLPSTGASVAQP
SASPLEVPSPVSYDGIK_LEPENPPPYEEAMSVALK_TSDAK_EEIKEPESFNAAVQEAEAPYIS
IACDLIK_ETK_LSTEPSPEFSNYSEIAK_FEKSVPDHCELVDDSSPESEPVDLFSDDSIPDVPQT
QEEAVMLMK_ESLTEVSETVTQHK_HKERLSASPQEVGKPYLESFQPNLHITKDAASNEIPT
LTKKETISLQMEEFNTAIYSNDDLLSSKEDKMKESETFSDSSPIEIIDEFPTFVSAKDDSPK_
EYTDLEVSNK_SEIANVQSGANSLPCSELPCDLSFK_NTYPK_DEAHVSDEFSK_SRSSVSK_VPLLL
PNVSALESQIEMGNIVKPK_VLTKEAEEKLPSDTEKEDRSLTAVLSAELNKTS**D**ETLFALP
AASEPVIPSSAE**K**I

B.

MDLK.EQPGNTVSSGQEDFPSVLFETAASLPSLSPLSTVSFK_EHGYLGNLSAVASTEGTIEE
TLNEASR_ELPER_ATNPFVNR_ESAEFSVLEYSEMGSSFNGSPK_GESAMLVENTK.EEVIVR_S
K.DKEDLVCSAALHNPQESPATLTK_VVK.EDGVMSPEK_TMDIFNEMK_MSVVAPVR.EEYAD
FKPFEQAWEVK.DTYEGSR.DVLAAR_ANMESKVDKK_CFEDSLEQK_SHGK.DSESR.NENASF
PSTPELVK.DGSR_AYITCDSFTSATESTAANIFPVLEDHTSENKTDEK.K_IEER_KAQIITEKTSP
K_TSNPFLVAIHDSEADYVTTDNLSK_VTEAVVATMPEGLTPDLVQEACESELNEATGTK_IA
YETKVDLVQTSEAIQESIYPTAQLCPSFEEAEATPSPVLPDIVMEAPLNSLLPSTGASVAQP
SASPLEVPSPVSYDGIK_LEPENPPPYEEAMSVALK_TSDAK_EEIKEPESFNAAVQEAEAPYIS
IACDLIK.ETK_LSTEPSPEFSNYSEIAK.FEK_SVPDHCELVDDSSPESEPVDLFSDDSIPDVPQT
QEEAVMLMK_ESLTEVSETVTQHK_HKERLSASPQEVGKPYLESFQPNLHITK_DAASNEIPT
LTK.K_ETISLQMEEFNTAIYSNDDLLSSK.EDK_MK.ESETFSDSSPIEIIDEFPTFVSAK.DDSPK_
EYTDLEVSNK_SEIANVQSGANSLPCSELPCDLSFK_NTYPK.DEAHVSDEFSK_SRSSVSK_VPLLL
PNVSALESQIEMGNIVK.PK_VLTK_EAEEK.LPSDTEKEDR_SLTAVLSAELNK_TS**D**ETLFALP
AASEPVIPdykddddkppSSA**E**K_I

Supplementary Fig. 4 Tryptic peptides identified by mass spectrometry.
A, B. Peptides derived from construct circRtn4 and from circRtn4-FLAG are shown in red and blue, respectively. For mass spectrograms, see Supplementary Figs. 3 and 6, respectively. The amino acids contributed by the FLAG sequence are in lower case. The glutamic acid residue (E) contributed by the splice site between exons 2 and 3 (last row) and the aspartic acid residue (D) at the site of circularization (penultimate row) are shown in bold.
Complete digestion is marked by an underline symbol and observed incomplete digestion yielding two or more overlapping products is marked by a dot, i.e., there were additional sequences (N-terminally and/or C-terminally) in identified peptides beyond the dots.

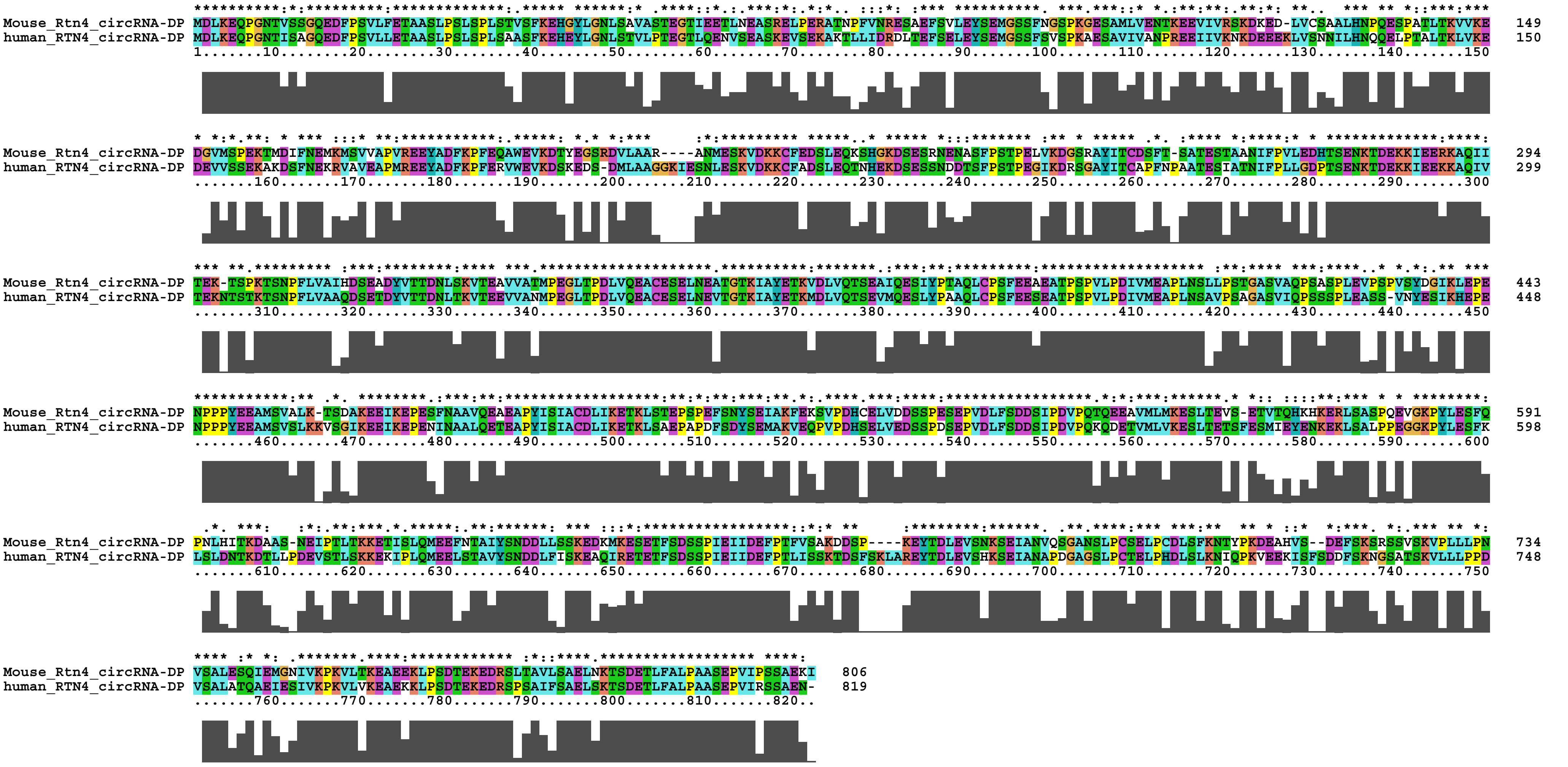

Supplementary Fig. 5

Alignment of circRtn4/RTN4-derived protein sequences from mouse and human. The sequences of mouse circRtn4-derived protein (mouse_Rtn4_circRNA-DP) and human circRtn4-derived protein (human_Rtn4_circRNA-DP) were aligned with the ClustalX2 program.

Supplementary Fig. 6

52 mass spectra of the tryptic peptides from circRtn4-FLAG derived protein

The left coordinate is the relative intensity, the right coordinate is the absolute intensity, the horizontal coordinate is m/z.

Raw file	Scan	Method	Score	m/z
180221_FS1_Li_DMo_cRNA_Try	11808	FTMS; HCD	122.74	459.75

Supplementary Fig. 6
1 of 52 peptide mass spectra

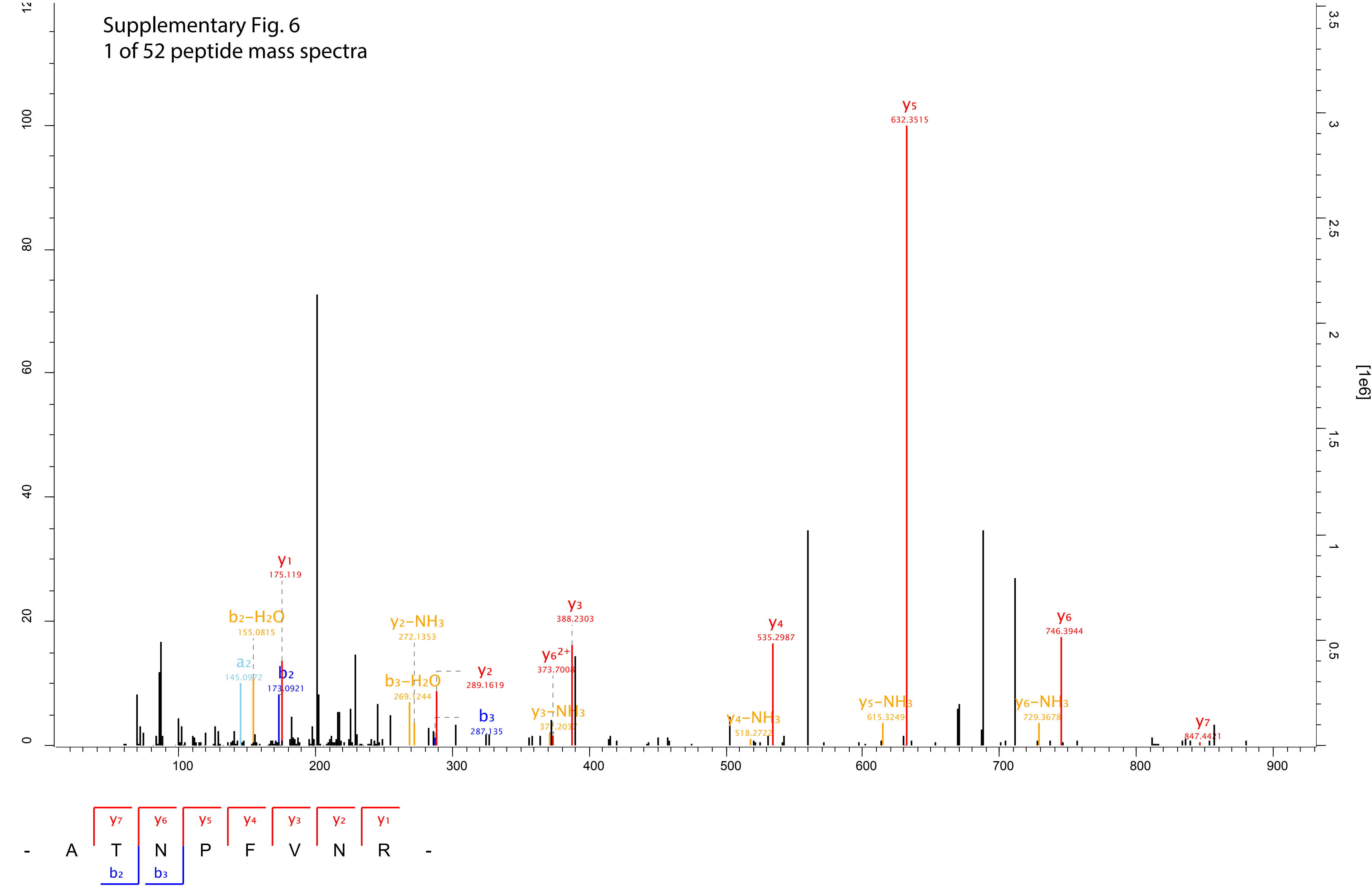

Raw file	Scan	Method	Score	m/z
180221_FS1_Li_DMo_cRNA_Try	35440	FTMS; HCD	96.07	974.46

Supplementary Fig. 6
2 of 52 peptide mass spectra

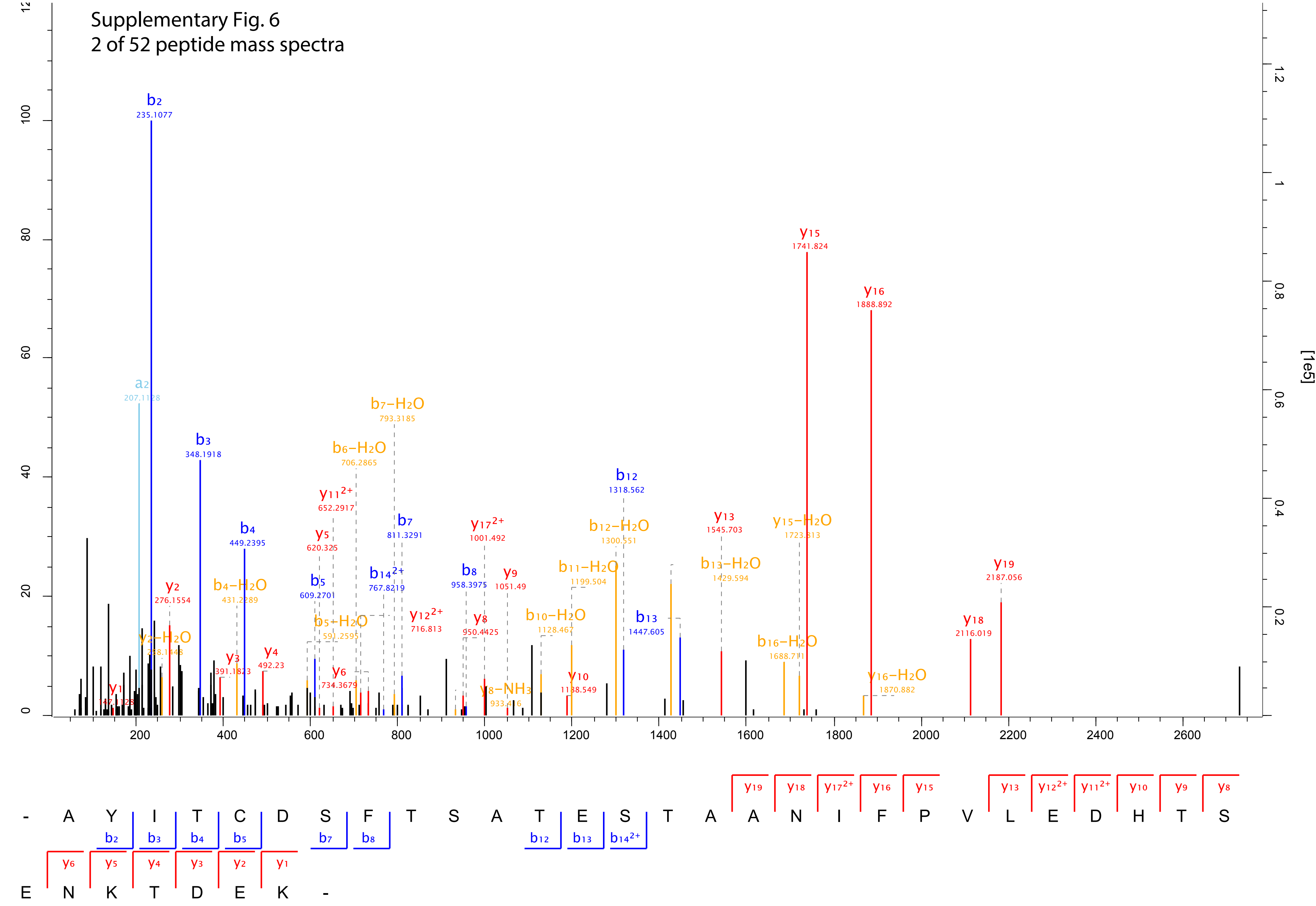

Raw file	Scan	Method	Score	m/z
180221_FS1_Li_DMo_cRNA_Try	34937	FTMS; HCD	30.01	805.39

Supplementary Fig. 6
3 of 52 peptide mass spectra

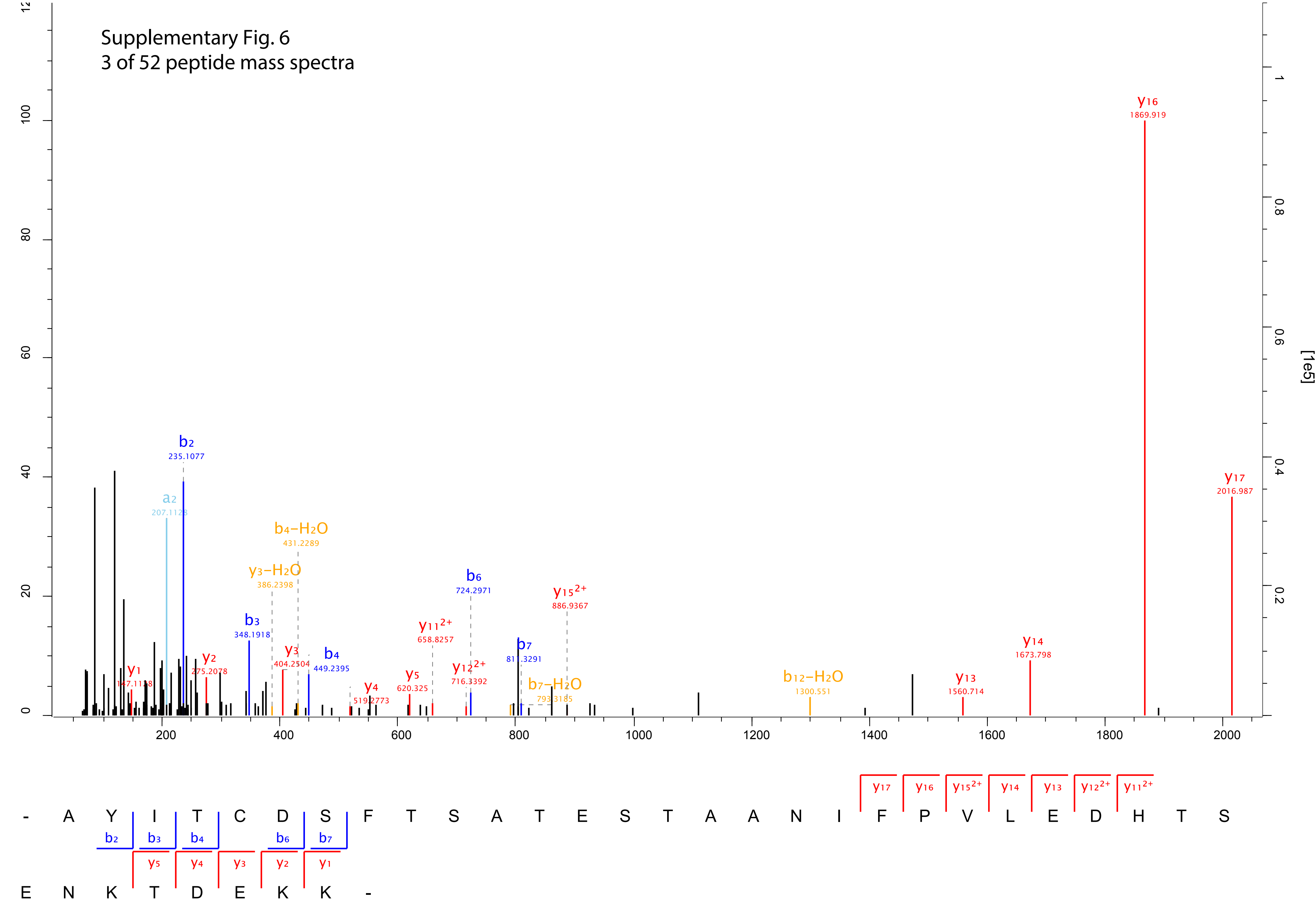

Raw file	Scan	Method	Score	m/z
180221_FS1_Li_DMo_cRNA_Try	13121	FTMS; HCD	196.32	578.26

Supplementary Fig. 6
4 of 52 peptide mass spectra

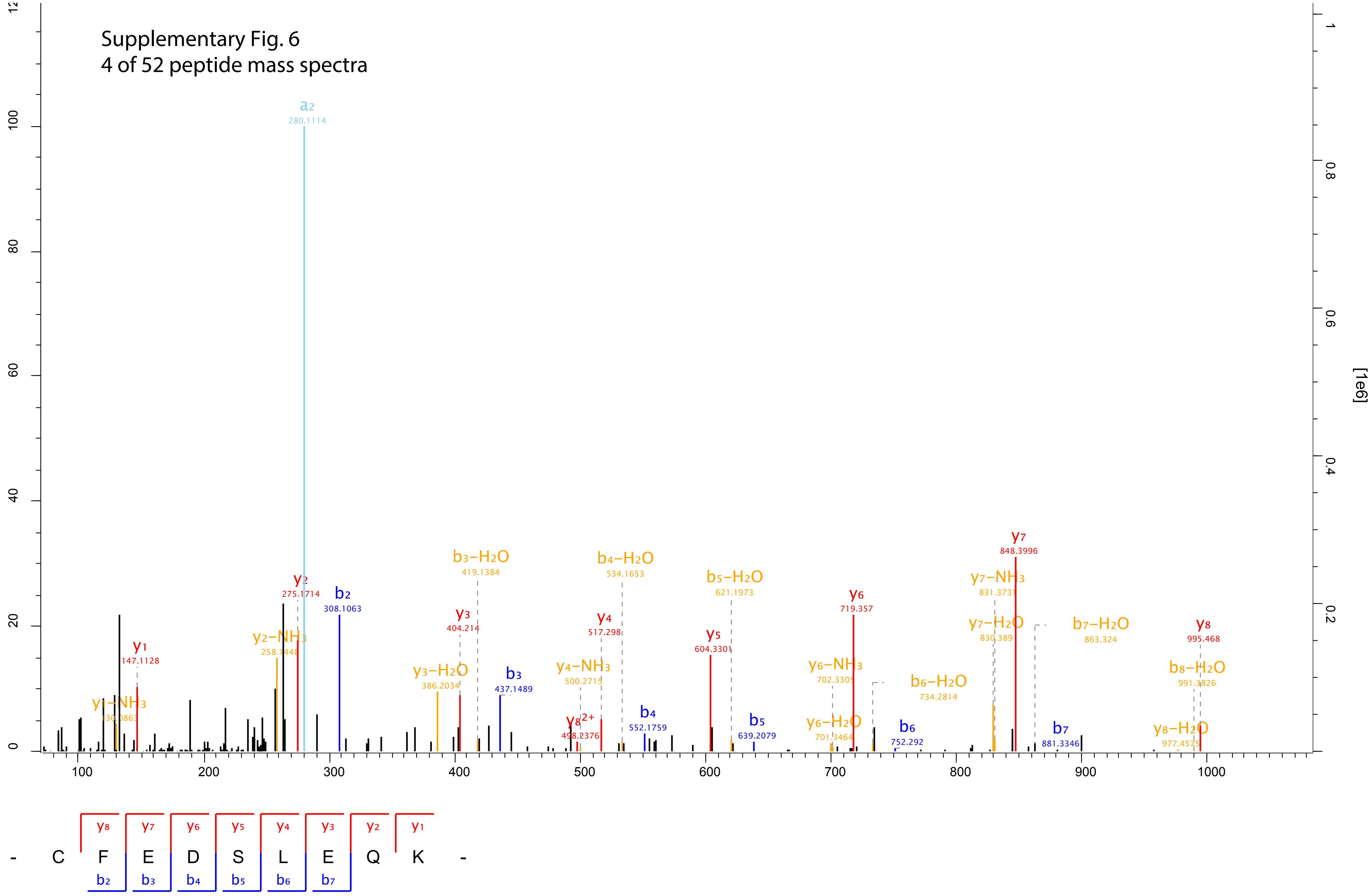

Raw file	Scan	Method	Score	m/z
180221_FS1_Li_DMo_cRNA_F_P	18308	FTMS; HCD	114.72	630.33

Supplementary Fig. 6
5 of 52 peptide mass spectra

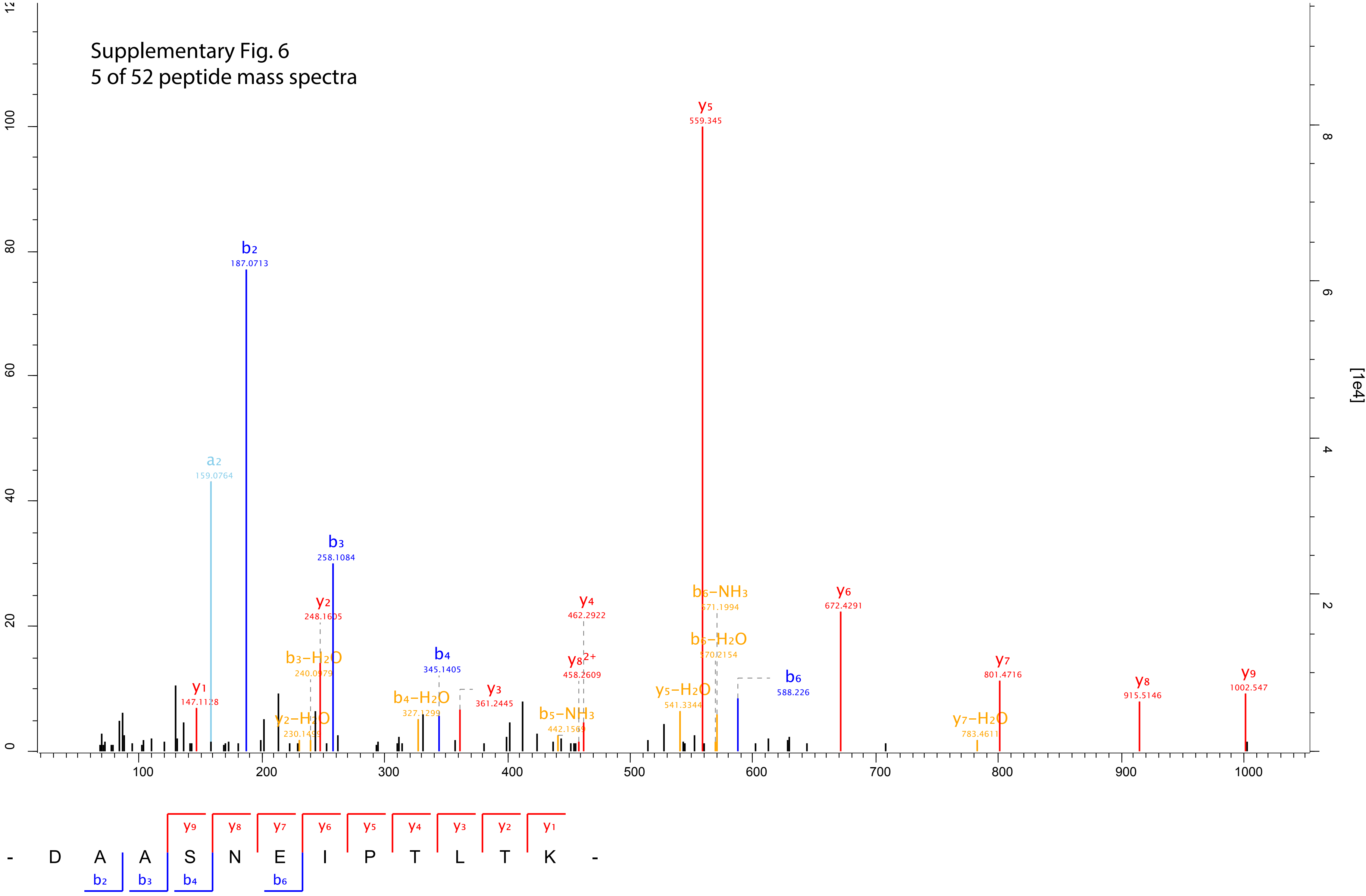

Raw file	Scan	Method	Score	m/z
180221_FS1_Li_DMo_cRNA_Try	15095	FTMS; HCD	99.99	463.26

Supplementary Fig. 6
6 of 52 peptide mass spectra

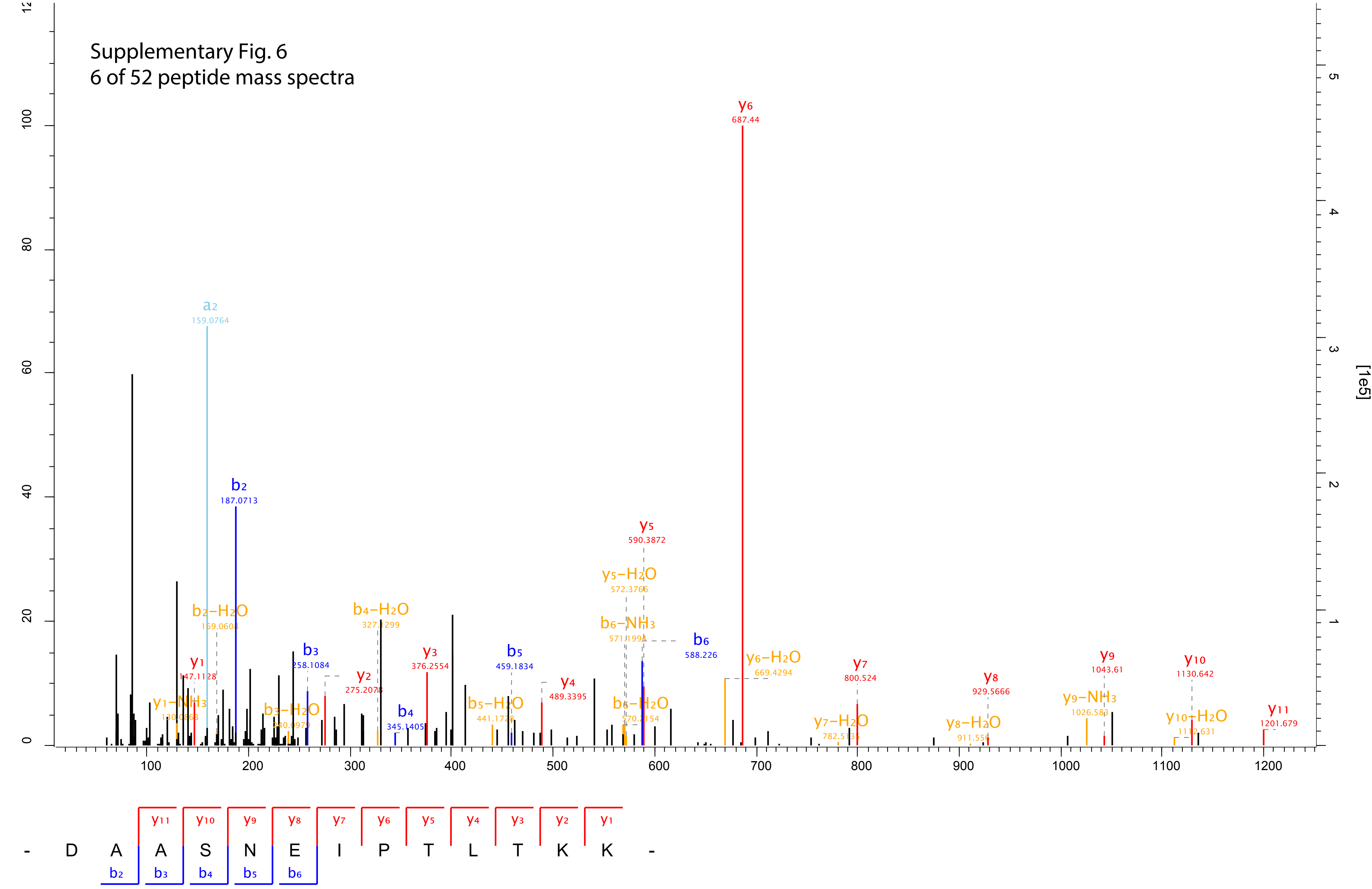

Raw file	Scan	Method	Score	m/z
180221_FS1_Li_DMo_cRNA_Try	15711	FTMS; HCD	138.87	580.61

Supplementary Fig. 6
7 of 52 peptide mass spectra

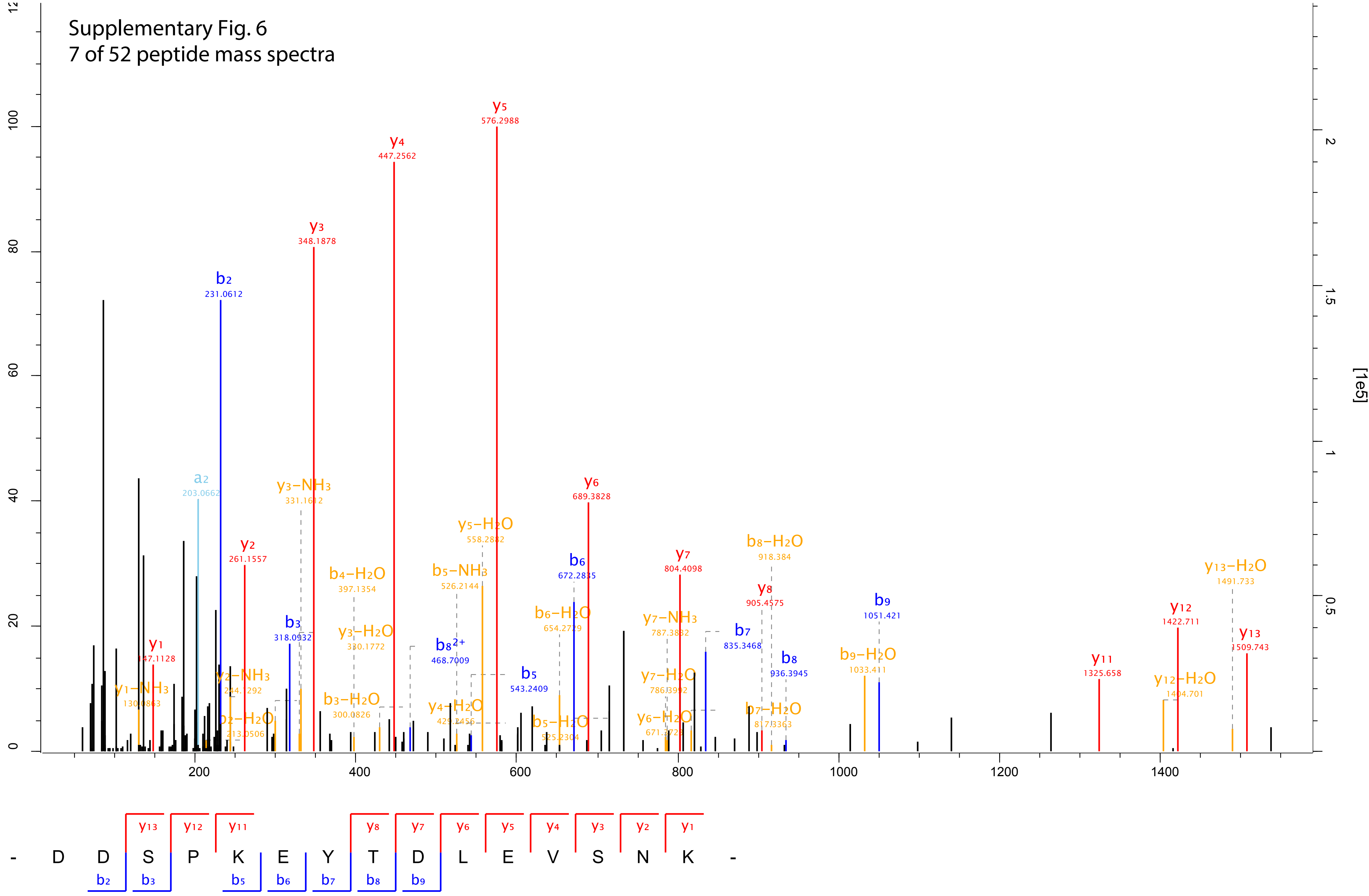

Raw file	Scan	Method	Score	m/z
180221_FS1_Li_DMo_cRNA_F_P	6839	FTMS; HCD	124.6	632.28

Supplementary Fig. 6
8 of 52 peptide mass spectra

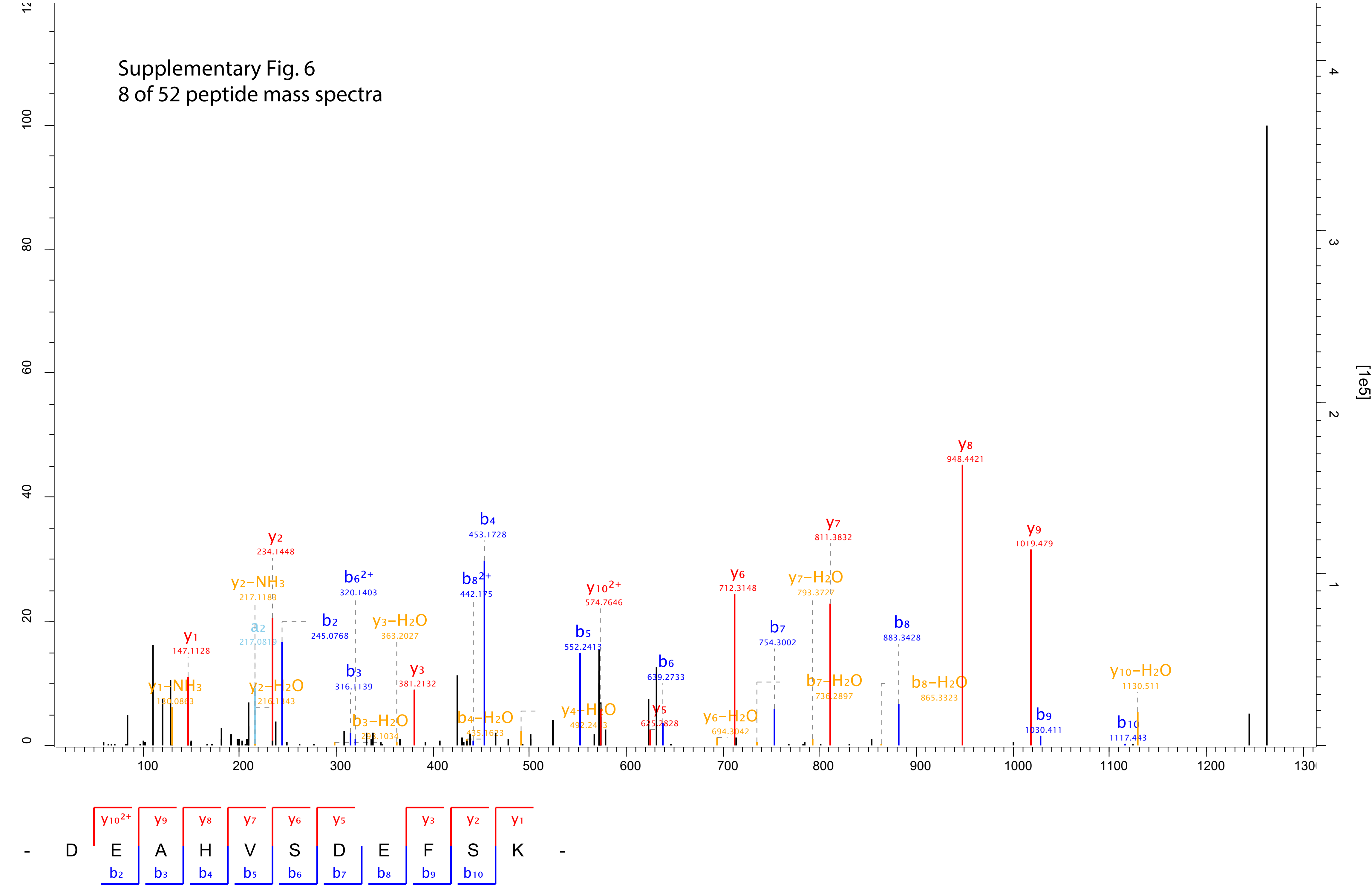

Raw file	Scan	Method	Score	m/z
180221_FS1_Li_DMo_cRNA_Try	21329	FTMS; HCD	126.97	842.42

Supplementary Fig. 6
9 of 52 peptide mass spectra

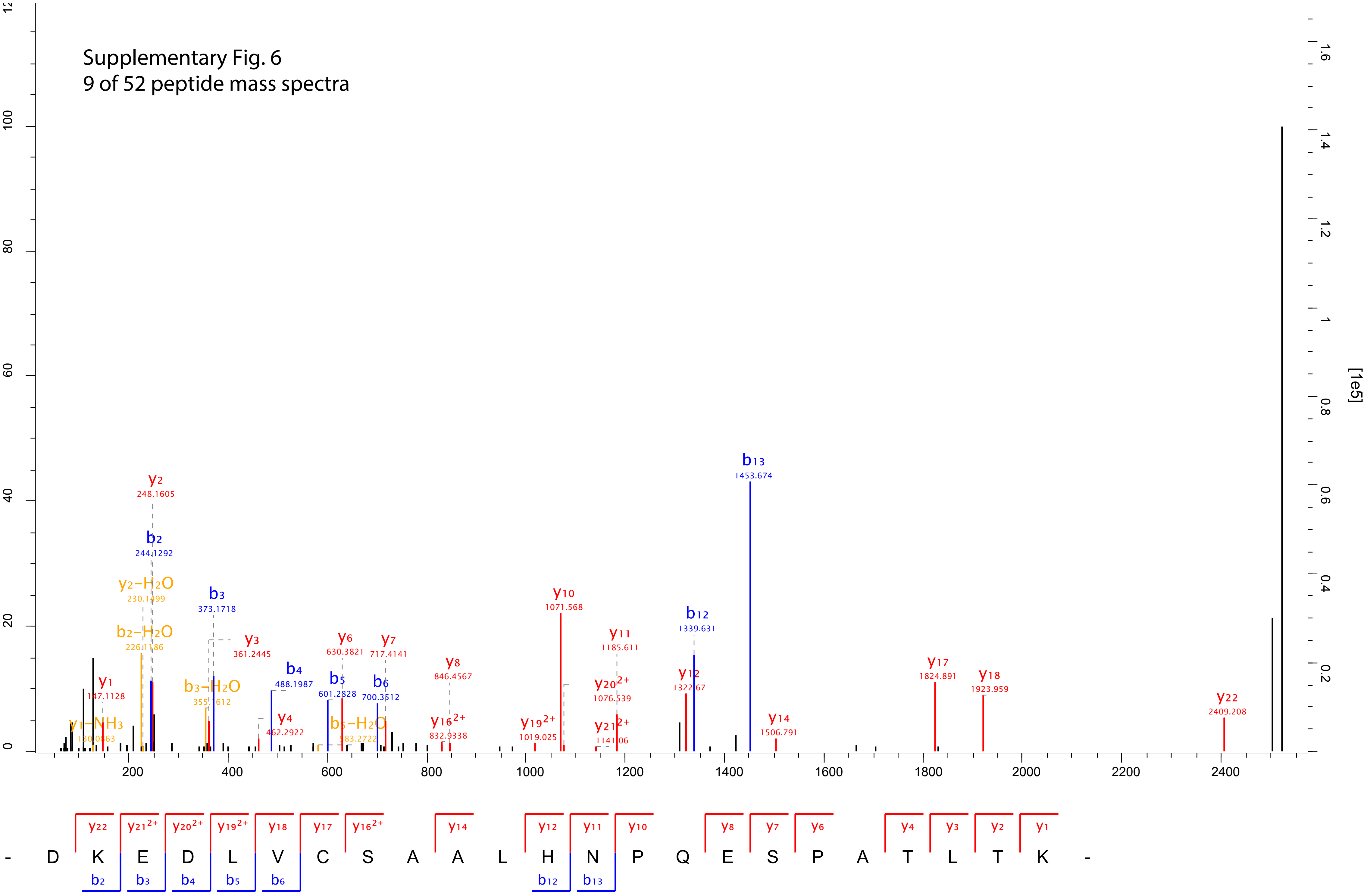

Raw file	Scan	Method	Score	m/z
180221_FS1_Li_DMo_cRNA_Try	21837	FTMS; HCD	58.56	703.01

Supplementary Fig. 6
10 of 52 peptide mass spectra

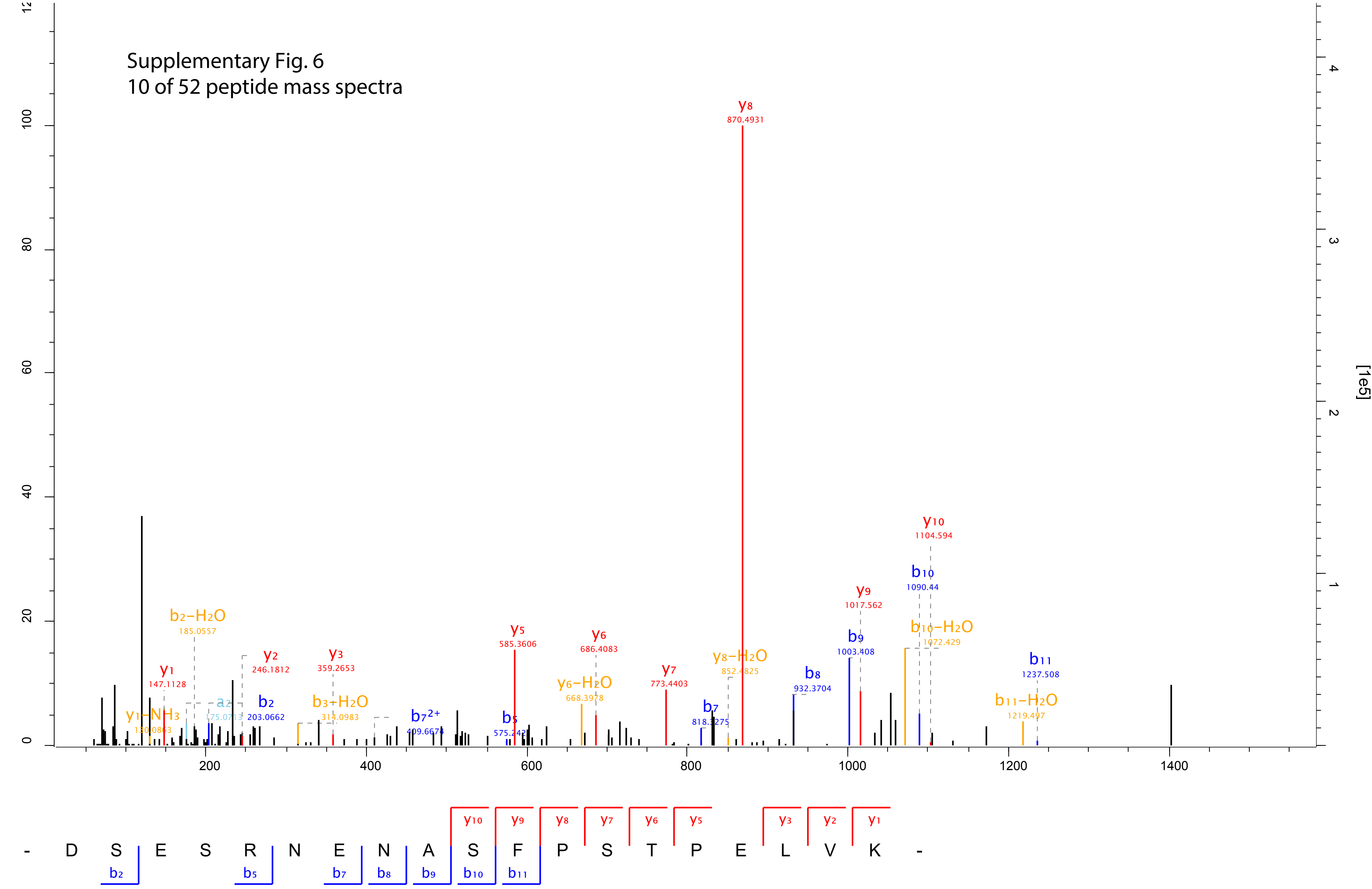

Raw file	Scan	Method	Score	m/z
180221_FS1_Li_DMo_cRNA_Try	12846	FTMS; HCD	74.99	484.91

Supplementary Fig. 6
11 of 52 peptide mass spectra

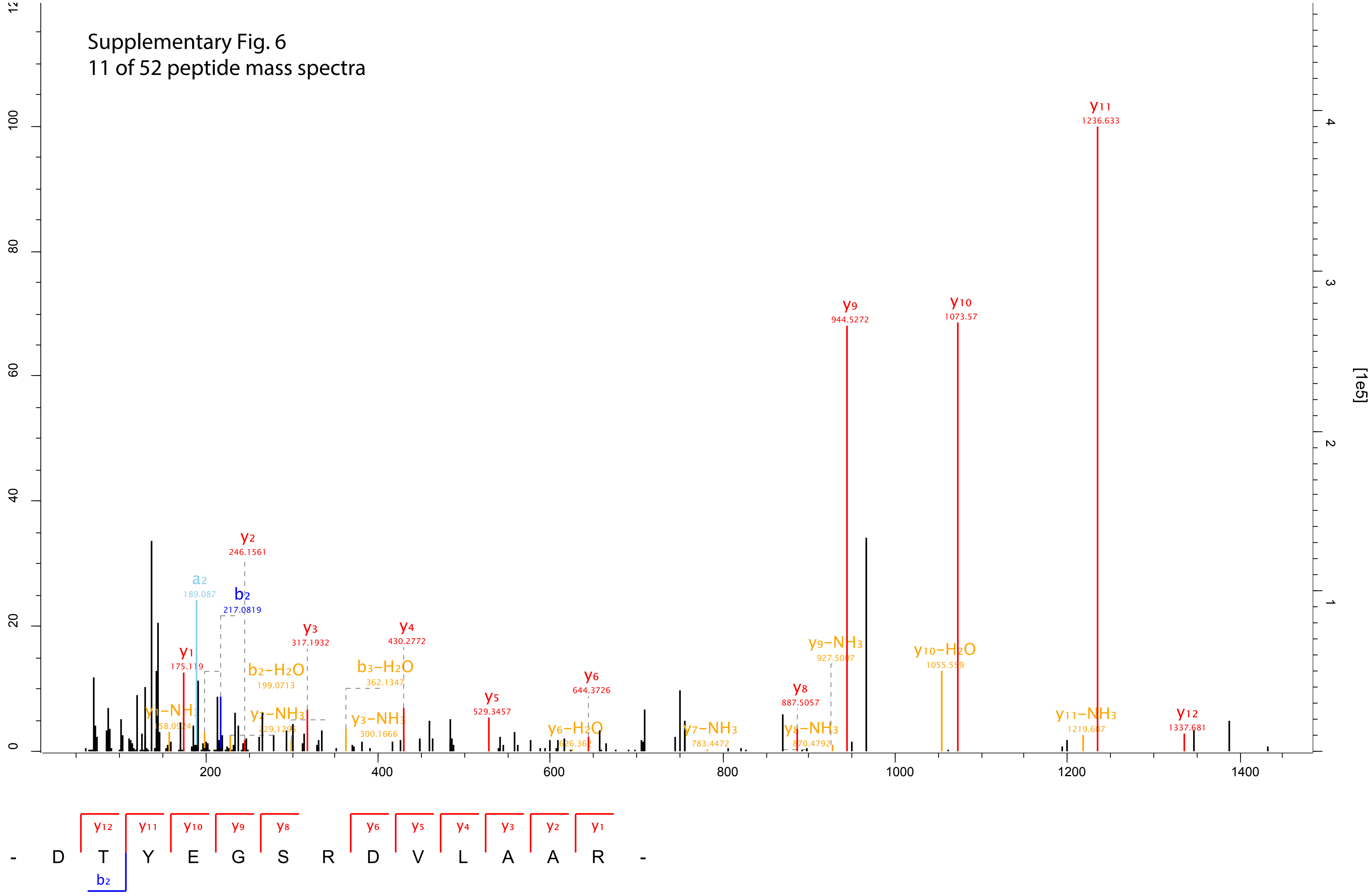

Raw file	Scan	Method	Score	m/z
180221_FS1_Li_DMo_cRNA_Try	5703	FTMS; HCD	126.77	592.73

Supplementary Fig. 6
12 of 52 peptide mass spectra

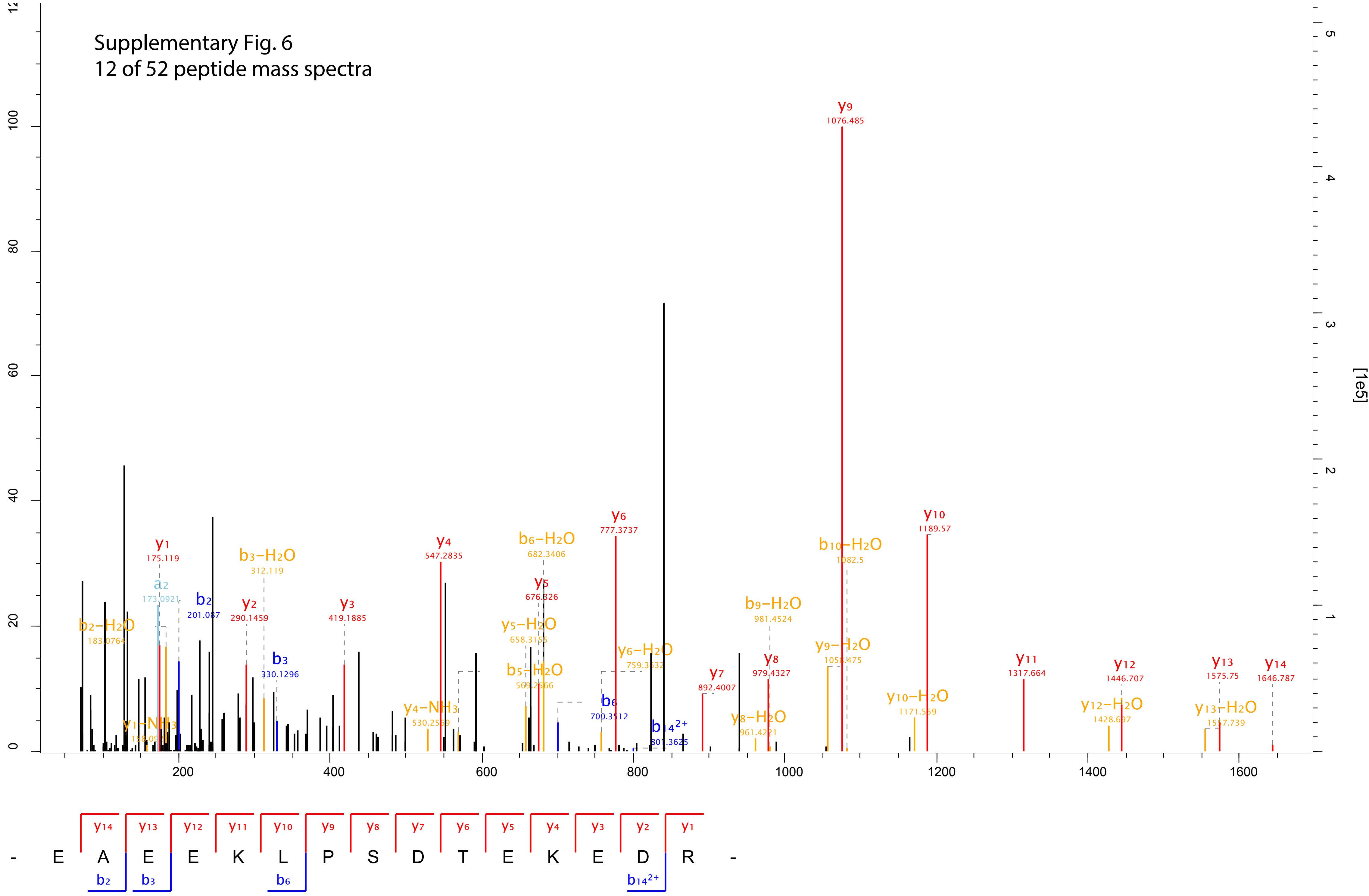

Raw file	Scan	Method	Score	m/z
180221_FS1_Li_DMo_cRNA_F_P	5317	FTMS; HCD	125.71	496.23

Supplementary Fig. 6
13 of 52 peptide mass spectra

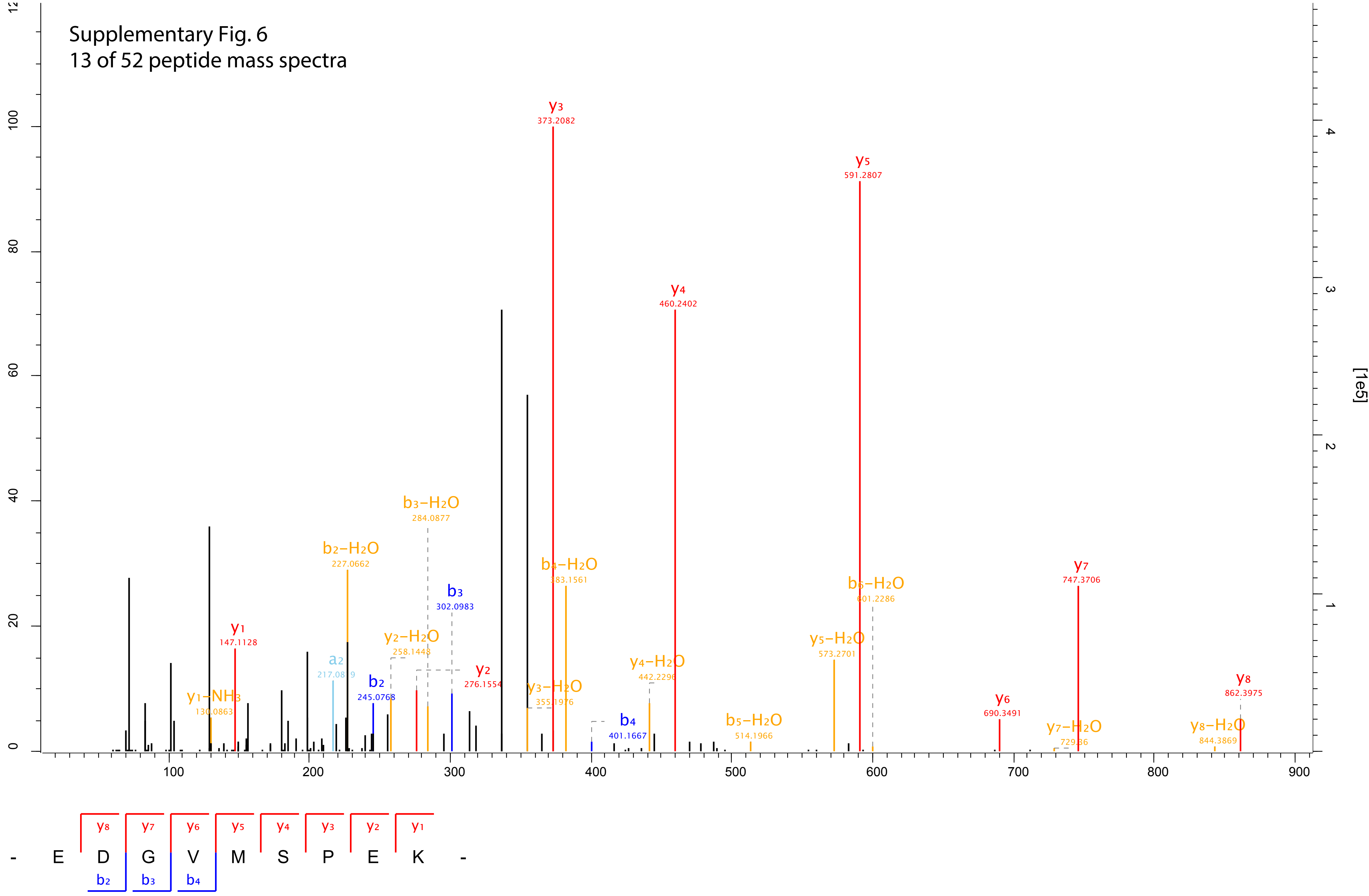

Supplementary Fig. 6
14 of 52 peptide mass spectra

Raw file	Scan	Method	Score	m/z
180221_FS1_Li_DMo_cRNA_Try	37853	FTMS; HCD	38.77	1089.55

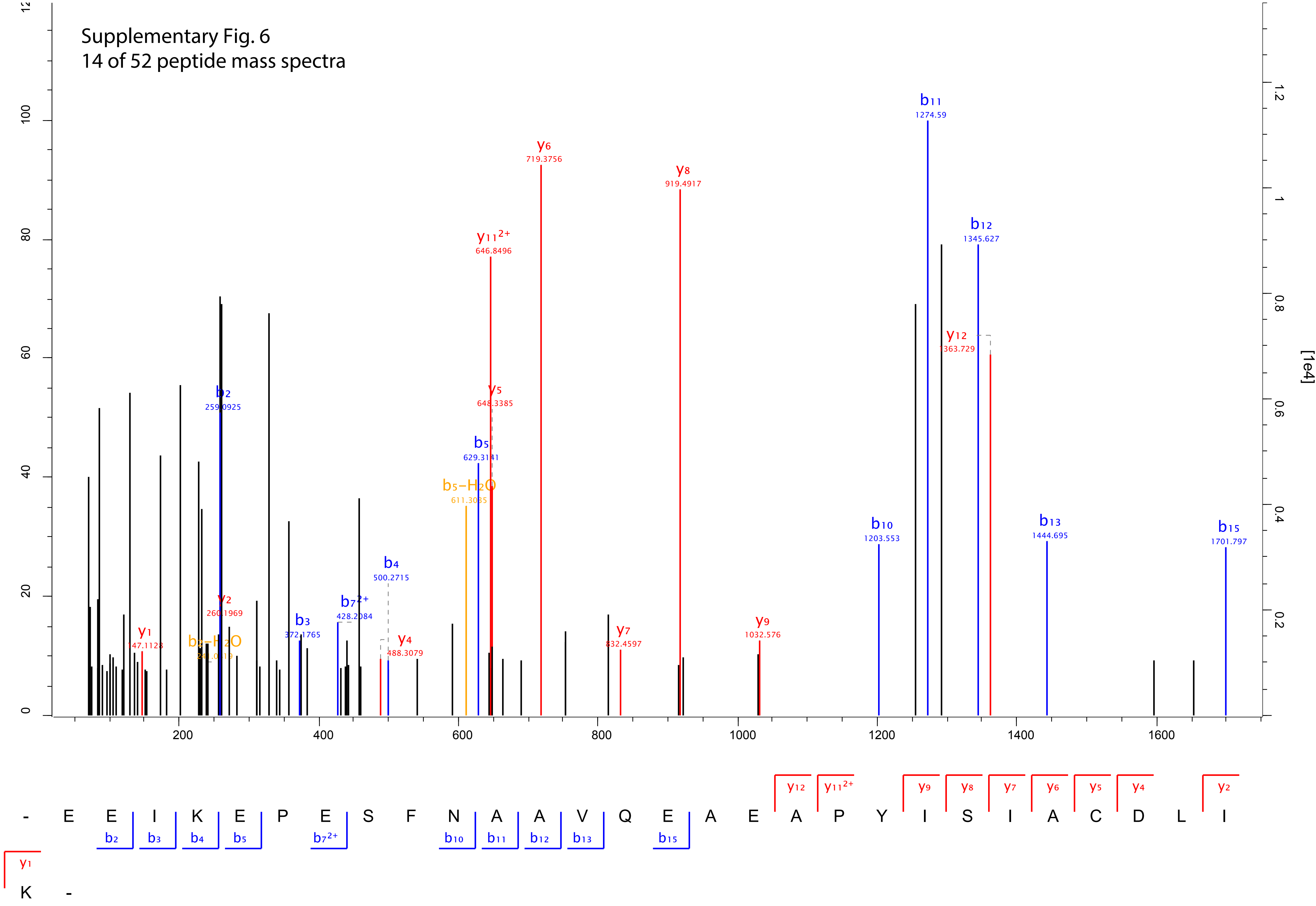

Raw file	Scan	Method	Score	m/z
180221_FS1_Li_DMo_cRNA_F_P	32625	FTMS; HCD	109.07	672.99

Supplementary Fig. 6
15 of 52 peptide mass spectra

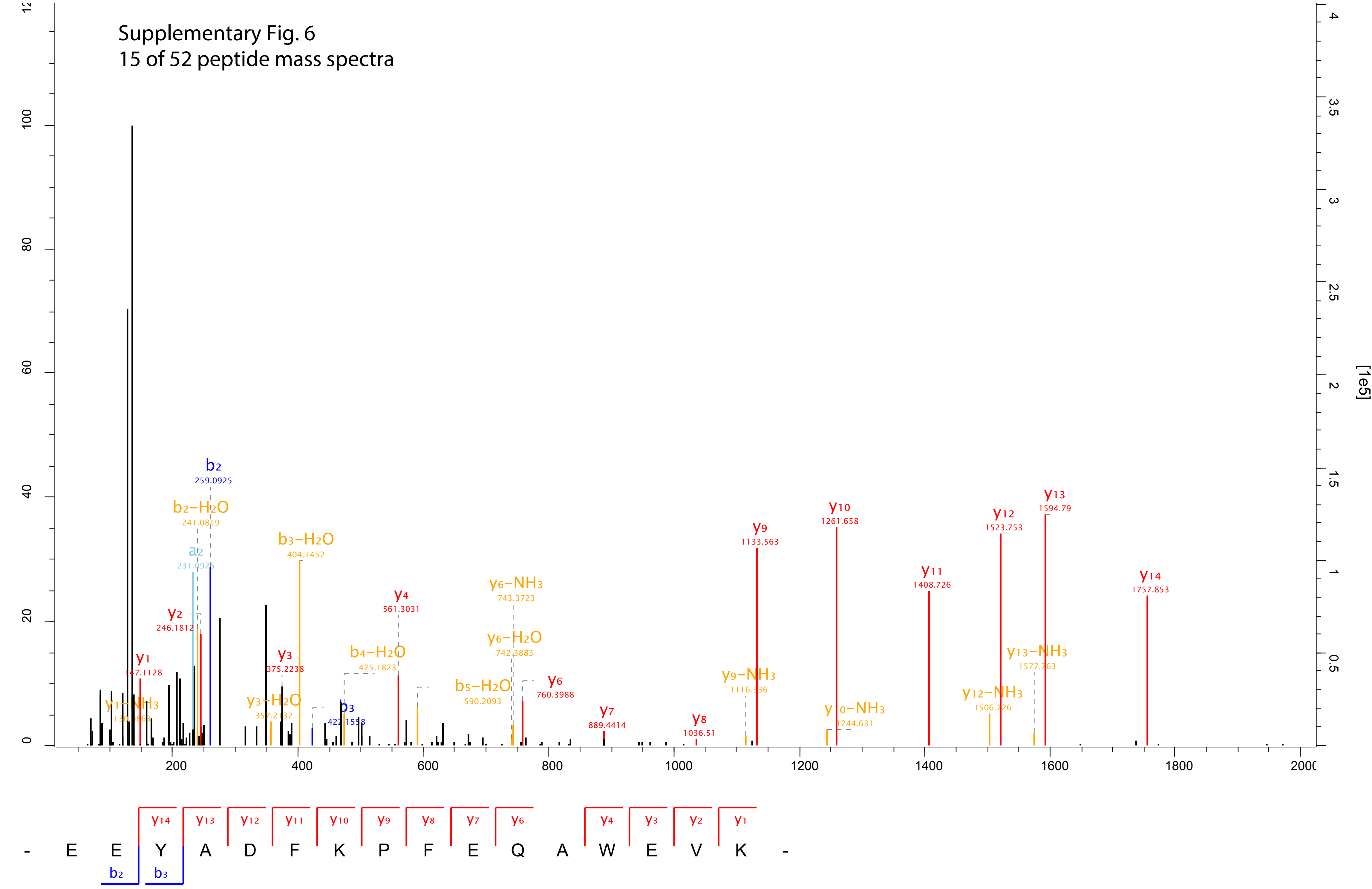

Raw file	Scan	Method	Score	m/z
180221_FS1_Li_DMo_cRNA_Try	32685	FTMS; HCD	146.6	707.08

Supplementary Fig. 6
16 of 52 peptide mass spectra

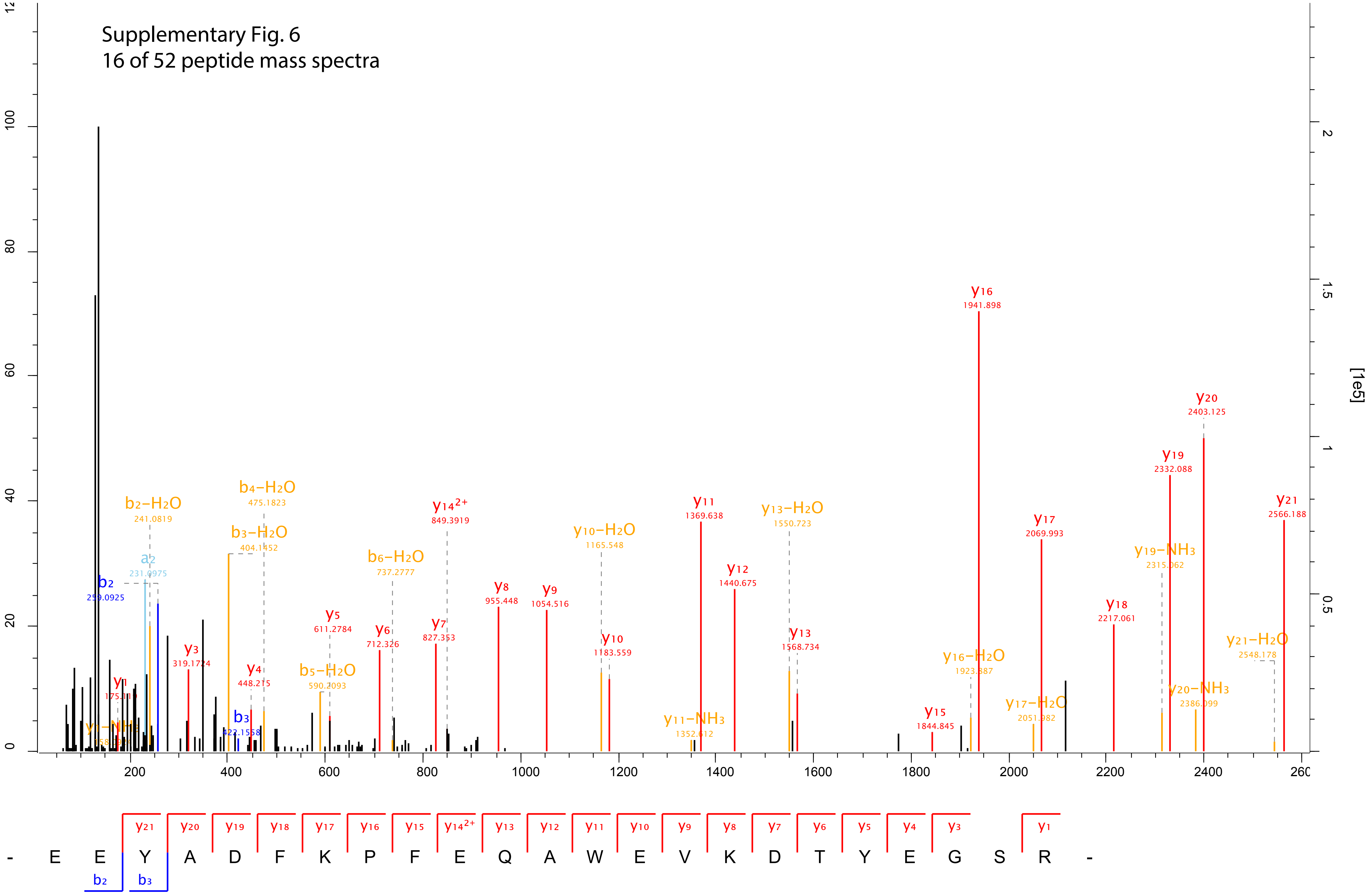

Raw file	Scan	Method	Score	m/z
180221_FS1_Li_DMo_cRNA_Try	35935	FTMS; HCD	168.06	950.47

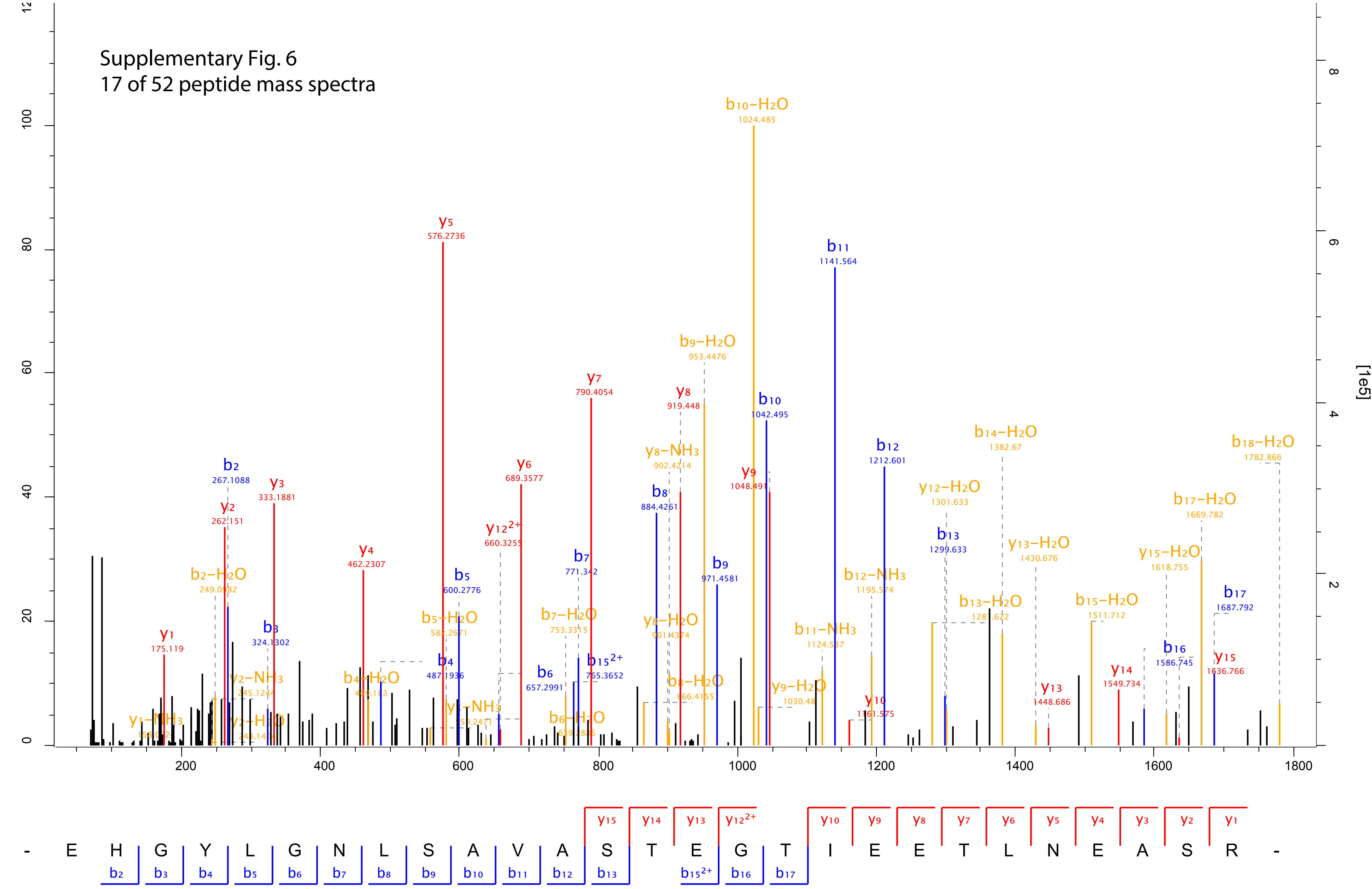

Supplementary Fig. 6
17 of 52 peptide mass spectra

Raw file	Scan	Method	Score	m/z
180221_FS1_Li_DMo_cRNA_Try	40573	FTMS; HCD	47.39	1285.99

Supplementary Fig. 6
18 of 52 peptide mass spectra

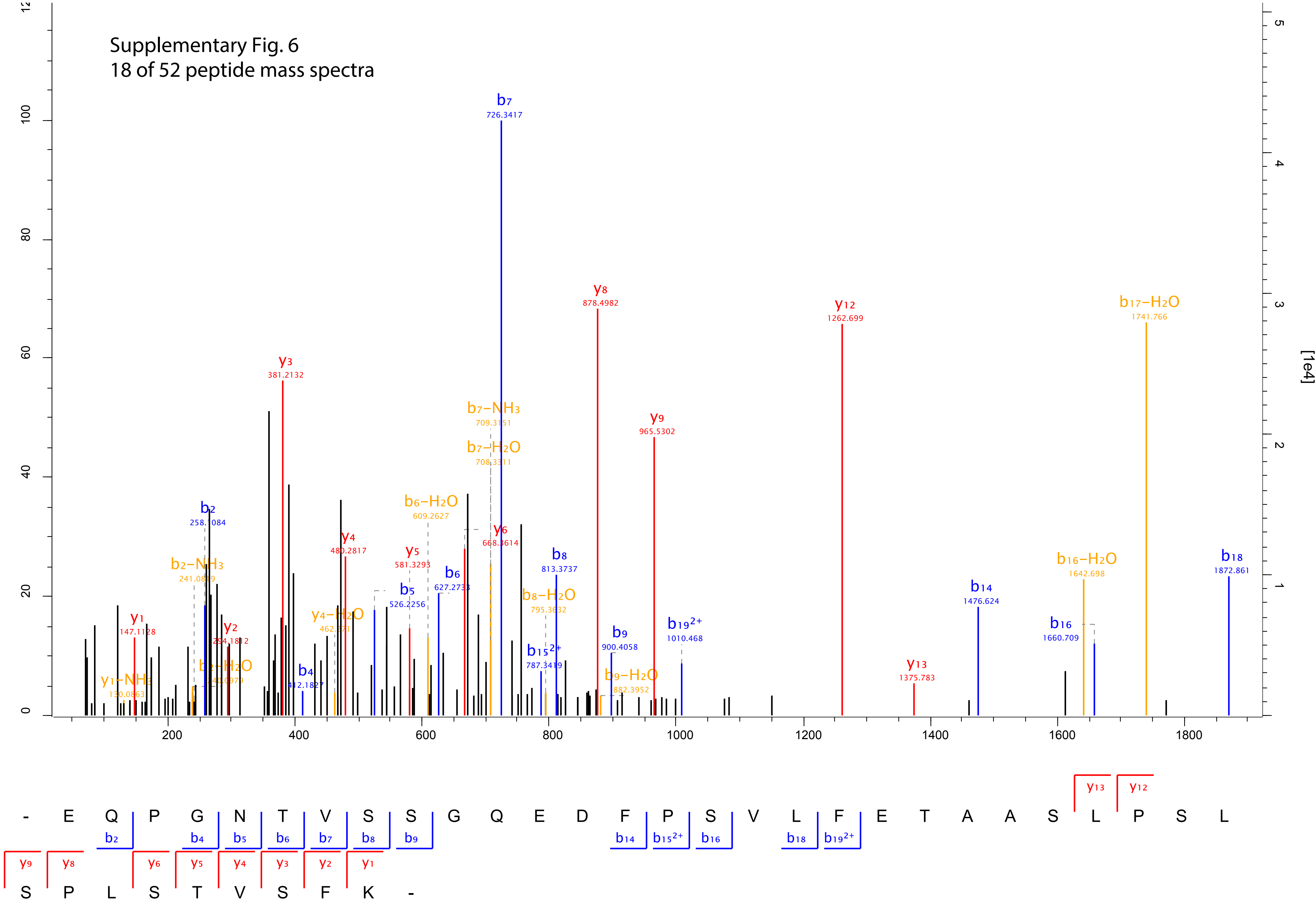

Raw file	Scan	Method	Score	m/z
180221_FS1_Li_DMo_cRNA_Try	53267	FTMS; HCD	143.57	795.03

Supplementary Fig. 6
19 of 52 peptide mass spectra

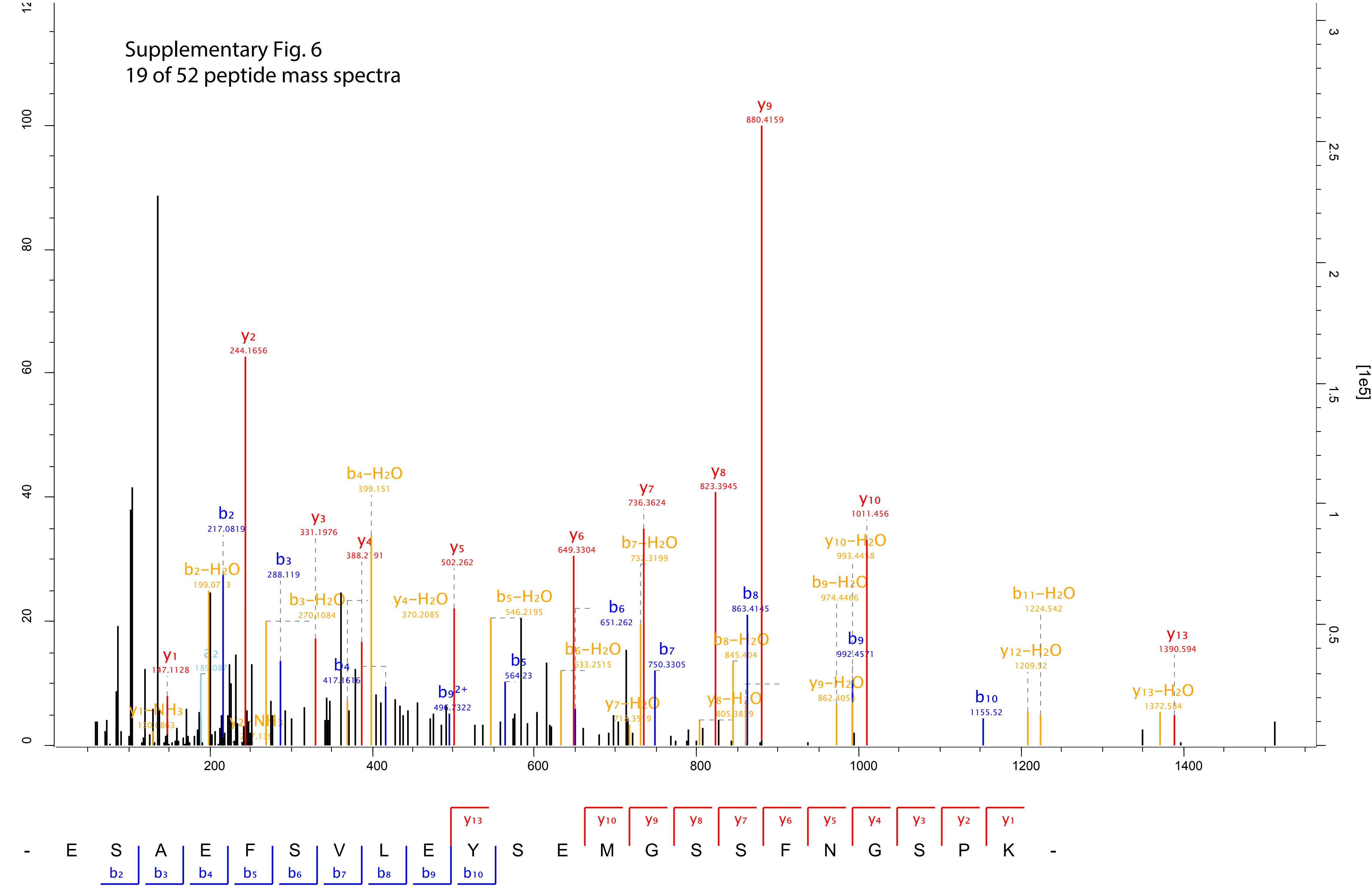

Raw file	Scan	Method	Score	m/z
180221_FS1_Li_DMo_cRNA_Try	38959	FTMS; HCD	111.38	892.77

Supplementary Fig. 6
20 of 52 peptide mass spectra

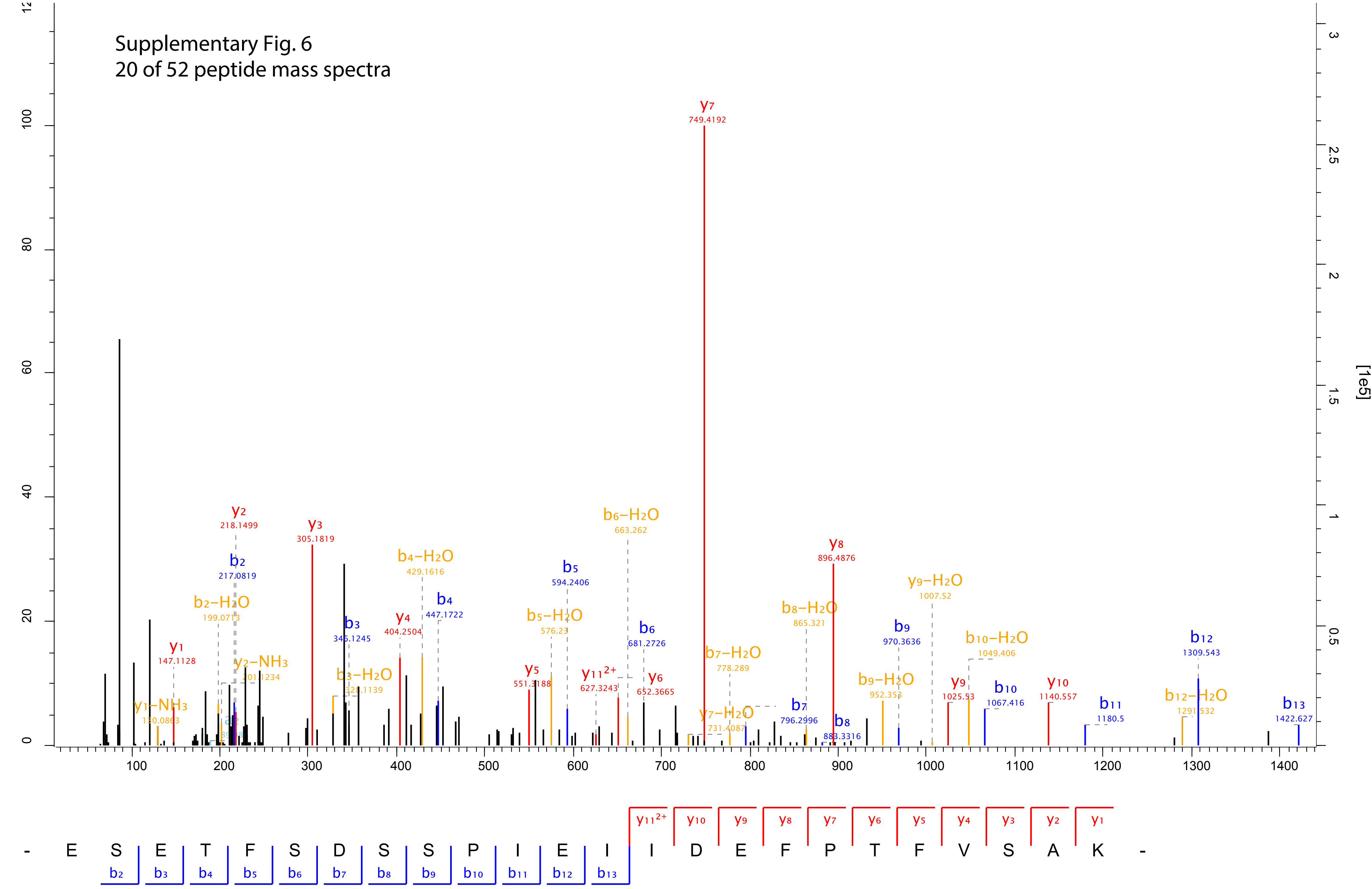

Raw file	Scan	Method	Score	m/z
180221_FS1_Li_DMo_cRNA_F_P	17476	FTMS; HCD	144.51	529.94

Supplementary Fig. 6
21 of 52 peptide mass spectra

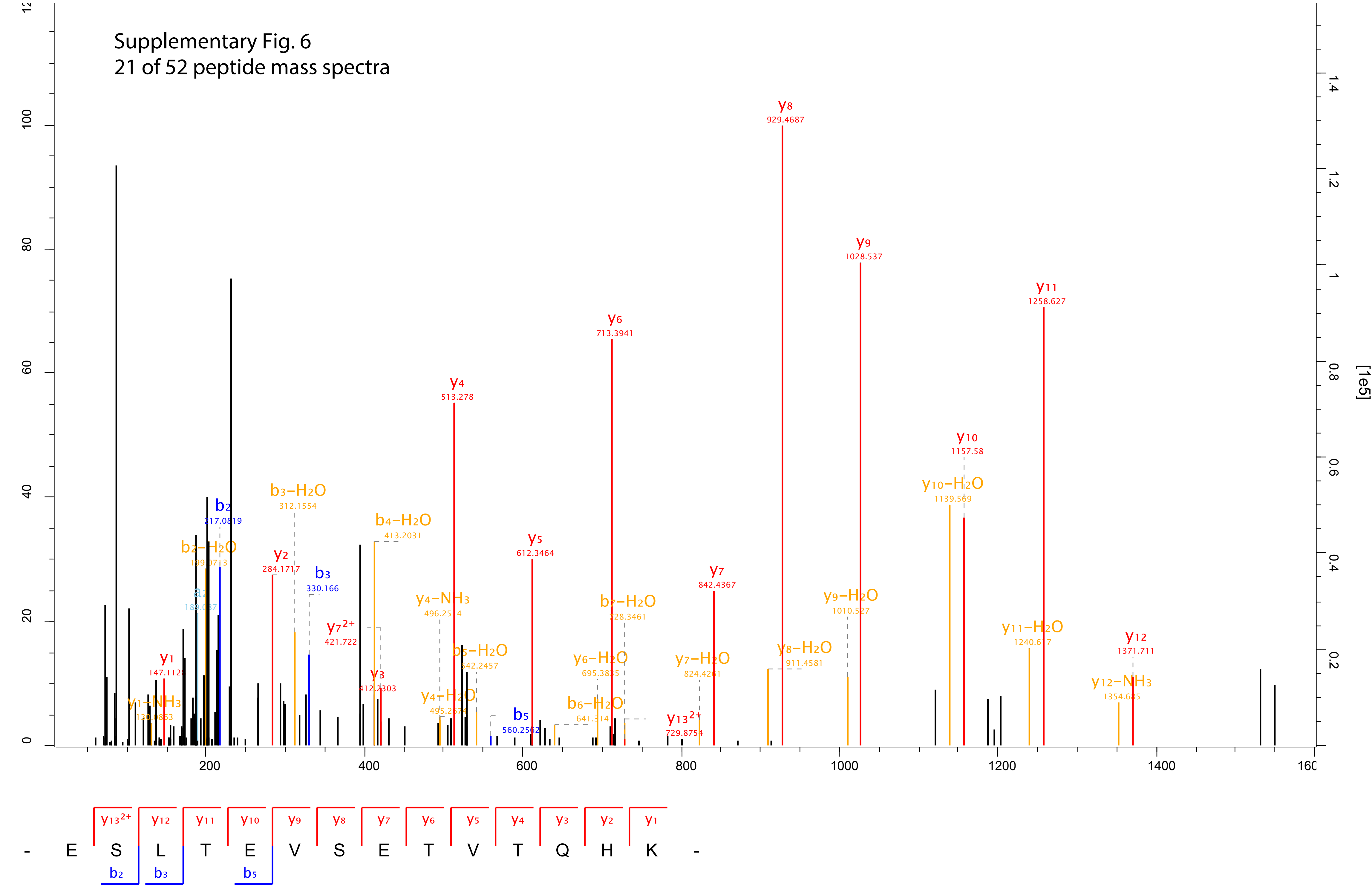

Raw file	Scan	Method	Score	m/z
180221_FS1_Li_DMo_cRNA_F_P	52319	FTMS; HCD	137.64	917.11

Supplementary Fig. 6
22 of 52 peptide mass spectra

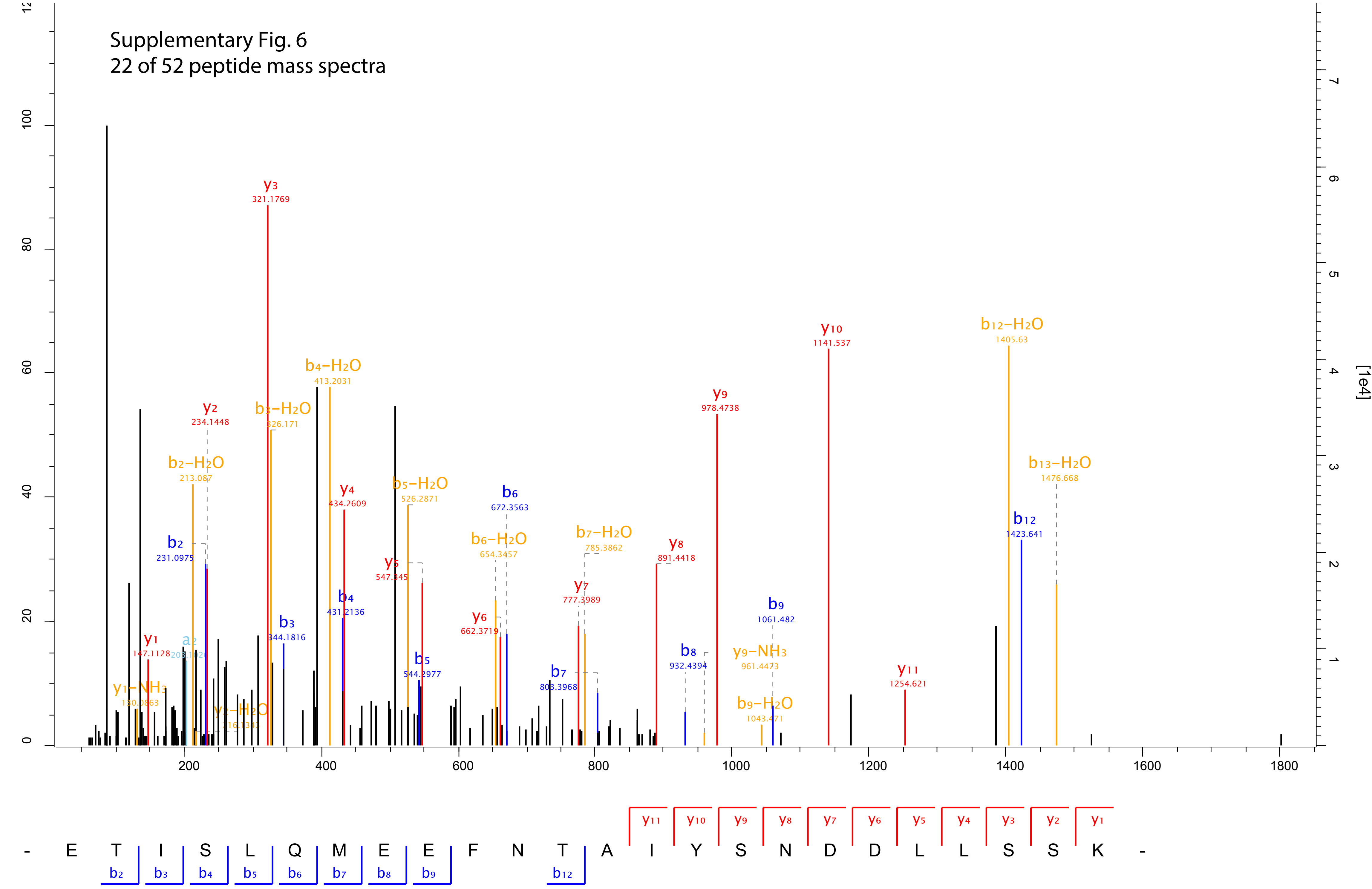

Raw file	Scan	Method	Score	m/z
180221_FS1_Li_DMo_cRNA_Try	53274	FTMS; HCD	135.8	1041.17

Supplementary Fig. 6
23 of 52 peptide mass spectra

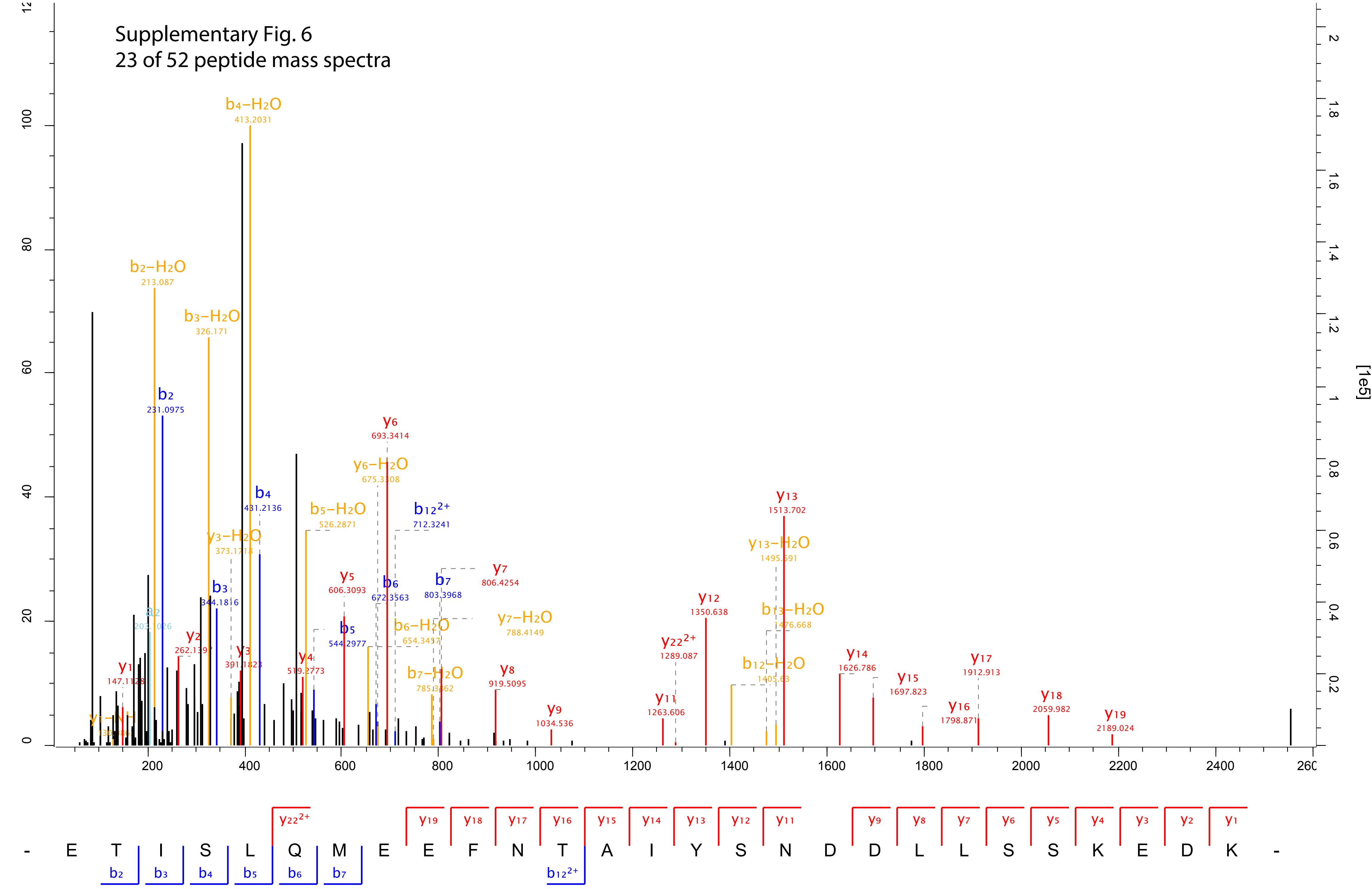

Raw file	Scan	Method	Score	m/z
180221_FS1_Li_DMo_cRNA_F_P	11919	FTMS; HCD	90.14	599.29

Supplementary Fig. 6
24 of 52 peptide mass spectra

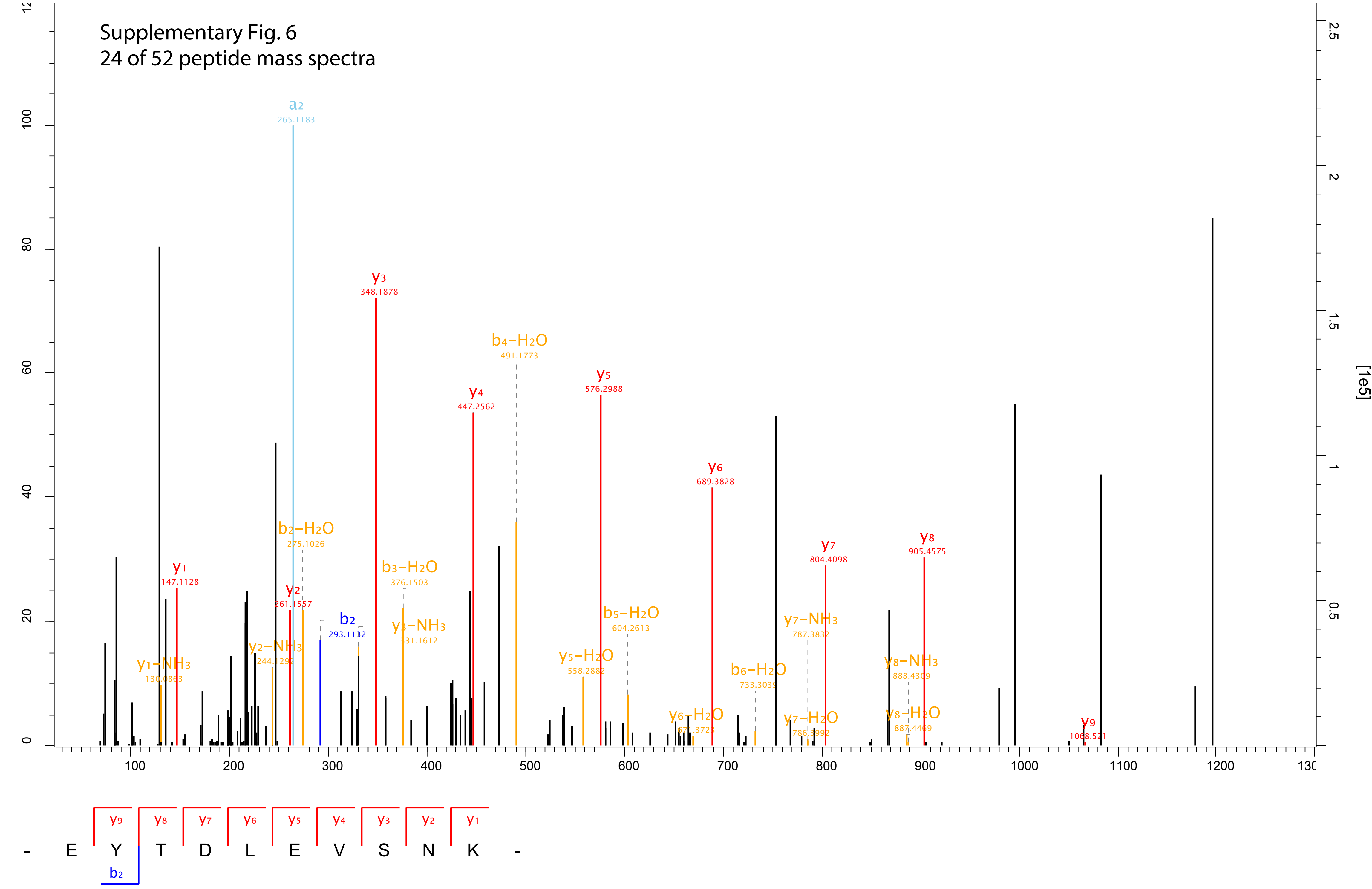

Raw file	Scan	Method	Score	m/z
180221_FS1_Li_DMo_cRNA_F_P	9585	FTMS; HCD	75.74	589.79

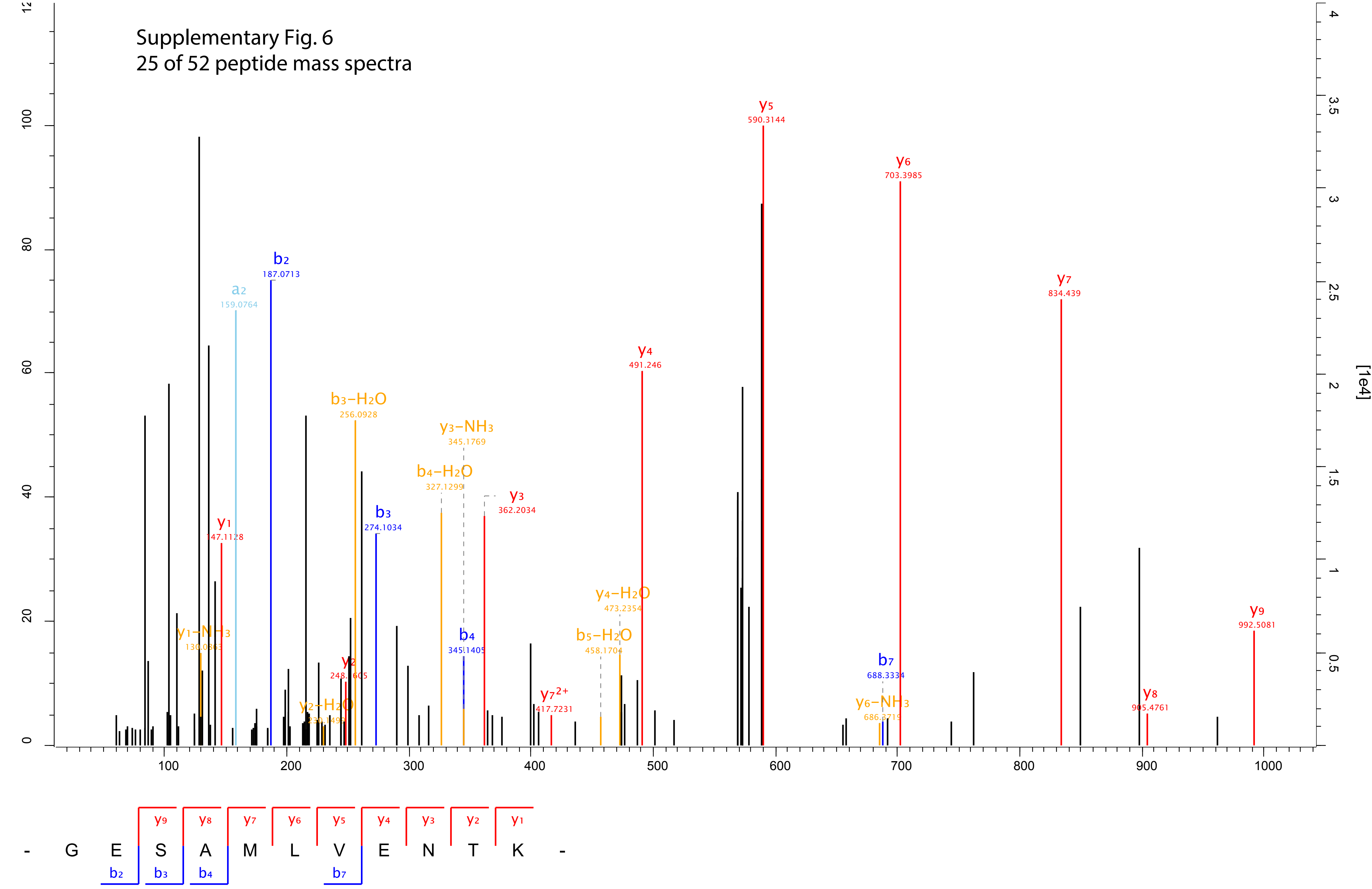

Raw file	Scan	Method	Score	m/z
180221_FS1_Li_DMo_cRNA_F_P	51043	FTMS; HCD	244.17	635.67

Supplementary Fig. 6
26 of 52 peptide mass spectra

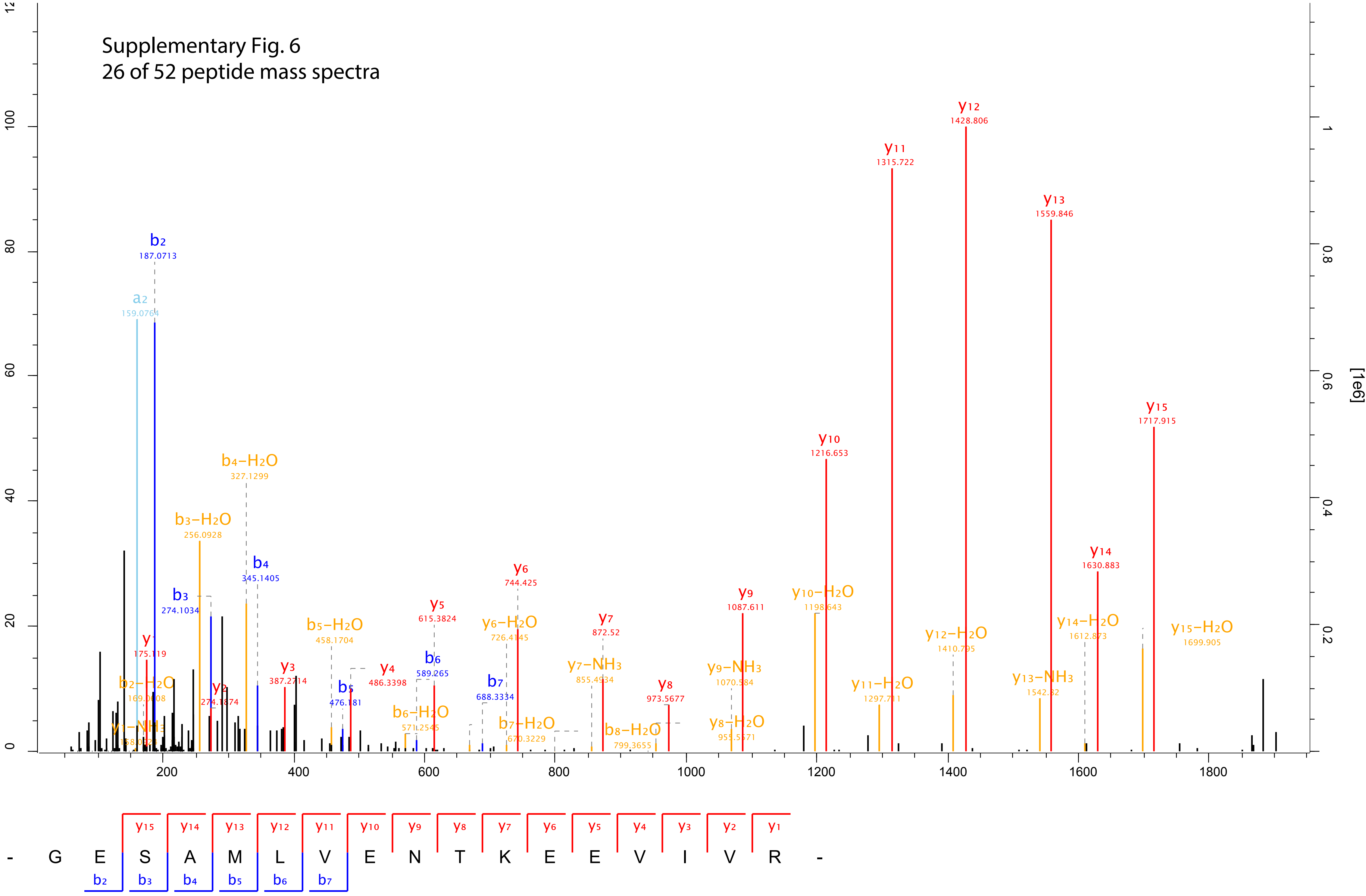

Raw file	Scan	Method	Score	m/z
180221_FS1_Li_DMo_cRNA_Try	4379	FTMS; HCD	92.78	448.6

Supplementary Fig. 6
27 of 52 peptide mass spectra

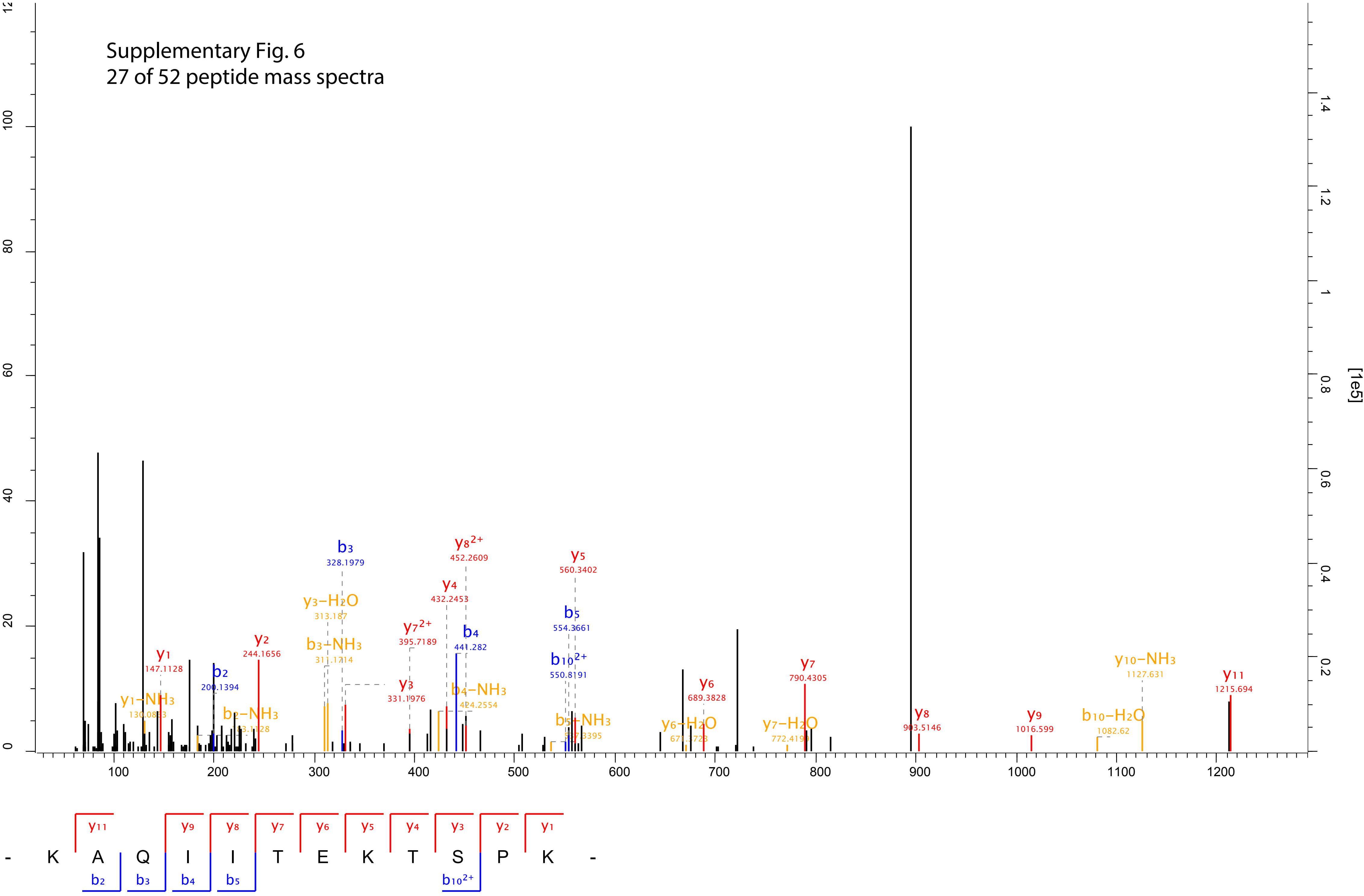

Raw file	Scan	Method	Score	m/z
180221_FS1_Li_DMo_cRNA_Try	53024	FTMS; HCD	87.28	813.09

Supplementary Fig. 6
28 of 52 peptide mass spectra

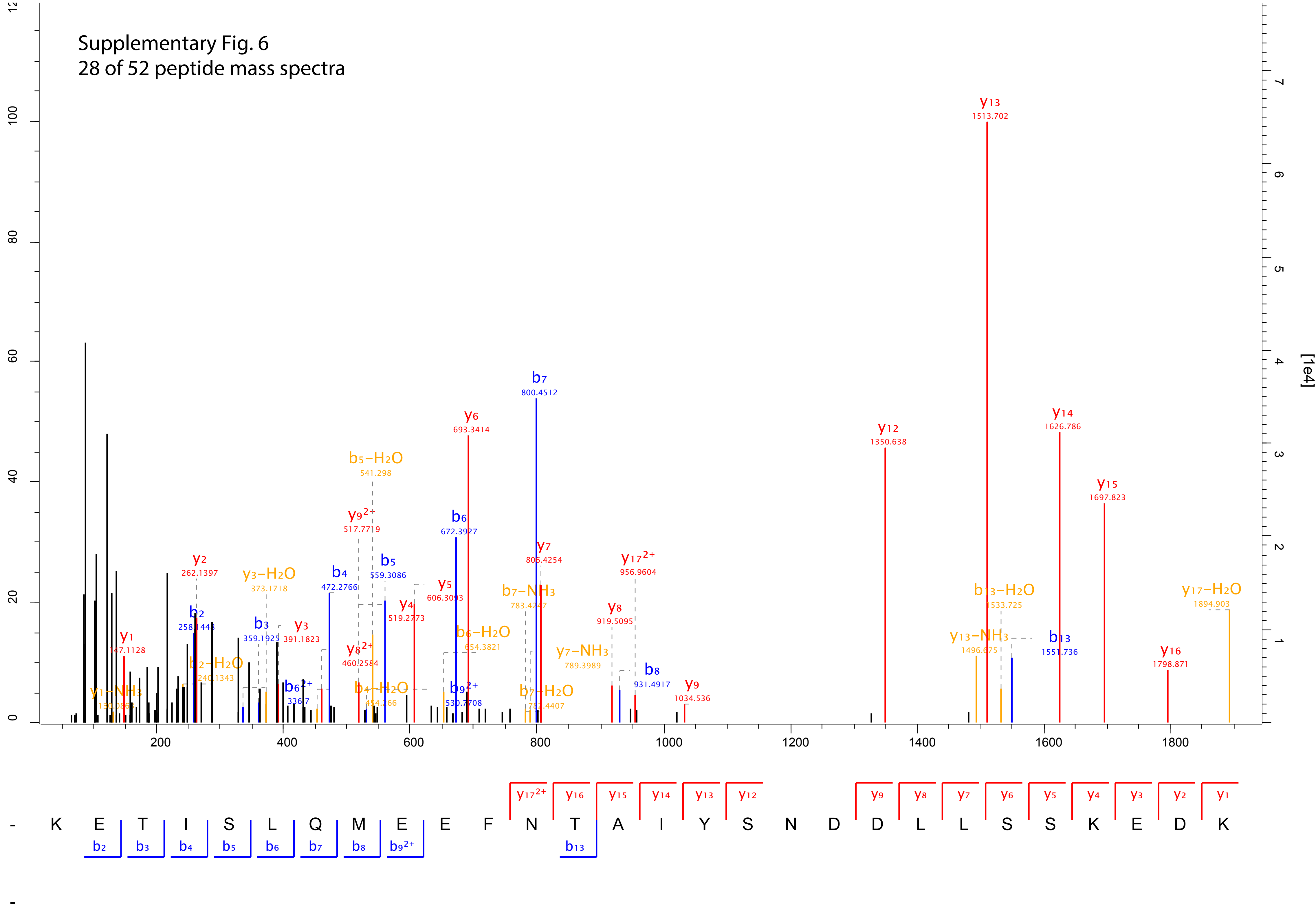

Raw file	Scan	Method	Score	m/z
180221_FS1_Li_DMo_cRNA_F_P	32751	FTMS; HCD	106.93	672.34

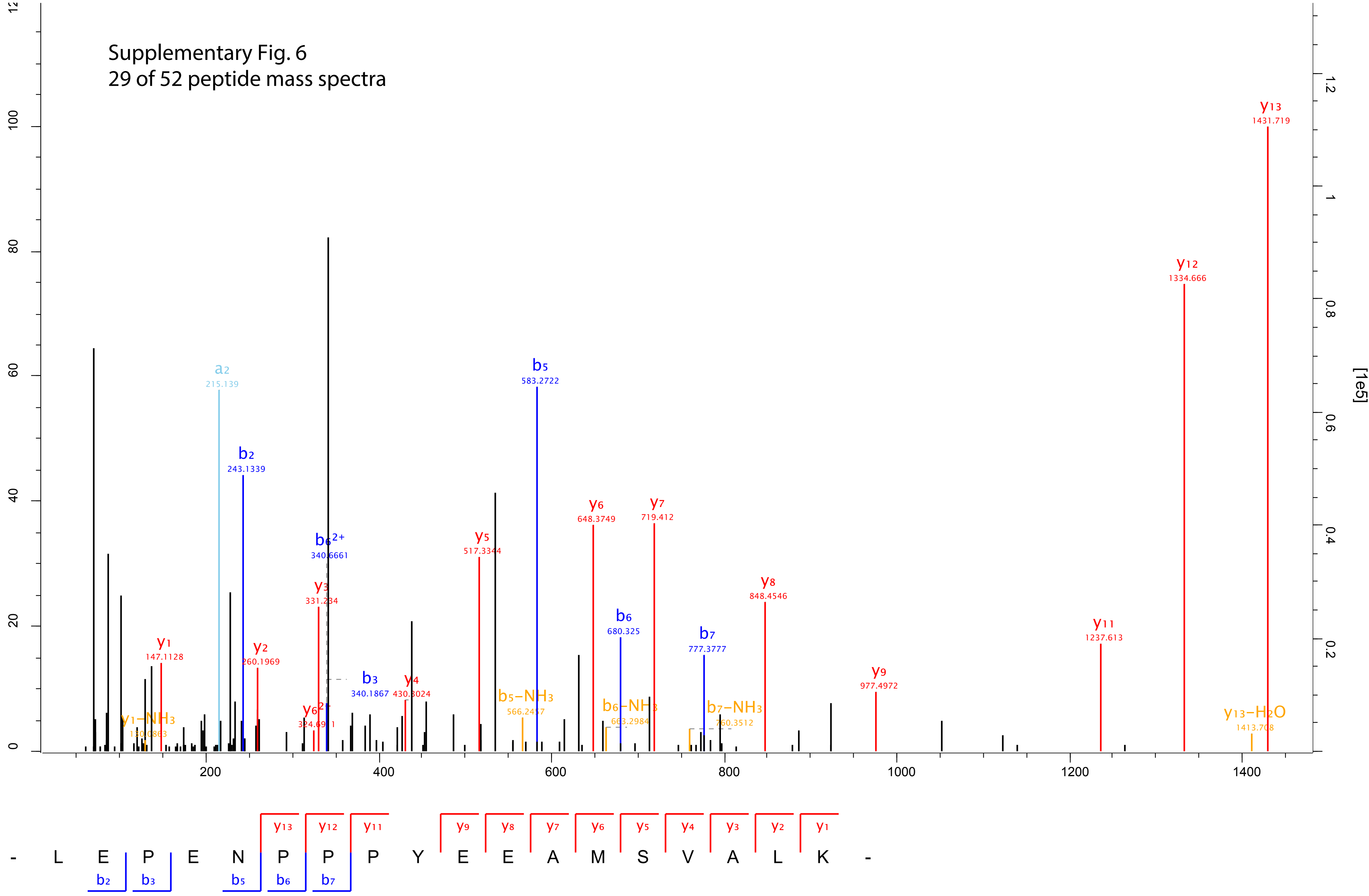

Raw file	Scan	Method	Score	m/z	Gene names
180221_FS1_Li_DMo_cRNA_Try	3175	FTMS; HCD	102.52	397.2	RTN4

Supplementary Fig. 6
30 of 52 peptide mass spectra

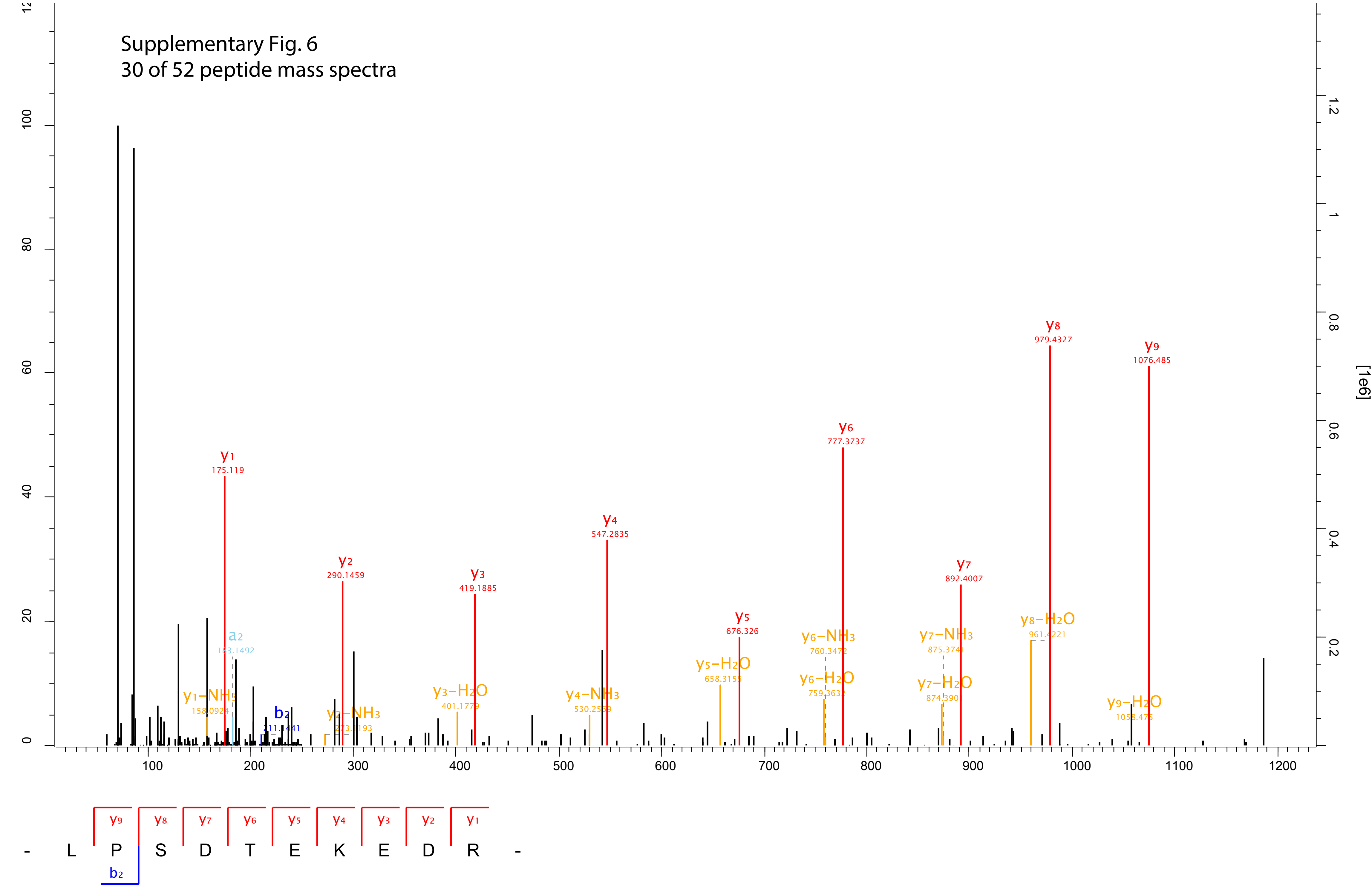

Raw file	Scan	Method	Score	m/z
180221_FS1_Li_DMo_cRNA_Try	25262	FTMS; HCD	93.5	950.46

Supplementary Fig. 6
31 of 52 peptide mass spectra

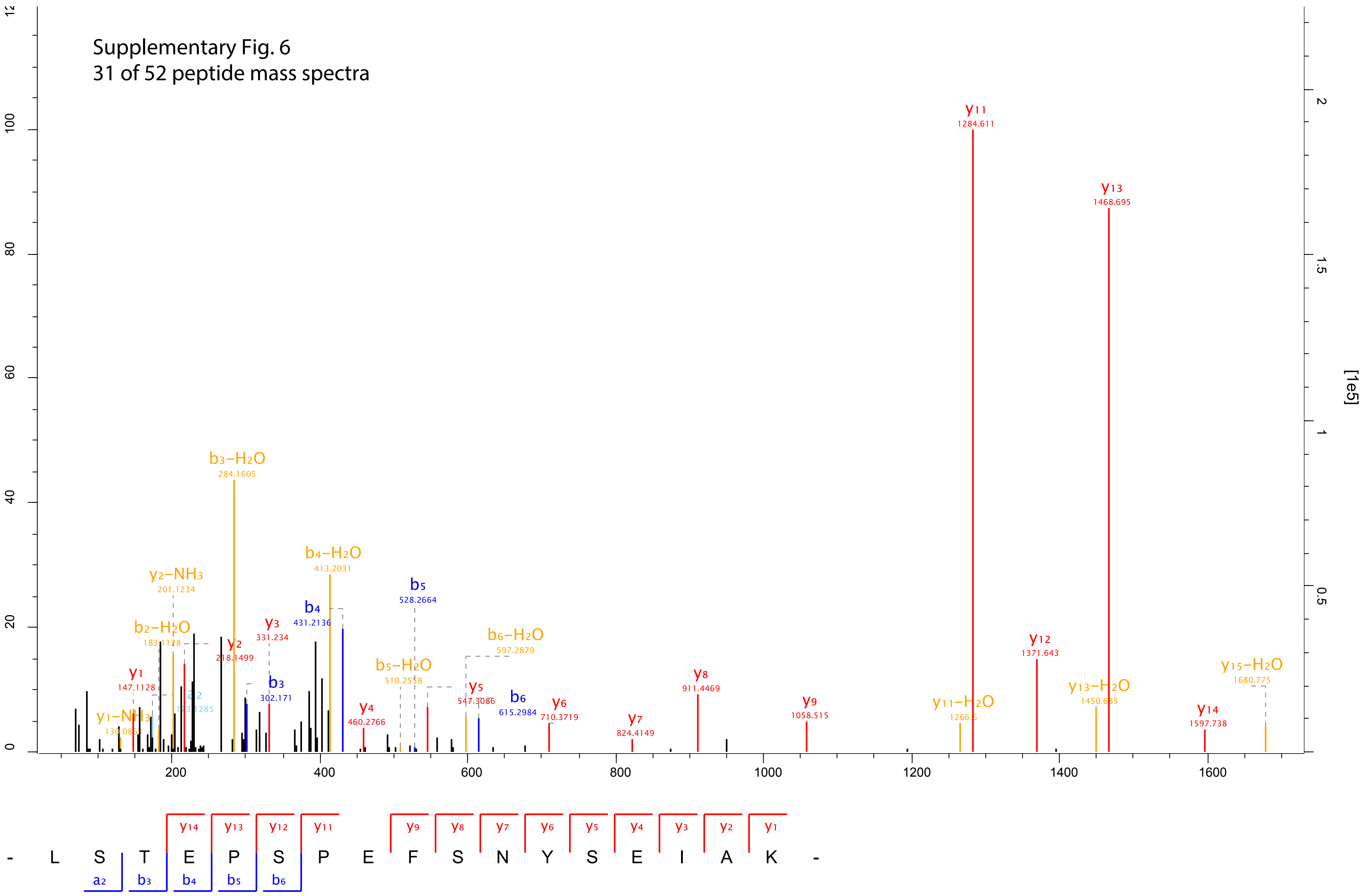

Raw file	Scan	Method	Score	m/z
180221_FS1_Li_DMo_cRNA_Try	29299	FTMS; HCD	109.65	768.72

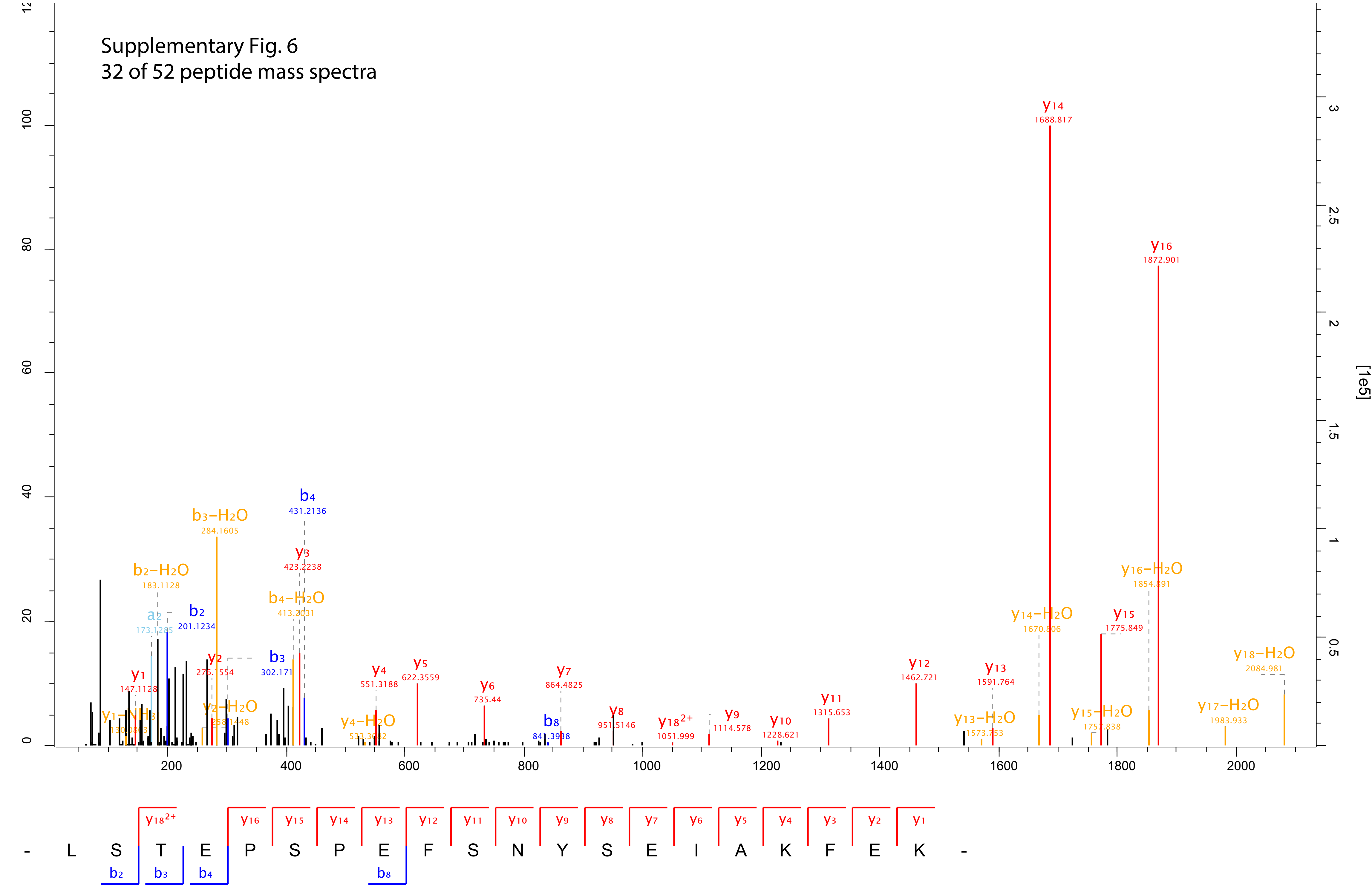

Raw file	Scan	Method	Score	m/z
180221_FS1_Li_DMo_cRNA_Try	37772	FTMS; HCD	89.3	979.15

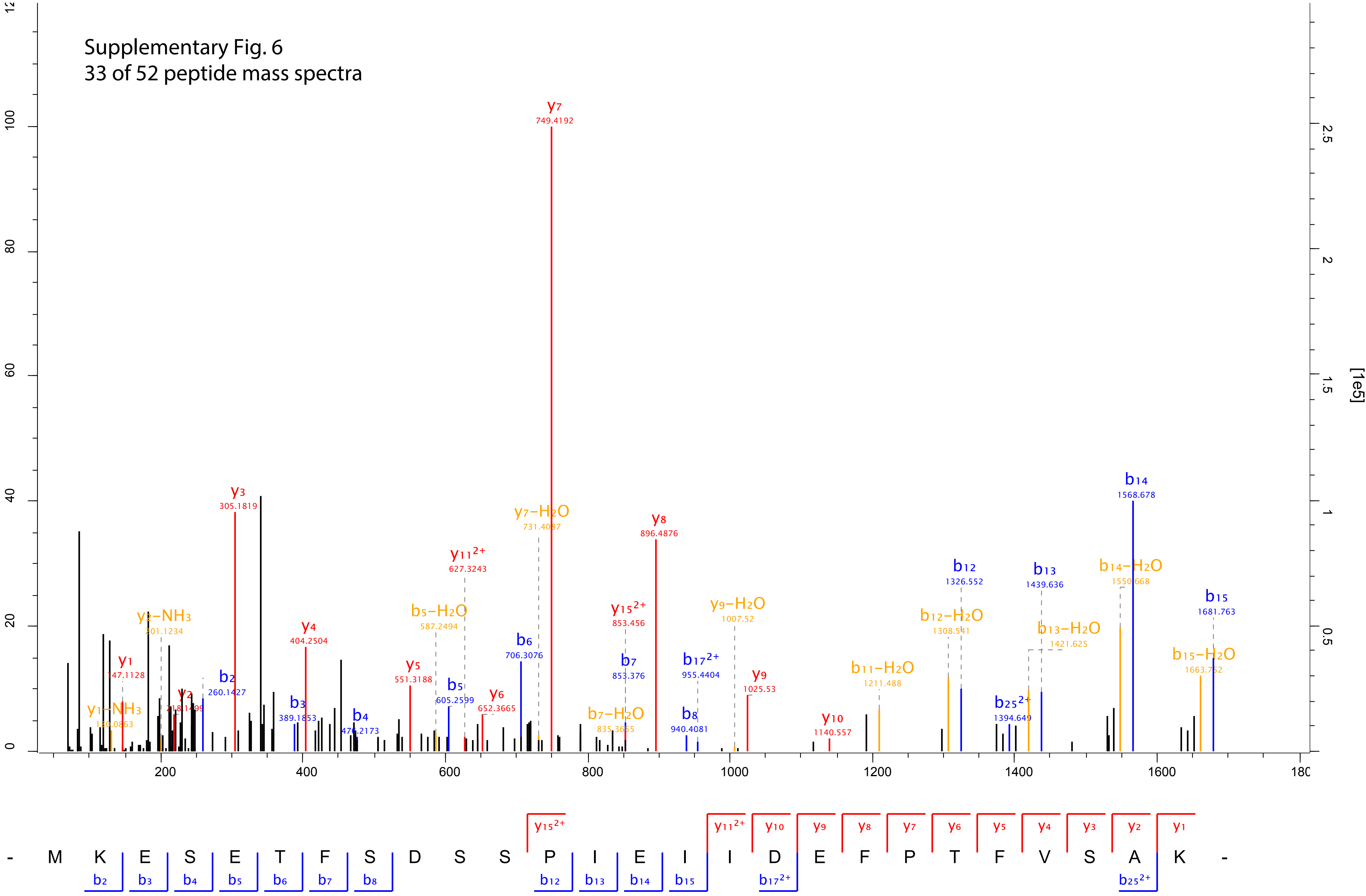

Supplementary Fig. 6
33 of 52 peptide mass spectra

Raw file	Scan	Method	Score	m/z
180221_FS1_Li_DMo_cRNA_Try	36831	FTMS; HCD	35.05	870.42

Supplementary Fig. 6
34 of 52 peptide mass spectra

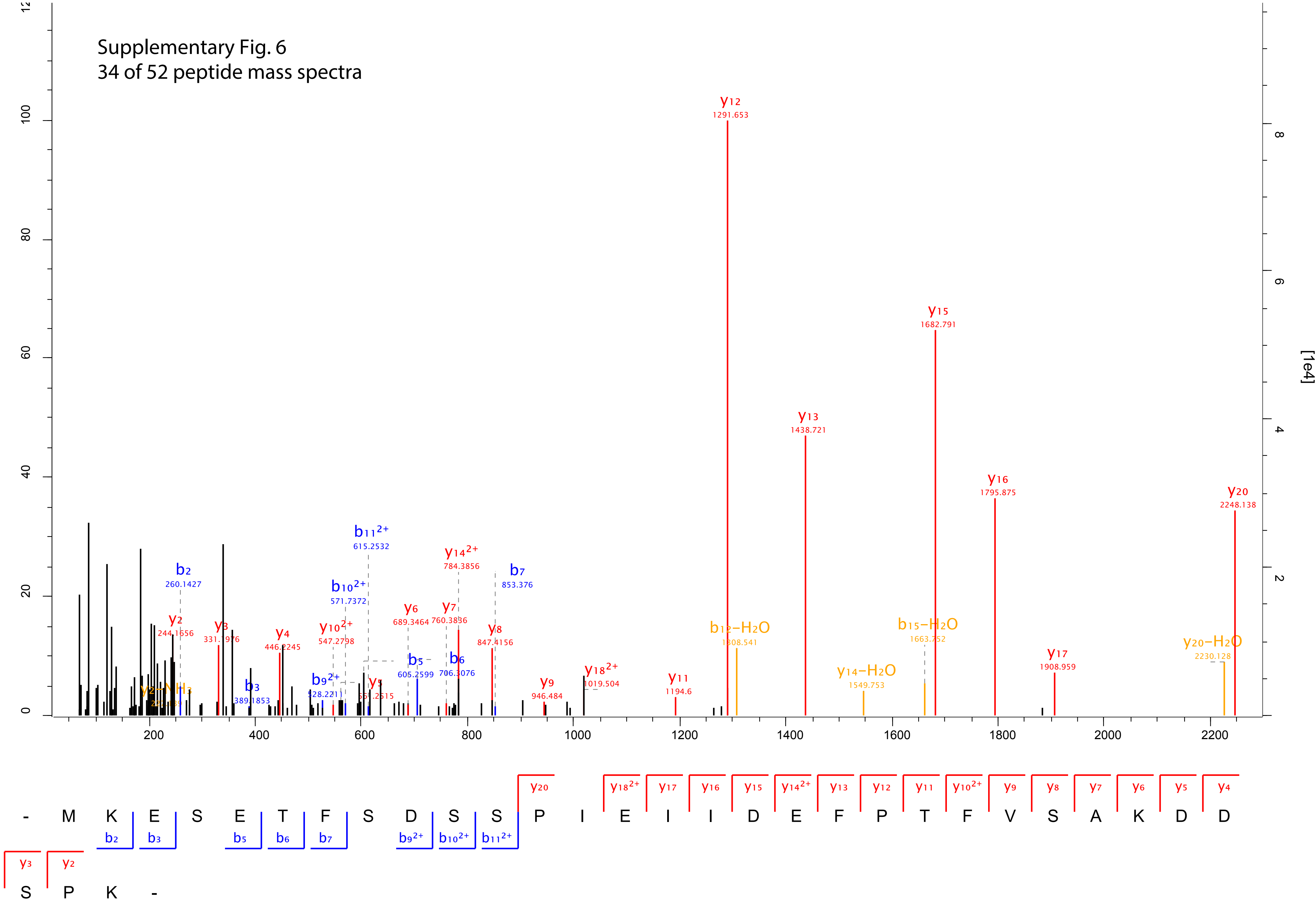

Raw file	Scan	Method	Score	m/z
180221_FS1_Li_DMo_cRNA_F_P	13238	FTMS; HCD	101.28	429.75

Supplementary Fig. 6
35 of 52 peptide mass spectra

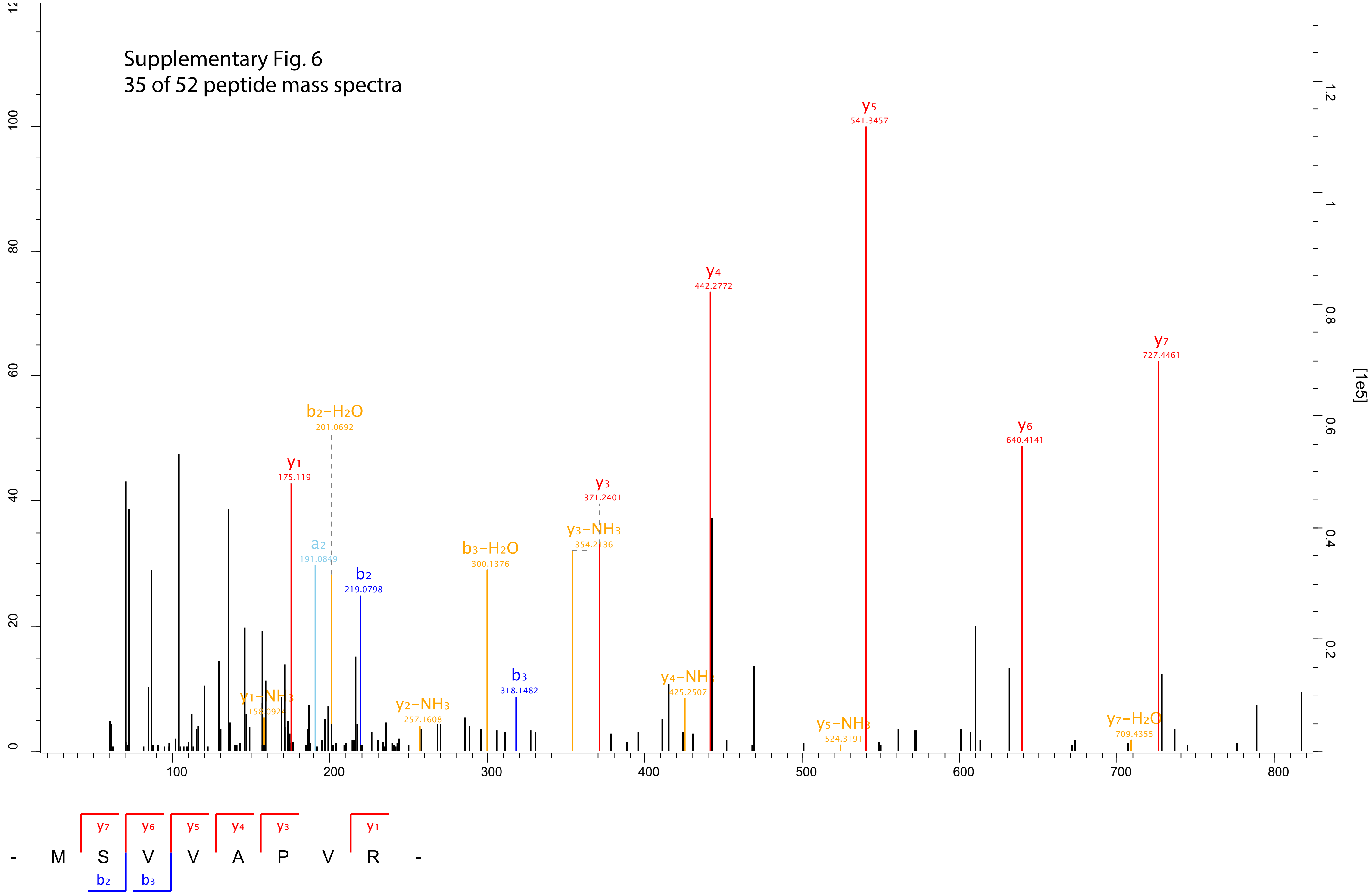

Raw file	Scan	Method	Score	m/z
180221_FS1_Li_DMo_cRNA_Try	34775	FTMS; HCD	135.76	714.86

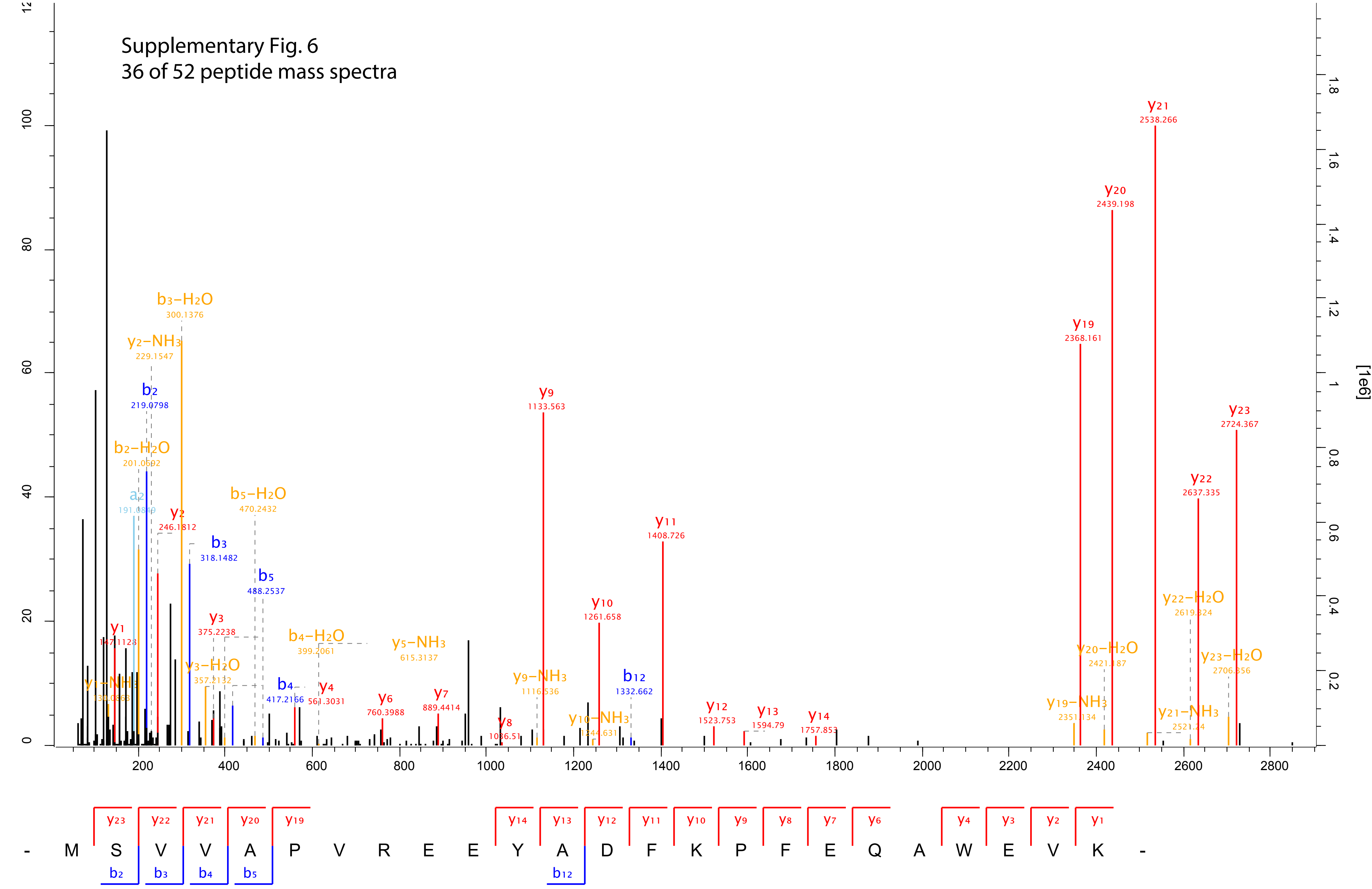

Raw file	Scan	Method	Score	m/z
180221_FS1_Li_DMo_cRNA_Try	33697	FTMS; HCD	139.72	737.16

Supplementary Fig. 6
37 of 52 peptide mass spectra

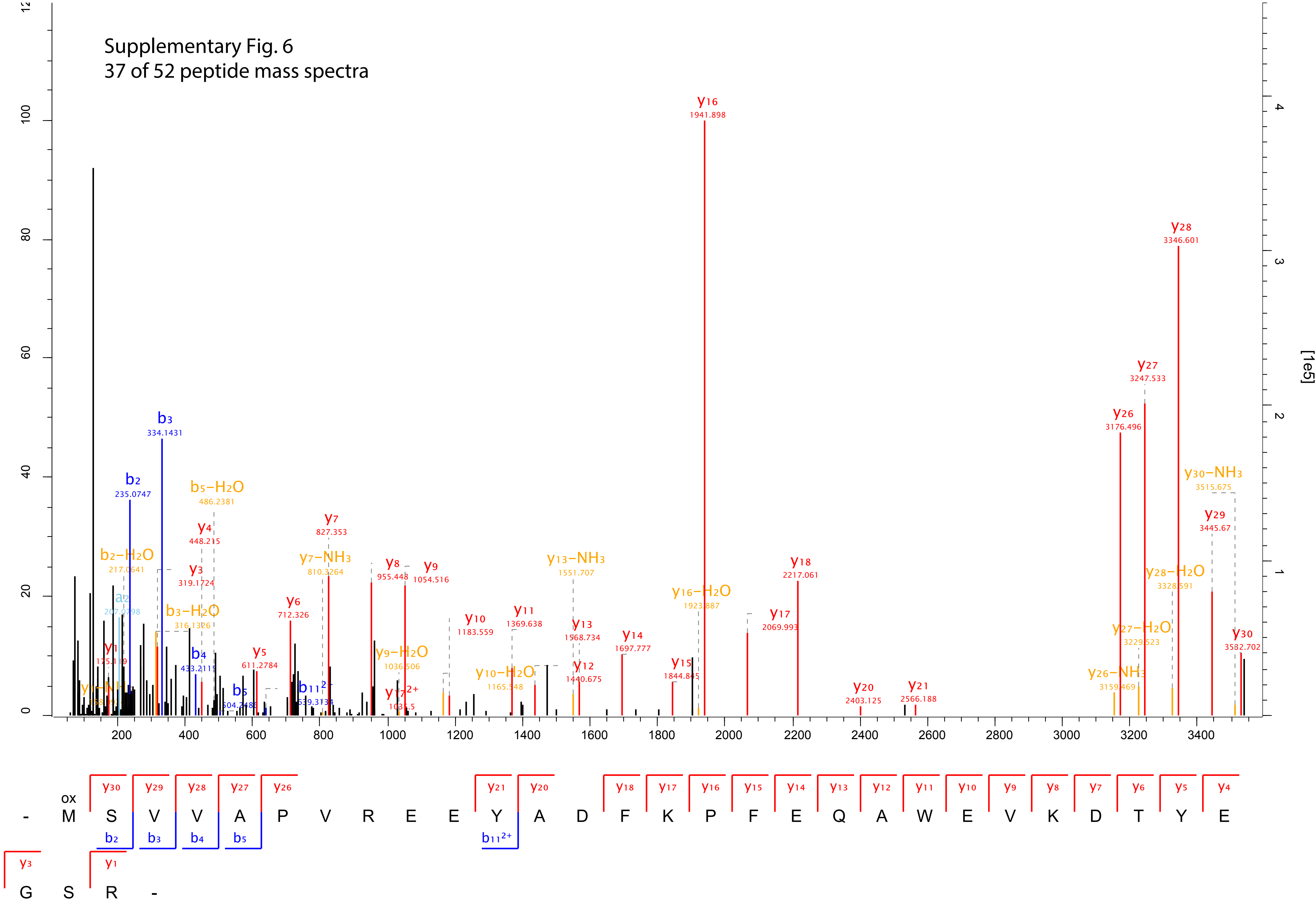

Raw file	Scan	Method	Score	m/z
180221_FS1_Li_DMo_cRNA_F_P	22954	FTMS; HCD	101.61	766.89

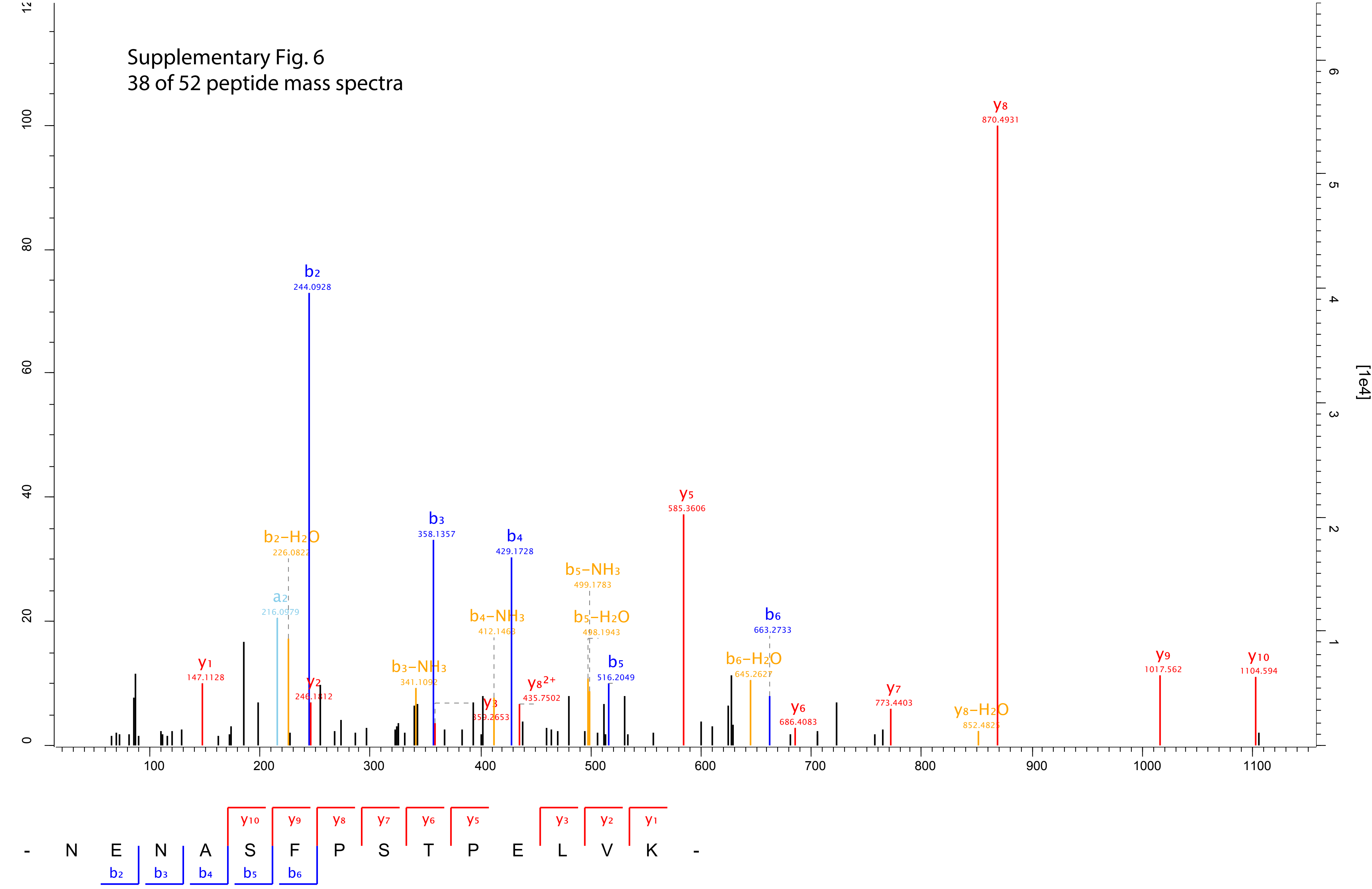

Supplementary Fig. 6
38 of 52 peptide mass spectra

Raw file	Scan	Method	Score	m/z
180221_FS1_Li_DMo_cRNA_F_P	19707	FTMS; HCD	145.91	650.33

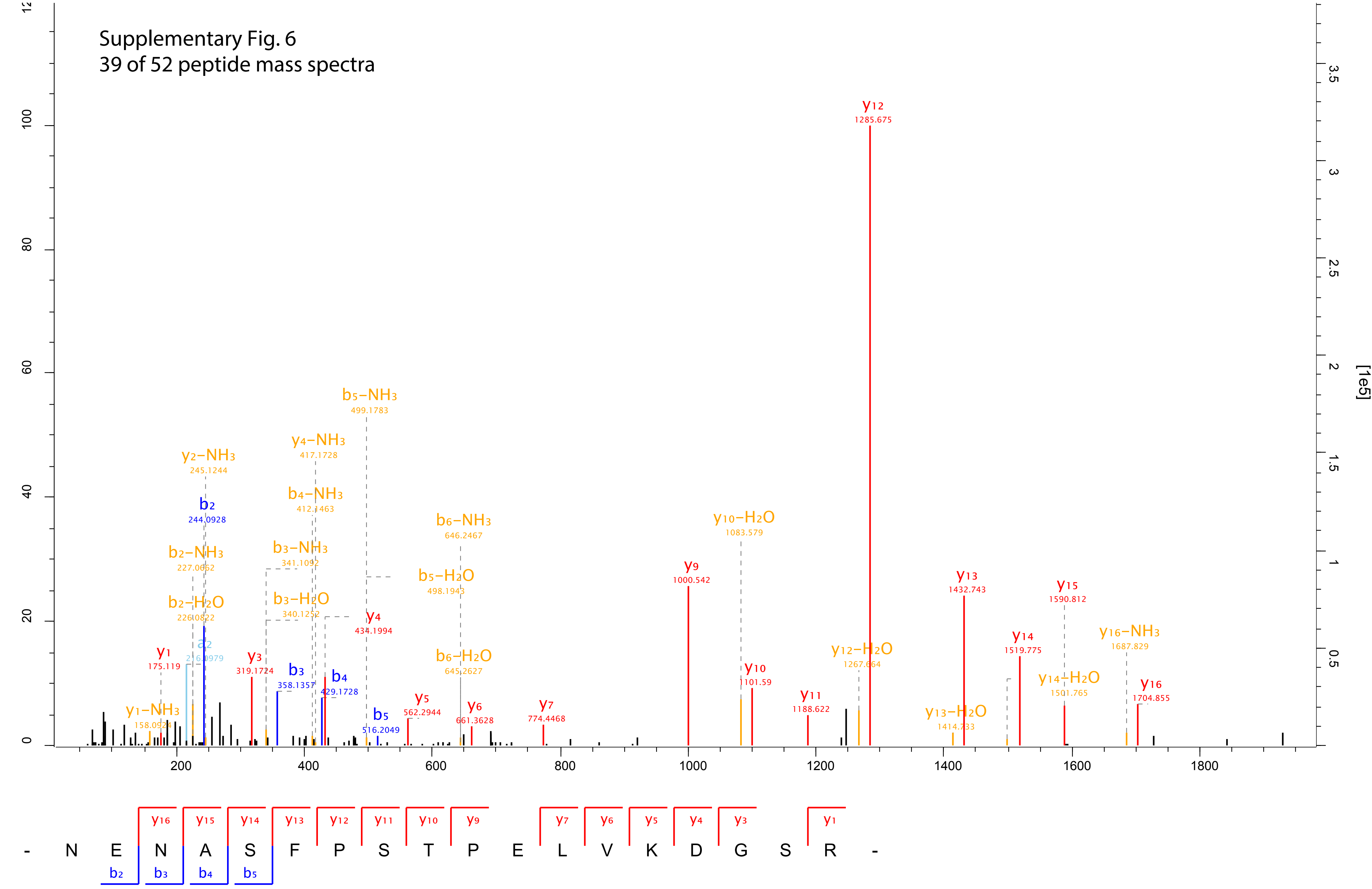

Raw file	Scan	Method	Score	m/z
180221_FS1_Li_DMo_cRNA_Try	11562	FTMS; HCD	202.18	623.29

Supplementary Fig. 6
40 of 52 peptide mass spectra

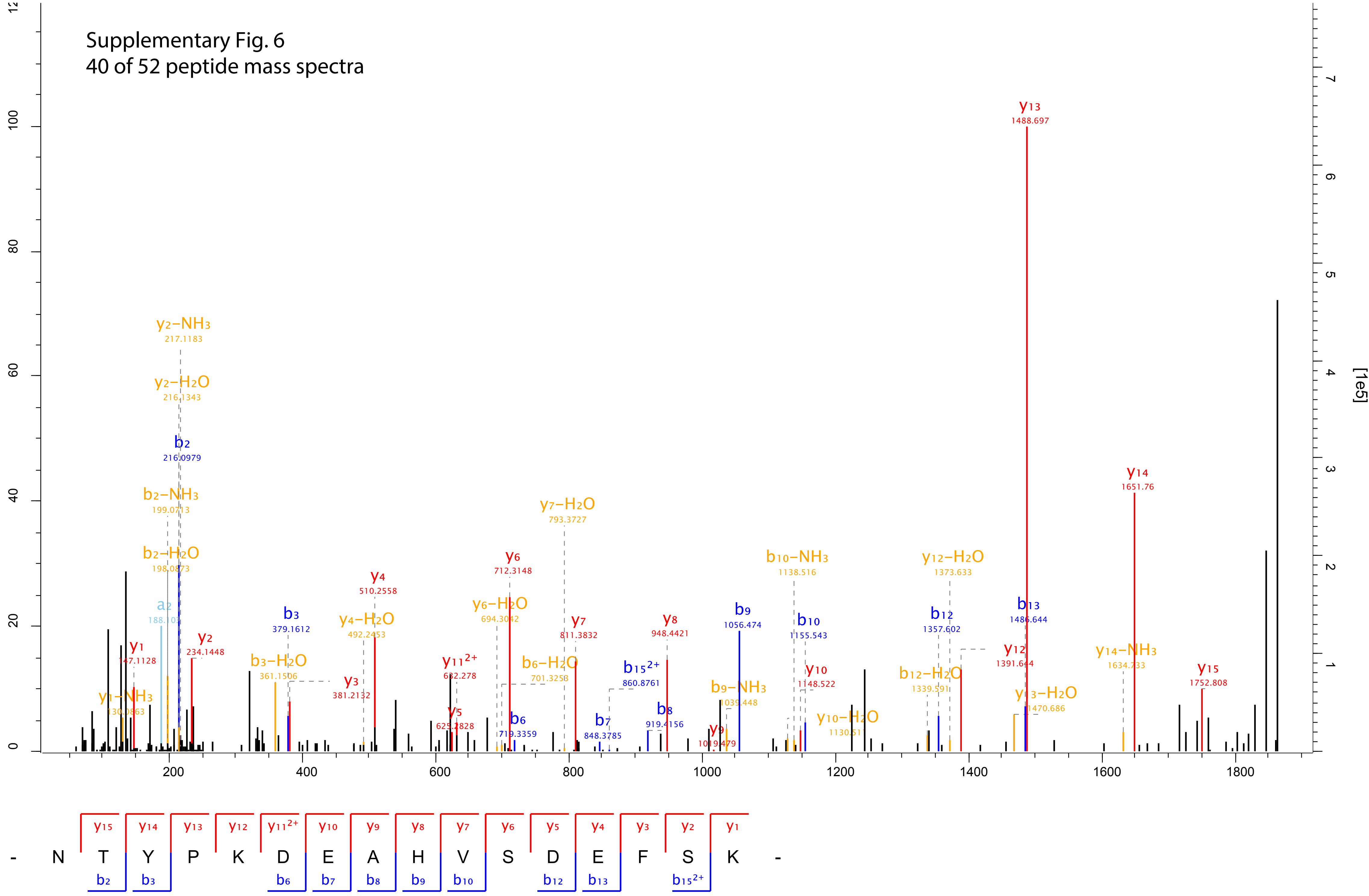

Raw file	Scan	Method	Score	m/z
180221_FS1_Li_DMo_cRNA_Try	34083	FTMS; HCD	50.27	909.44

Supplementary Fig. 6
41 of 52 peptide mass spectra

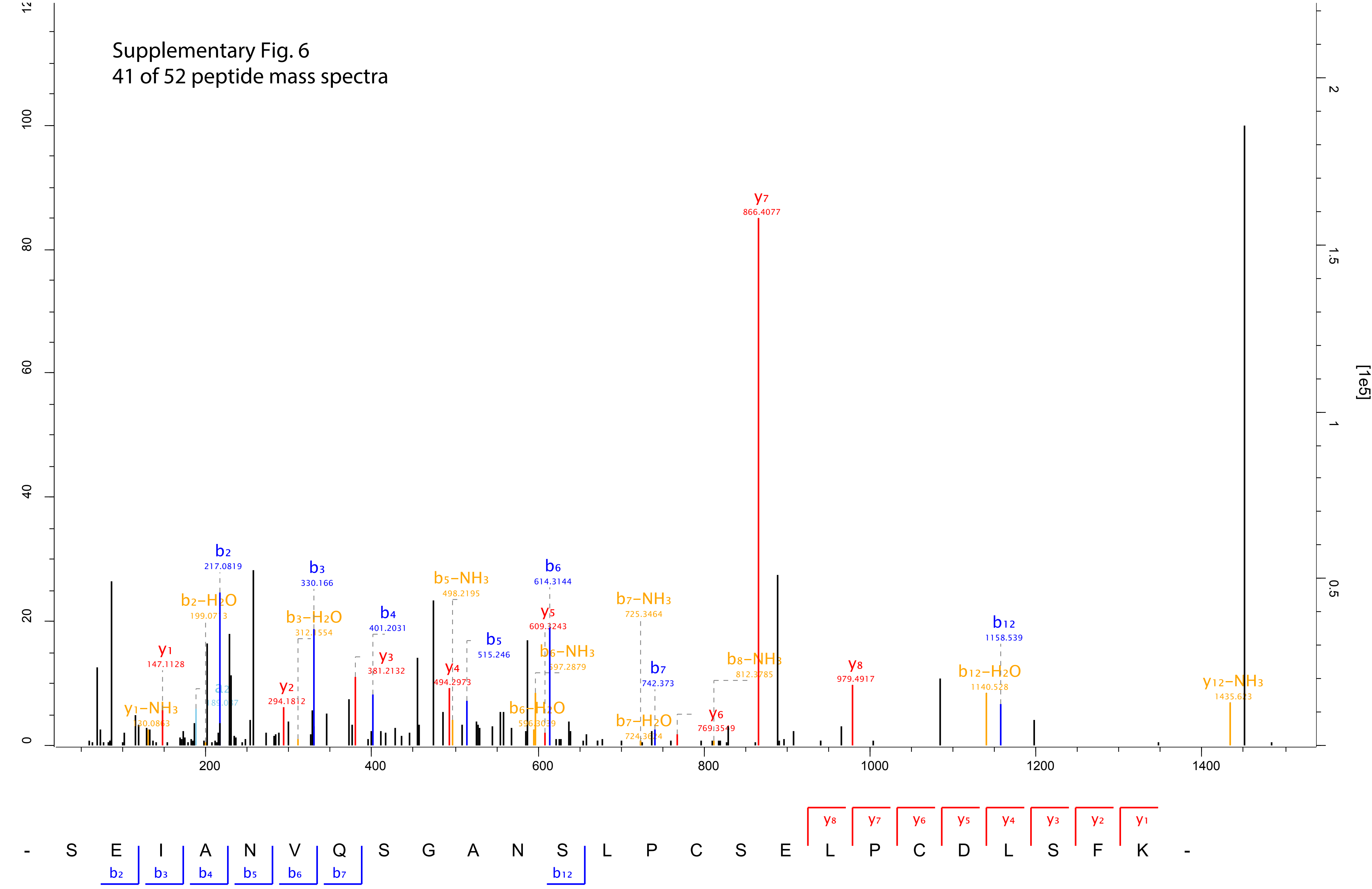

Raw file	Scan	Method	Score	m/z
180221_FS1_Li_DMo_cRNA_Try	15866	FTMS; HCD	102.45	630.06

Supplementary Fig. 6
42 of 52 peptide mass spectra

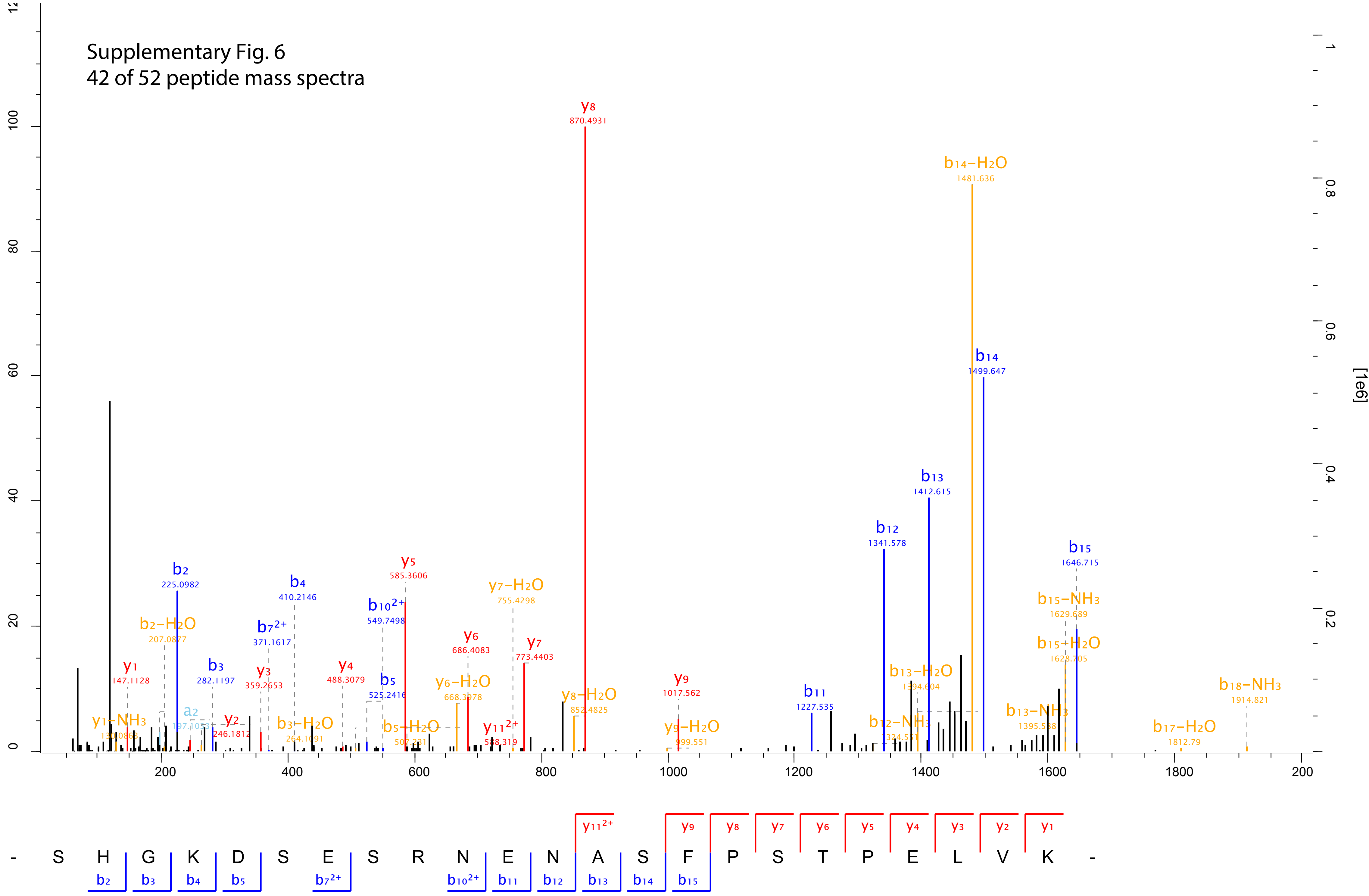

Raw file	Scan	Method	Score	m/z
180221_FS1_Li_DMo_cRNA_Try	18155	FTMS; HCD	182.95	686.1

Supplementary Fig. 6
43 of 52 peptide mass spectra

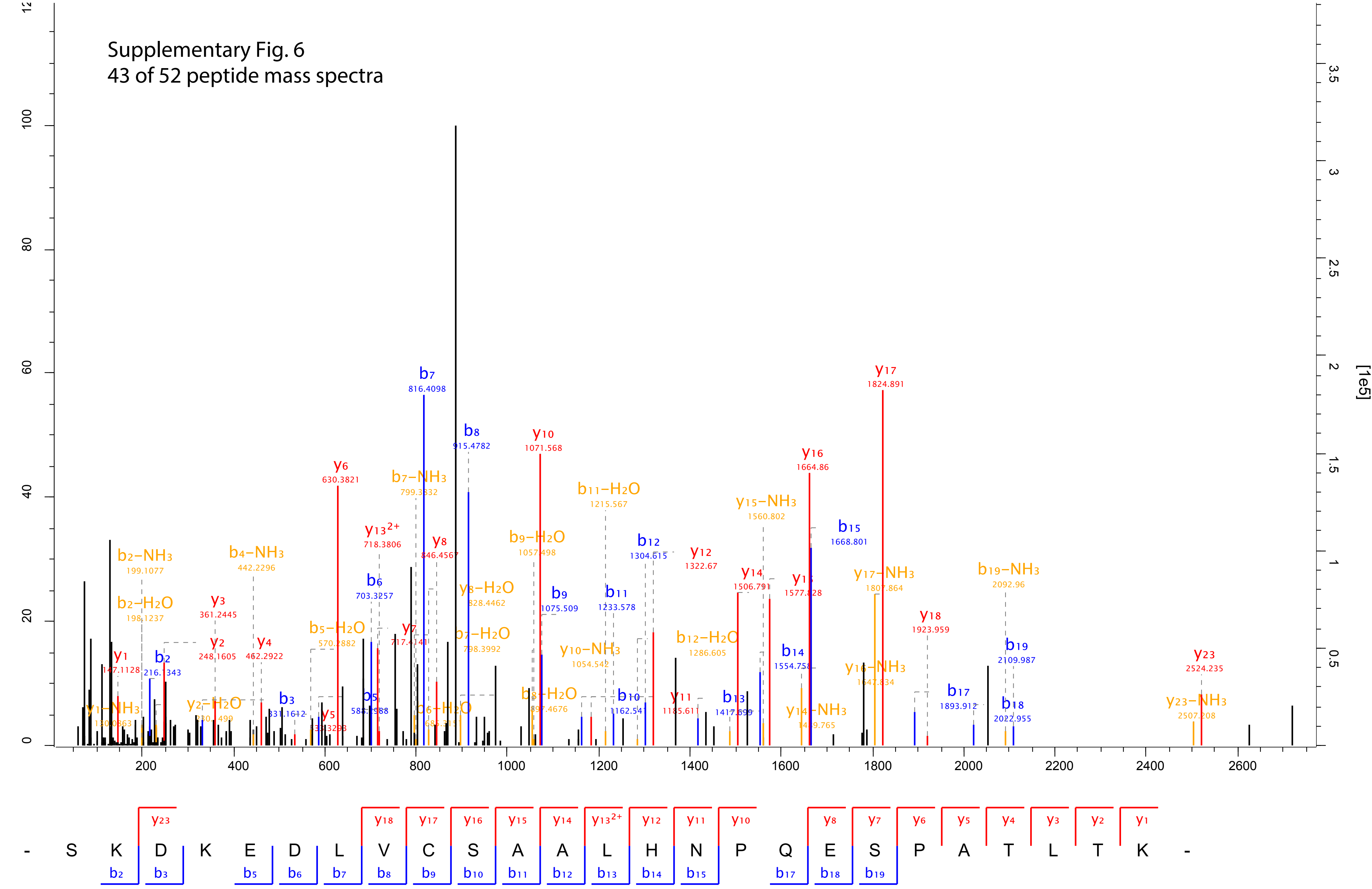

Raw file	Scan	Method	Score	m/z
180221_FS1_Li_DMo_cRNA_Try	30746	FTMS; HCD	178.71	623.36

Supplementary Fig. 6
44 of 52 peptide mass spectra

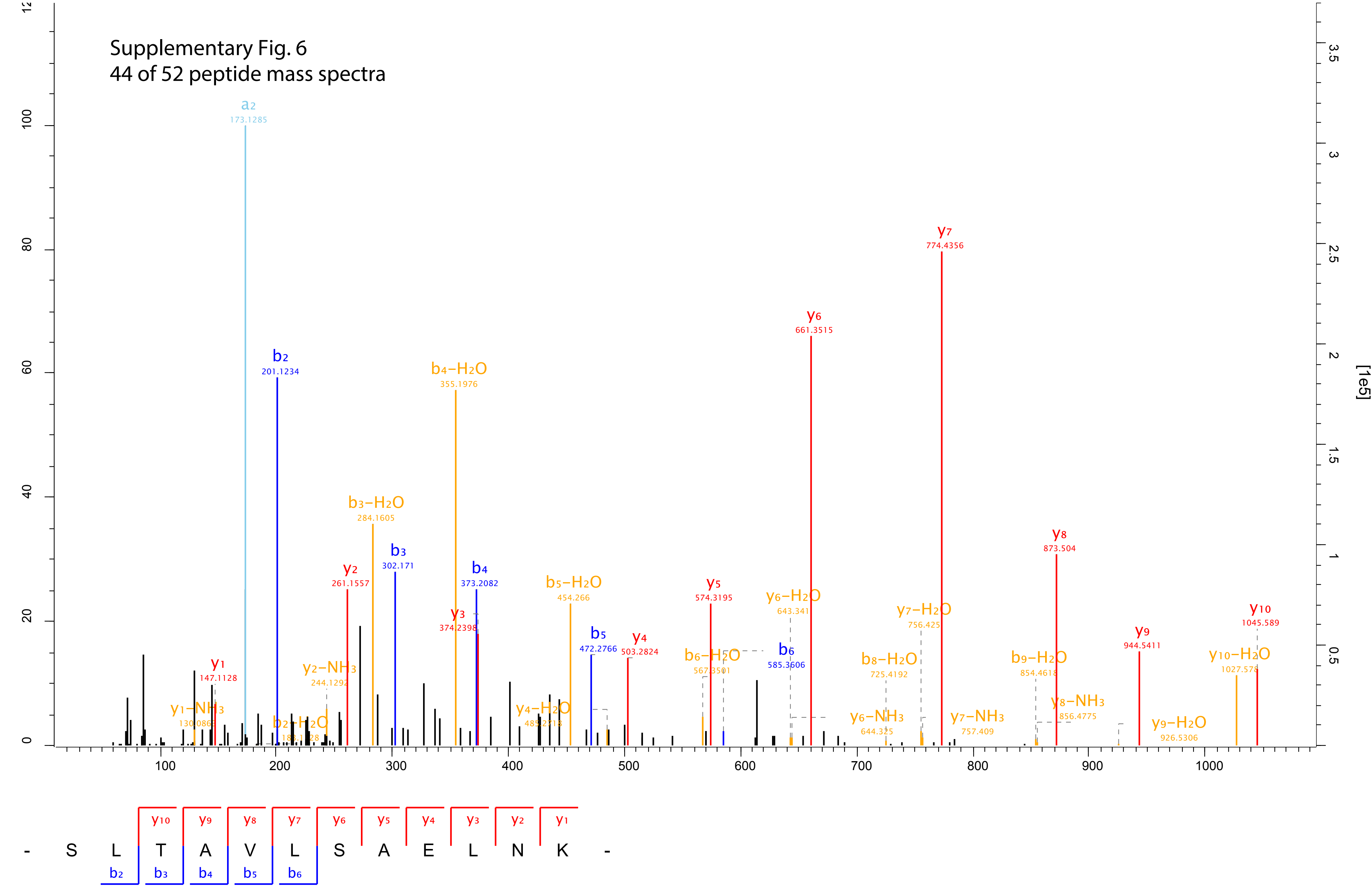

Raw file	Scan	Method	Score	m/z
180221_FS1_Li_DMo_cRNA_F_P	19442	FTMS; HCD	135.43	572.76

Supplementary Fig. 6
45 of 52 peptide mass spectra

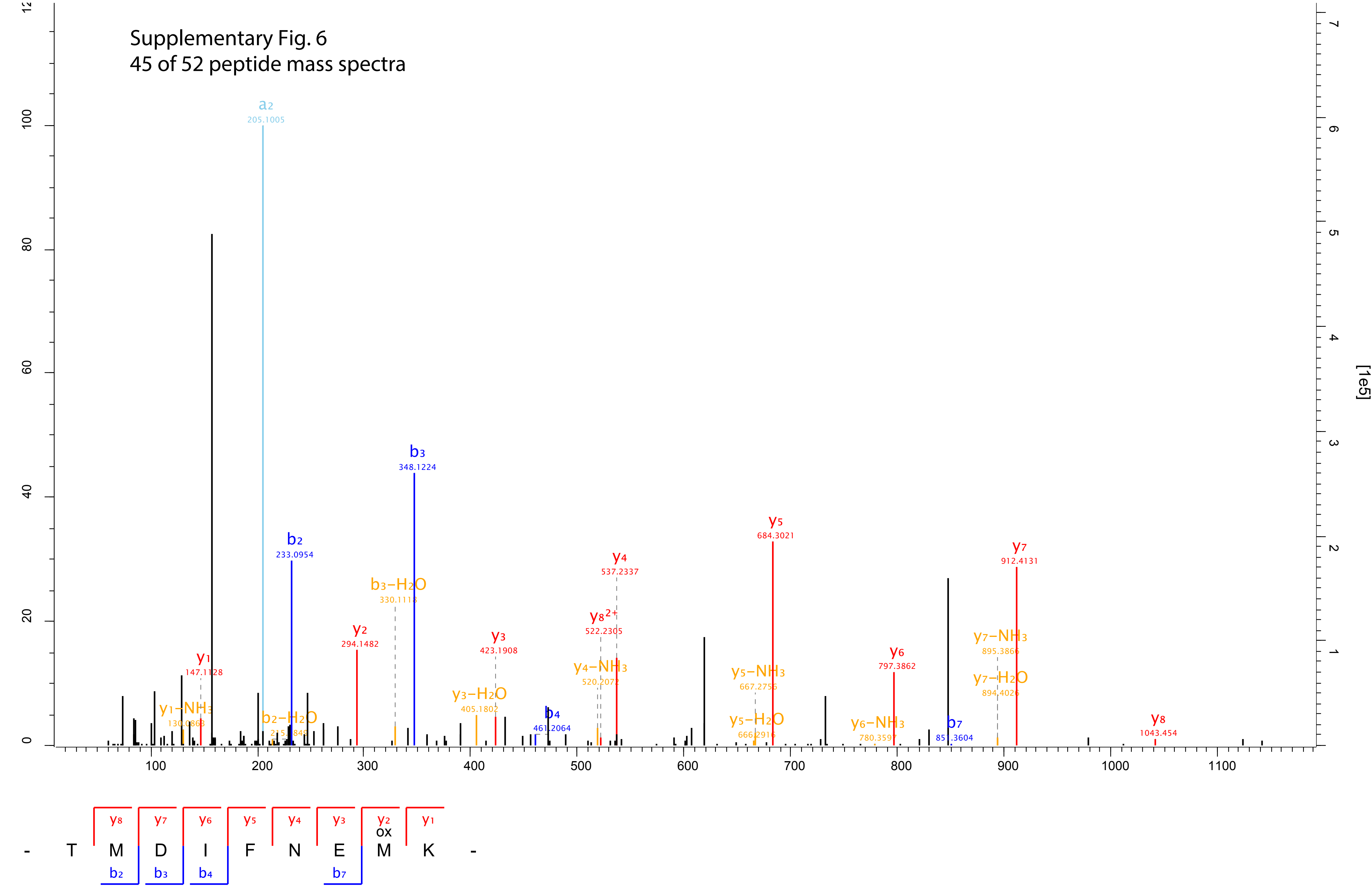

Raw file	Scan	Method	Score	m/z
180221_FS1_Li_DMo_cRNA_F_P	32825	FTMS; HCD	69.52	888.44

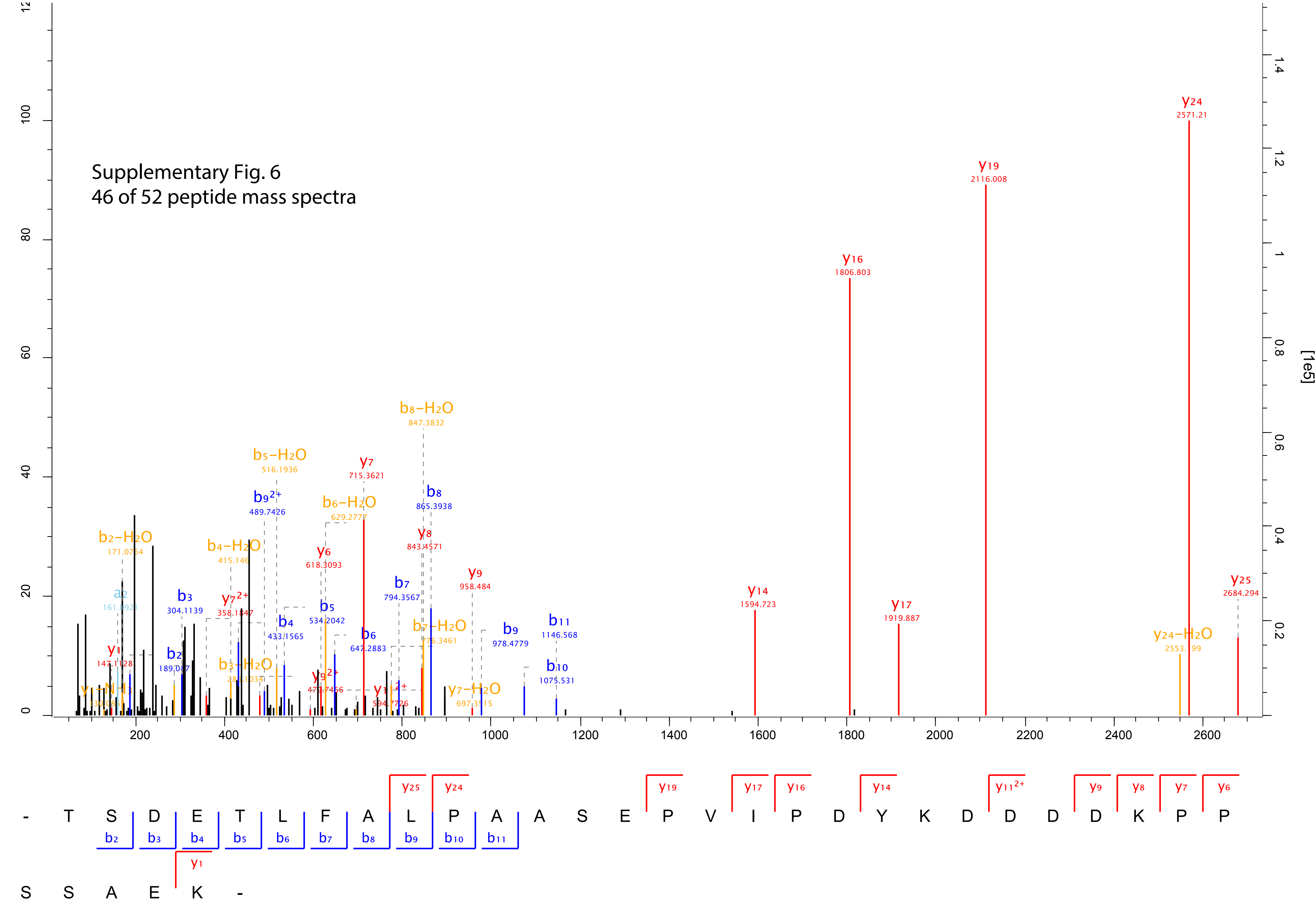

Raw file	Scan	Method	Score	m/z
180221_FS1_Li_DMo_cRNA_Try	34976	FTMS; HCD	84.67	880.1

Supplementary Fig. 6

47 of 52 peptide mass spectra

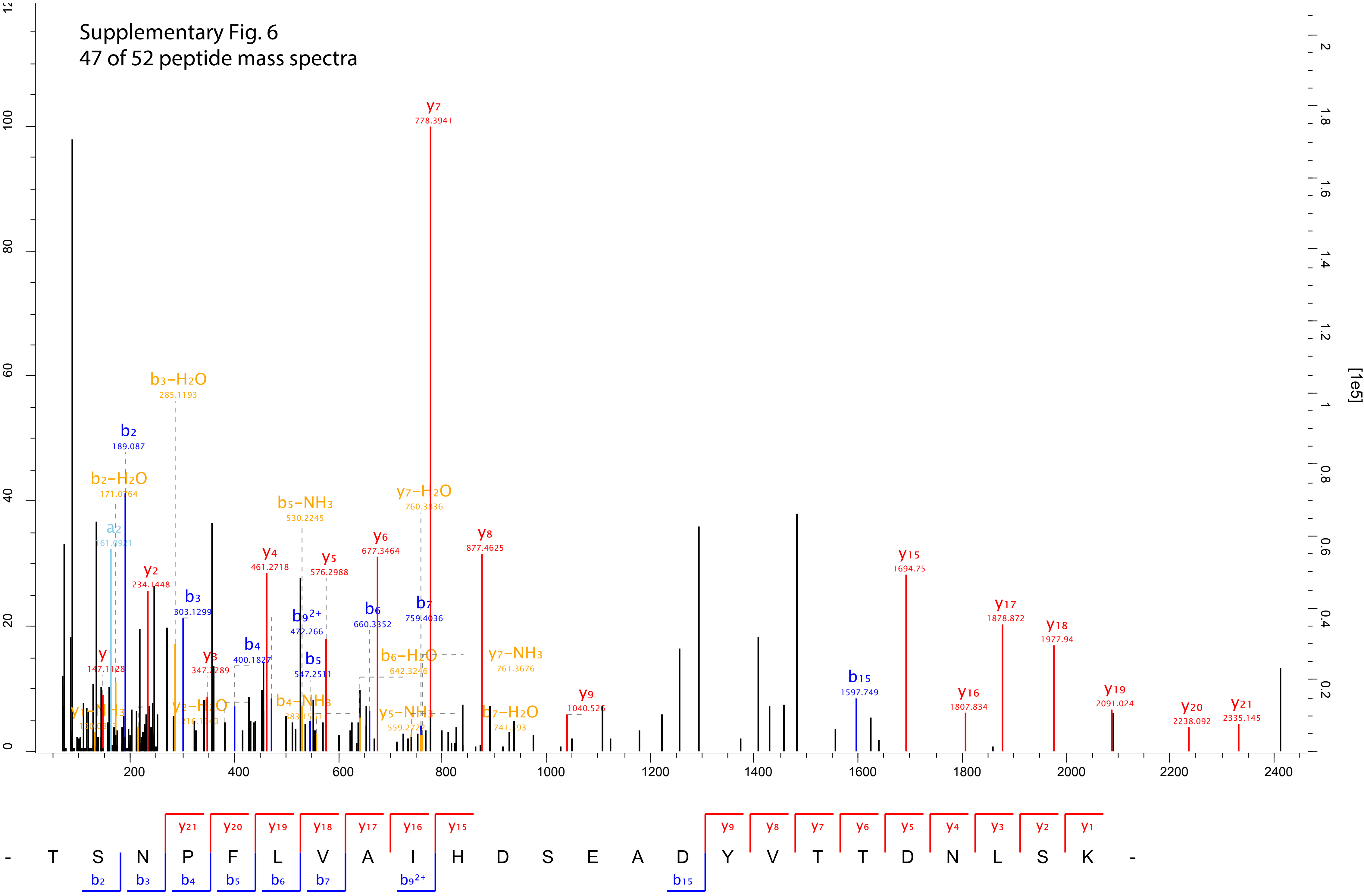

Raw file	Scan	Method	Score	m/z
180221_FS1_Li_DMo_cRNA_F_P	37725	FTMS; HCD	62.74	789.12

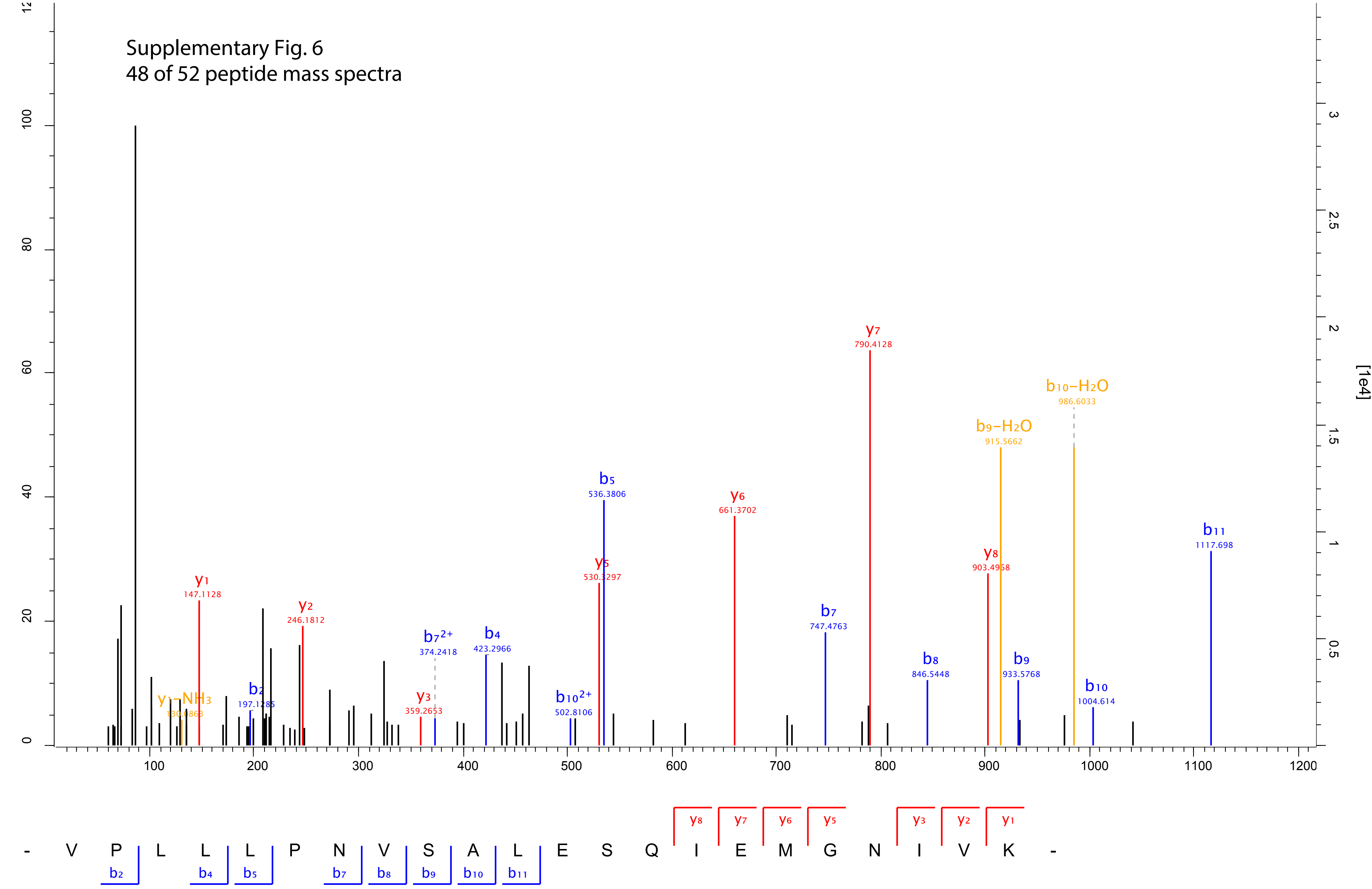

Raw file	Scan	Method	Score	m/z
180221_FS1_Li_DMo_cRNA_F_P	36128	FTMS; HCD	199.89	869.5

Supplementary Fig. 6
49 of 52 peptide mass spectra

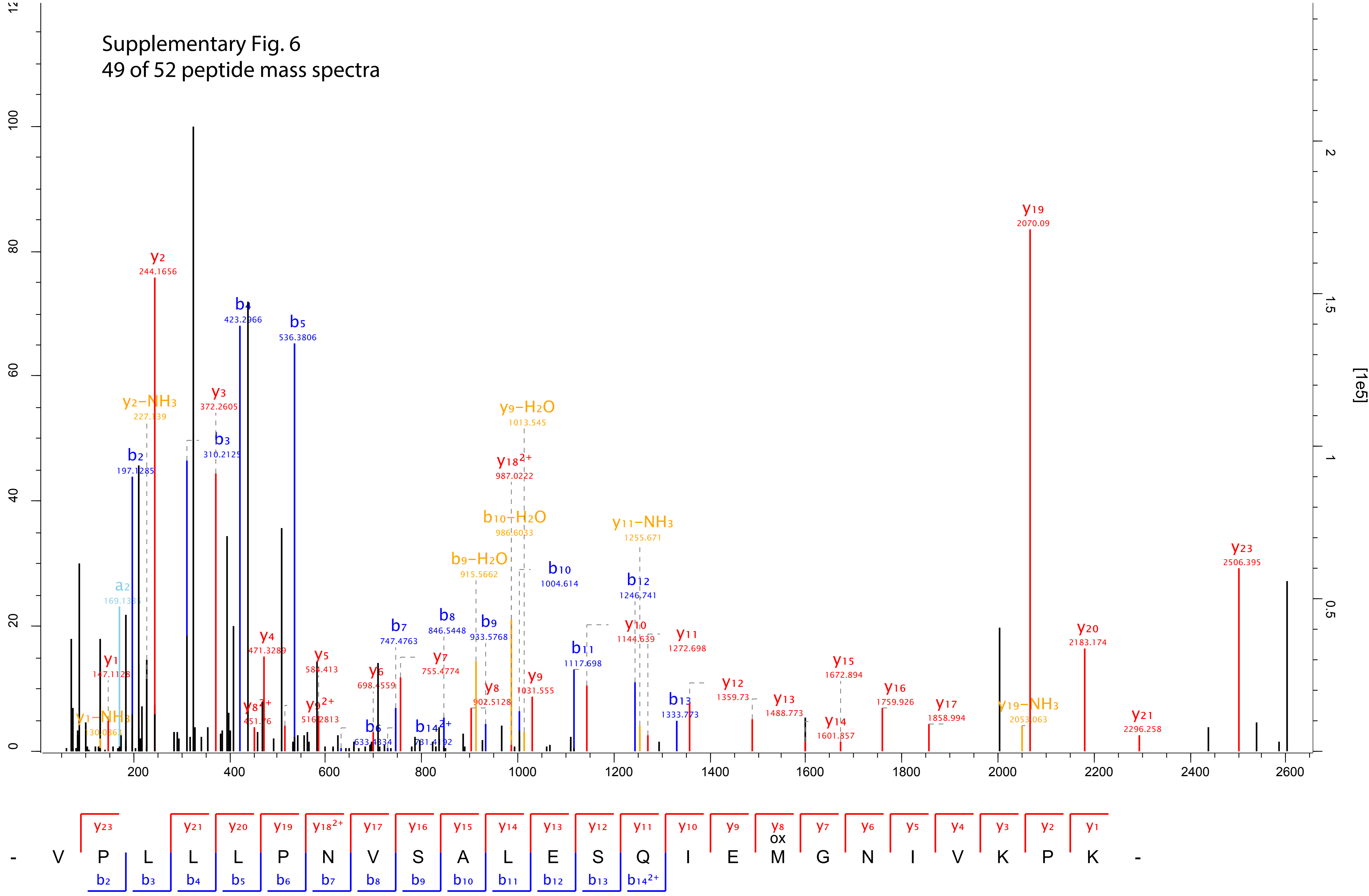

Raw file	Scan	Method	Score	m/z
180221_FS1_Li_DMo_cRNA_Try	36189	FTMS; HCD	53.52	1164.57

Supplementary Fig. 6
50 of 52 peptide mass spectra

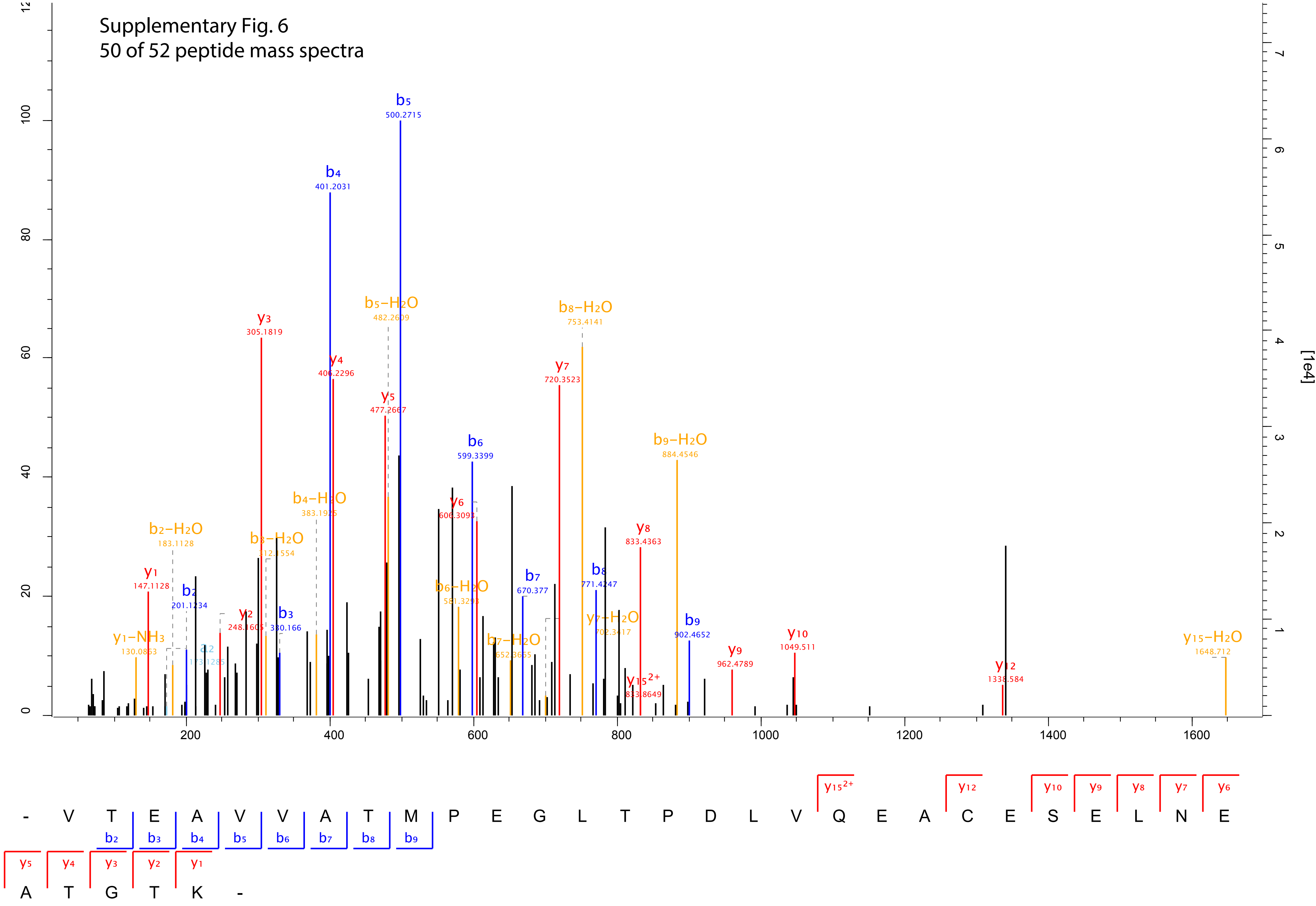

Raw file	Scan	Method	Score	m/z
180221_FS1_Li_DMo_cRNA_F_P	5595	FTMS; HCD	112.02	439.9

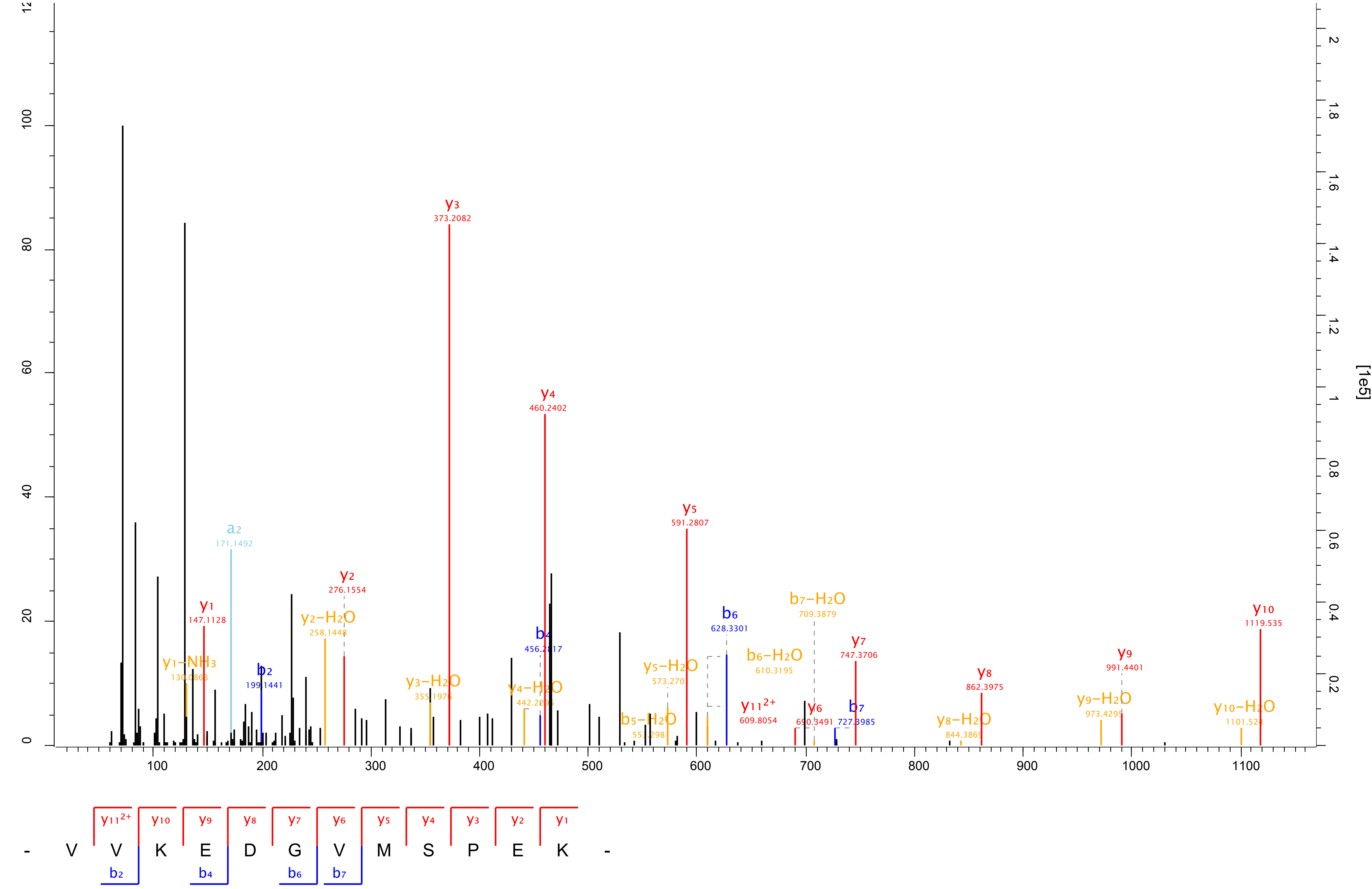

Supplementary Fig. 6
52 of 52 peptide mass spectra

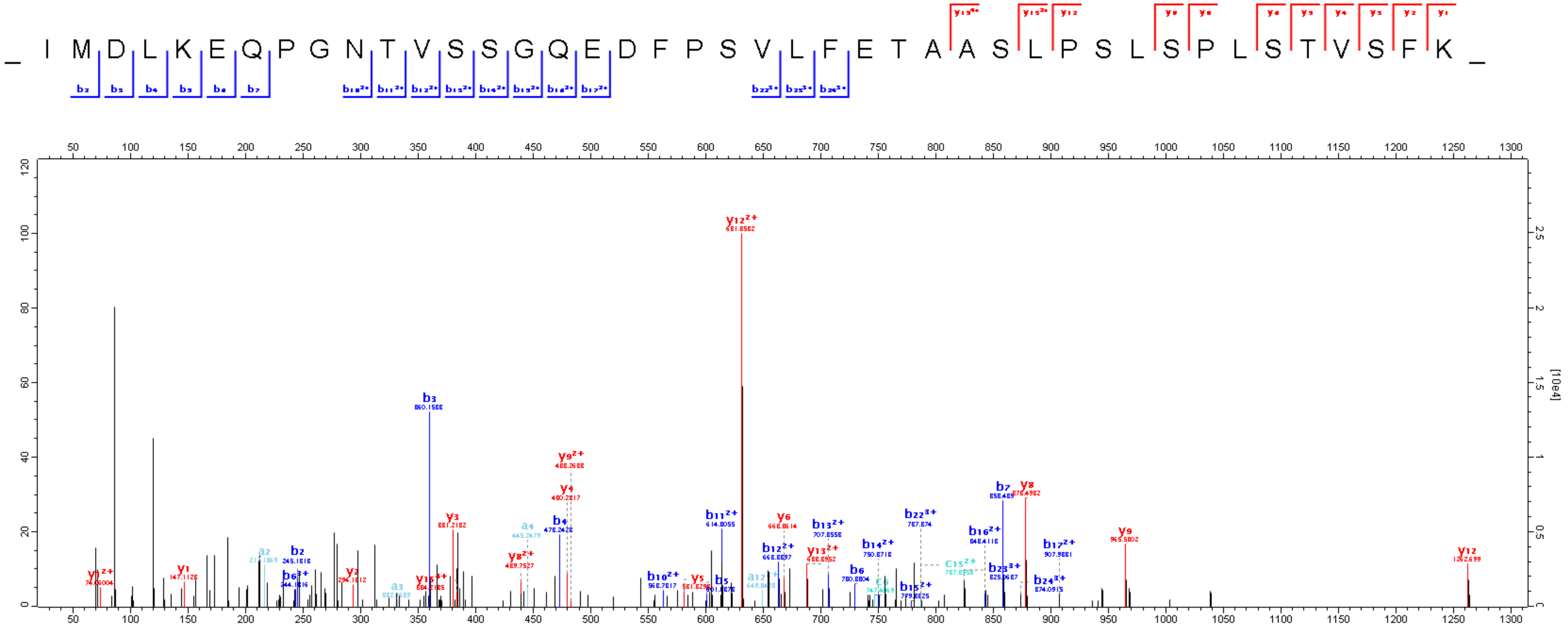

A

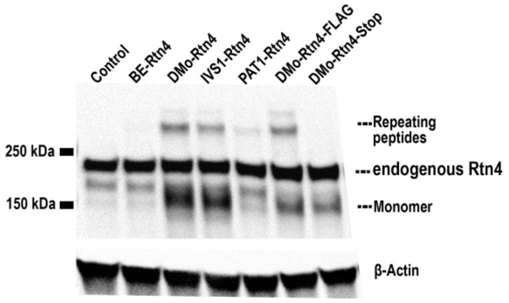

B

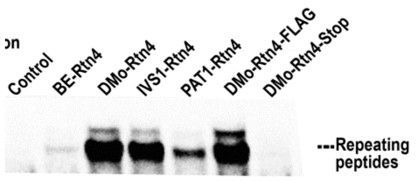

Supplementary Fig. 7.

Several IMEs promote circRtn4 translation in N2a cells.

A. Western blot with Anti-Nogo A antibody of protein isolated from N2a cells with circRtn4 overexpression. Control, empty vector; BE-Rtn4, pCircRNA-BE-Rtn4; DMo-Rtn4, pCircRNA-DMo-Rtn4; IVS1-Rtn4, pCircRNA-IVS1-Rtn4; PAT1-Rtn4, pCircRNA-PAT1-Rtn4; DMo-Rtn4-FLAG, pCircRNA-DMo-Rtn4-FLAG; DMo-Rtn4-Stop, pCircRNA-DMo-Rtn4-Stop; the high molecular weight bands larger than 250 kDa represent polypeptides from circRtn4 continuous translation; monomer designates the putative product from single round of circRtn4 translation; actin is used as loading control. B. long exposure of the high molecular weight bands larger than 250 kDa.

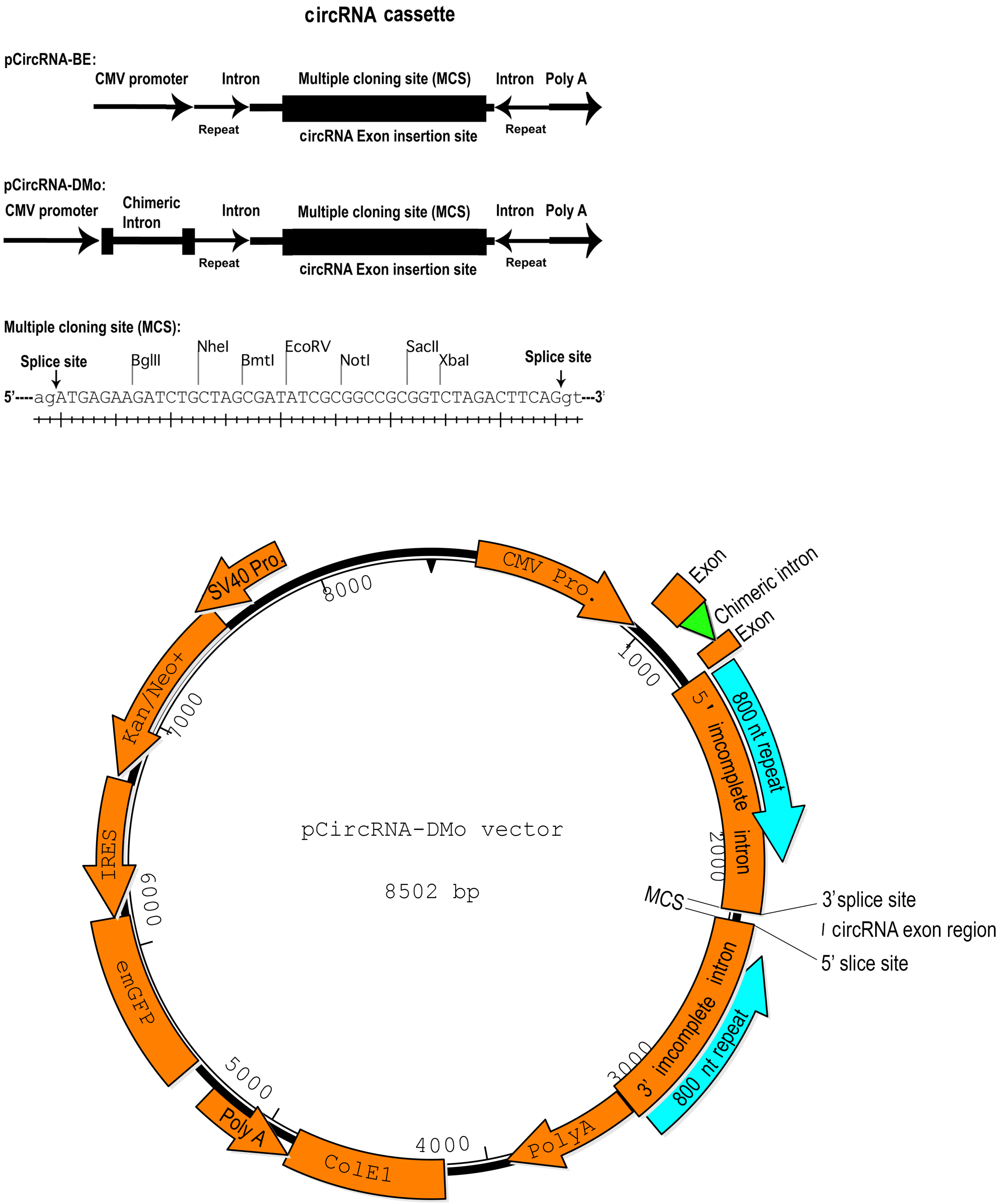

Supplementary Fig. 8 CircRNA expression cassette of pCircRNA-BE and pCircRNA-DMo vectors.

The multiple cloning site (MCS) contains the following restriction endonuclease recognition sites: *Bgl*II, *Nhe*I, *Bmt*I, *Eco*RV, *Not*I, *Sac*II, and *Xba*I. The 5' and 3' splice sites are indicated by lines. GFP of the original pCMV-MIR vector was replaced by emGFP (emerald green fluorescent protein).

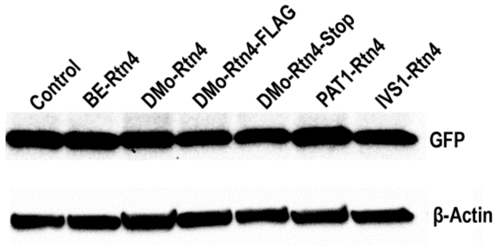

Supplementary Fig. 9 Equal transfection efficiency from circRNA vectors.
GFP expression from the SV40 promoter (Supplementary Fig. 1) in each circRNA expression plasmid DNA transfection in HEK293 cells was measured by Western blot analysis with antibody against GFP. β-Actin was used as loading control. Control, pCircRNA-DMo; BE-Rtn4, pCircRNA-BE-Rtn4; DMo-Rtn4, pCircRNA-DMo-Rtn4; DMo-Rtn4-FLAG, pCircRNA-DMo-Rtn4-FLAG; DMo-Rtn4-Stop, pCircRNA-DMo-Rtn4-Stop; PAT1-Rtn4, pCircRNA- PAT1-Rtn4; IVS1-Rtn4, pCircRNA-IVS1-Rtn4.
